# Supplementary material for: A cage-on-MOF strategy to coordinatively functionalize mesoporous MOFs for manipulating selectivity in adsorption and catalysis
Source: Nat Commun. 2023 Aug 26;14:5223. doi: 10.1038/s41467-023-40973-9 (PMC10460432; doi:10.1038/s41467-023-40973-9)
Supplement: Supplementary file 1 — Supplementary Information [file 41467_2023_40973_MOESM1_ESM.pdf]

Supplementary Information

A Cage-on-MOF Strategy to Coordinatively Functionalize Mesoporous MOFs for Manipulating Selectivity in Adsorption and Catalysis

Yu Liang[1,2]†, Xiaoxin Yang[1,3,]†, Xiaoyu Wang[4], Zong-Jie Guan[1,2], Hang Xing[1,3*], Yu Fang[1,2*]

[1] State Key Laboratory for Chemo/Bio-Sensing and Chemometrics, College of Chemistry and Chemical Engineering, Hunan University, Changsha 410082, Hunan, China

[2] Innovation Institute of Industrial Design and Machine Intelligence Quanzhou-Hunan University, Quanzhou 362801, Fujian, China

[3] Institute of Chemical Biology and Nanomedicine, Hunan University, Changsha 410082, Hunan, China

[4] Kuang Yaming Honors School, Nanjing University, Nanjing 210023, China

† These authors contributed equally.

* To whom correspondence should be addressed.

Email: hangxing@hnu.edu.cn, yu.fang@hnu.edu.cn

Content

Materials 3

Characterization 4

Section 1. Synthesis of Ligands and Complexes 8

Section 2. The crystal structure of PCCs 10

Section 3. Characterizations of PCN-222@PCCs 17

Section 4. Theoretical Calculations of PCN-222@PCCs 36

Section 5. Dye adsorptions of PCN-222@PCCs 38

Section 6. Catalytic reactions of PCN-222@PCCs 47

Section 7. Characterizations of MIL-101@PCCs 58

Section 8. Theoretical Calculations of MIL-101@PCCs 79

Section 9. Dye adsorptions of MIL-101@PCCs 81

Section 10. Catalytic reactions of MIL-101@PCCs 91

Materials

All solvents and reagents obtained from commercial sources are used without further purification. Tetrakis (4-carboxyphenyl)-porphyrin (H_4TCPP, 97%), zirconyl chloride octahydrate ($ZrOCl_2·8H_2O$, 98%), terephthalic acid (BDC, 99%), chromium chloride ($CrCl_3·6H_2O$, 99%), cobalt chloride hexahydrate ($CoCl_2·6H_2O$, 99%) are purchased from Admas-beta. 2, 2-bipyridine (99%), carbon load palladium, Sodium chloride (99%), sodium hydroxide (99%), trifluoroacetic acid (99%), potassium hydroxide (99%), P-tert-butyl phenol (99%) are obtained from MACHLIN. Palladium chloride ($PdCl_2$, 99%), acetonitrile (99%), 2,4,6-Tris(2-pyridyl)-s-triazine (TPT, 99%), eosin Y, methylene blue, rhodamine B, methyl orange, benzaldehyde dimethyl acetal (98%), malononitrile (99%), methanol (99%), ethanol (99%), benzaldehyde (99%), N, N-dimethylformamide (DMF, 99%), acetic acid (99%) Triethylamine (99%), benzylamine (99%), tetraethylene glycol dimethyl ether (99%) are obtained from Shanghai Titan Scientific. Silver hexafluorophosphate ($AgPF_6$, 98%) is purchased from Sigma-Aldrich. Hydrochloric acid (36~38%), sulfuric acid (98%), nitric acid (65~68%), hydrogen peroxide (30%), acetone (99%), hydrazine hydrate (45%), sulfur powder, diethyl ether (99%), and toluene (99%) are obtained from Sinopharm Chemical Reagent Co. LTD.

Characterizations

Scanning electron microscopy (SEM)

Scanning electron microscopy images were acquired on JSM-7610FPlus. The samples to be tested can be obtained by dispersing the appropriate amount of samples in ethanol and then dropping them on silicon wafers to dry.

Transmission electron microscopy (TEM)

Scanning electron microscopy images were acquired on FEI Tecnai F20. The samples to be tested can be obtained by dispersing the appropriate number of samples in ethanol and then dropping them on a thin carbon film to dry.

High-angle annular dark-field scanning transmission electron microscope (HAADF-STEM)

STEM images and linear scanning results (Figure 6e, Supplementary Figs. 10 and 77) were acquired using a double spherical aberration-corrected STEM/TEM instrument, specifically the FEI Titan G2 60-300 in Nanjing University, operating at a voltage of 300 kV and equipped with a field emission gun. The probe convergence angle on the Titan electron microscope was set at 22.5 mrad, while the high-angle annular dark-field (HAADF) detector covered an angular range from 79.5 to 200 mrad.

Other STEM images were acquired on FEI Tecnai F20. The samples to be tested can be obtained by dispersing the appropriate number of samples in ethanol and then dropping them on a thin carbon film to dry.

Fourier transform infrared (FT-IR)

Infrared was used to detect the stretching vibration characteristic of PCC in the samples.

The FT-IR spectra were obtained using IR-Spirit-T (206-31010-58).

X-ray photoelectron spectroscopy (*XPS*)

X-ray photoelectron spectroscopy (*XPS*) data were determined by Thermo Kalpha. The binding energy was adjusted by setting the C1s transition to 284.6 eV.

Powder X-ray diffraction (PXRD)

Powder X-ray diffraction patterns (PXRD) were acquired on a Bruker D8 Advance diffractometer with Cu Kα radiation.

Nitrogen adsorption measurements

N_2 adsorption isotherms were collected on a BSD-660S-0005. Prior to analyses, samples were activated under a vacuum for 12 hours at 150 ºC. A liquid nitrogen bath (77K) was used for the measurement.

Zeta potential

Zeta potential was performed on a Malvern Zetasizer Nano ZS. An appropriate number of samples should be sonicated and dispersed in deionized water before testing.

Inductively coupled plasma optical emission spectrometer (ICP-OES)

ICP-OES were determined by Agilent 720ES. Prior to analyses, the samples were soaked overnight in a mixture of hydrogen peroxide and sodium hydroxide and then dissolved by adding aqua regia.

Ultraviolet-visible spectroscopy (UV-Vis)

UV-vis spectroscopy was performed on a UV-1900i. The test results were used to measure the change of dye adsorption behavior of MOF@PCC.

Dye adsorption of PCN-222 and PCN-222@PCCs

Eosin Y with concentrations of 10, 20, 30, 40 and 50 mg/L and methylene blue with concentrations of 10, 20, 30, 40 and 50 mg/L were prepared, respectively. A 20 ml of dye solution was used and 2 mg of adsorbent was added. After 2 hours, the supernatant was tested to compare the adsorption capacity of different materials.

Eosin Y (30 mg/L) and methylene blue (10 mg/L) were respectively prepared. A 20 ml of dye solution was used and 2 mg of adsorbent was added, and the supernatant was tested after a period of time to compare the adsorption rates of different materials.

Prepare a mixed aqueous solution with both EY and MB concentrations of 12.5 mg/L, and then 1.5 mg of adsorbent was added to 40 ml of the prepared solution, and the supernatant of the solution was subjected to UV detection at regular intervals.

Dye adsorption of MIL-101 and MIL-101@PCCs

Rhodamine B with concentrations of 20, 40, 60, 80 and 100 mg/L and methyl orange with concentrations of 20, 40, 60, 80 and 100 mg/L were prepared, respectively. A 20 ml of dye solution was used, and 5 mg of adsorbent was added. After 2 hours, the supernatant was tested to compare the adsorption capacity of different materials.

Rhodamine B (20 mg/L) and methyl orange (60 mg/L) were respectively prepared. A 20 ml of dye solution was used and 5 mg of adsorbent was added, and the supernatant was tested after a period of time to compare the adsorption rates of different materials.

Prepare a mixed aqueous solution with both Rhodamine B (120 mg/L) and methyl orange (60 mg/L), and then 5 mg of adsorbent was added to 20 ml of the prepared solution, and the supernatant of the solution was subjected to UV detection at regular intervals.

Section 1. Synthesis of Ligands and Complexes

Synthesis of PCN-222

The parent PCN-222 was synthesized by the modified procedure published previously. 150 mg $ZrOCl_2 \cdot 8H_2O$ (0.465 mmol) was dissolved in 40 mL of DMF by 30 min sonication. Then, 116 mg of TPPC (0.147 mmol) was added and the solution was further sonicated for 10 min followed by addition of acetic acid (20 mL, 90%). The mixture was heated at 130 °C for 2 days. The resulting precipitate was centrifuged and washed three times with DMF and three times with ethanol. The obtained purple solid was air-dried at room temperature.

Synthesis of MIL-101

Terephthalic acid (36.615 mg, 0.22 mmol) and chromium chloride (53.846 mg, 0.20 mmol) hexahydrate were dispersed in 2 mL of water. The mixture system sealed in a 50 mL of vial, then heated at 180 °C for 3 h. After cooling to room temperature, the mixture was filtered to remove the recrystallized terephthalic acid. MIL-101 was purified by centrifugation and washed with DMF and ethanol. The pure product is dispersed in ethanol for later use.

Synthesis of PCC-4

Cobalt chloride hexahydrate (23.7 mg, 0.1 mmol), 2,4, 6-tri (4-carboxyl phenyl)-1,3, 5-triazine (14.5 mg, 0.33 mmol) and sodium 4-sulfonate-sulfonylcalix[4]arene (18 mg, 0.015 mmol) were suspended in 2 mL MeOH. The mixture was heated up at 85 °C in an oven for 12 h. After cooling down to ambient temperature, big purple crystals were collected and washed by methanol. (Sodium 4-sulfonate-sulfonylcalix[4]arene was synthesized according to previous reports in the literature.)

Synthesis of Pd(4,4'-diamino-2,2'-bipyridine)(PF_6)$_2$

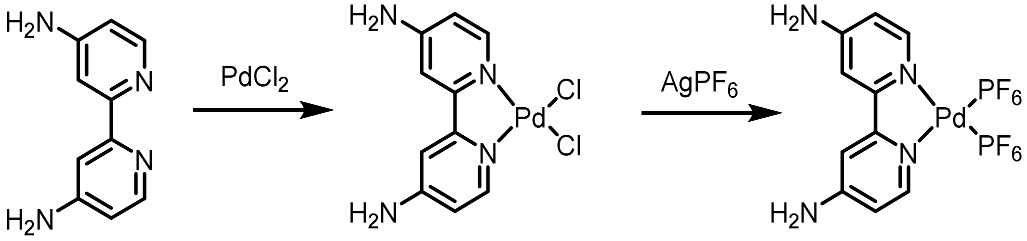

1 2

To a refluxing solution of $PdCl_2$ (35.5 mg, 0.20 mmol) in CH_3CN (15.0 mL) was added 4,4'-diamino-2,2'-bipyridine **1** (37.6 mg, 0.20 mmol) which was synthesized as previously reported. The resulting yellowish solution was stirred at 75 °C for another 4 h. Then the solution was cooled down to room temperature and treated with $AgPF_6$ (55 mg, 0.40 mmol). A large amount of white precipitate appeared instantly, and the suspended solution was stirred at room temperature for overnight. White precipitate was filtrated, and the clear orange solution was evaporated to dryness to obtain compound **2**.

Section 2. The crystal structure of PCCs

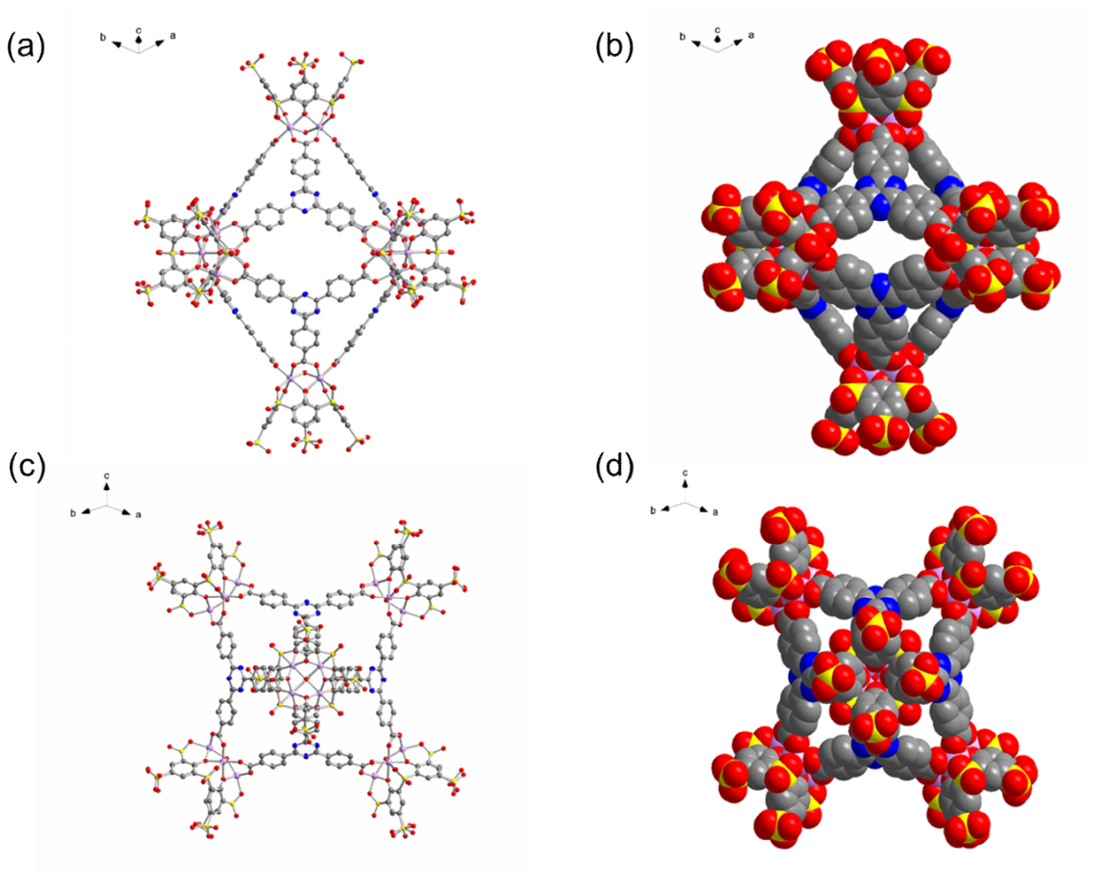

Supplementary Figure 1. Single crystal X-ray structure of **PCC-4**. (a) (c) Ball-and-stick model. (b) (d) Space-filling Model. (Red: oxygen atom; Gray: carbon atoms; Blue: nitrogen atoms; Yellow: sulfur atoms; Purple: cobalt atom; Hydrogen atoms are omitted for clarity).

Supplementary Table 1. Unit cell information of the **PCC-4.**

Empirical formula	$C_{336}H_{144}Co_{24}N_{24}O_{198}S_{48}$
Formula weight	10637.94
Temperature/K	293(2)
Crystal system	trigonal
Space group	*R*-3
a/Å	33.2626(12)
b/Å	33.2626(12)
c/Å	81.477(12)
α/°	90
β/°	90
γ/°	120
V/Å^3	78069(13)
Z	3
ρ_{calc} g/cm^3	0.679
μ/mm^{-1}	0.510
F(000)	15984.0
Radiation	MoKα (λ = 0.71073)
2Θ range for data collection/°	4.242 to 30.958
Index ranges	$-24 \le h \le 24$, $-24 \le k \le 24$, $-61 \le l \le 61$
Reflections collected	231230
Independent reflections	7645 [R_{int} = 0.3143, R_{sigma} = 0.0590]
Data/restraints/parameters	7645/928/982
Goodness-of-fit on F^2	1.059
Final *R* indexes [I>=2*σ* (I)]	R_1 = 0.1724, wR_2 = 0.3731
Final *R* indexes [all data]	R_1 = 0.1760, wR_2 = 0.3751
Largest diff. peak/hole / e Å^{-3}	0.55/-0.77

Crystal Data for $C_{336}H_{144}Co_{24}N_{24}O_{198}S_{48}$ (M =10637.94 g/mol): trigonal, R-3, a = 33.2626(12) Å, c = 81.477(12) Å, V = 78069(13) Å^3, Z = 3, T = 293(2) K, 231230 reflections measured (4.242° ≤ 2θ ≤ 30.958°), 7645 unique (Rint = 0.3143, Rsigma = 0.0590) which were used in all calculations. The final R1 was 0.1724 (I > 2σ(I)) and wR2 was 0.3751 (all data).

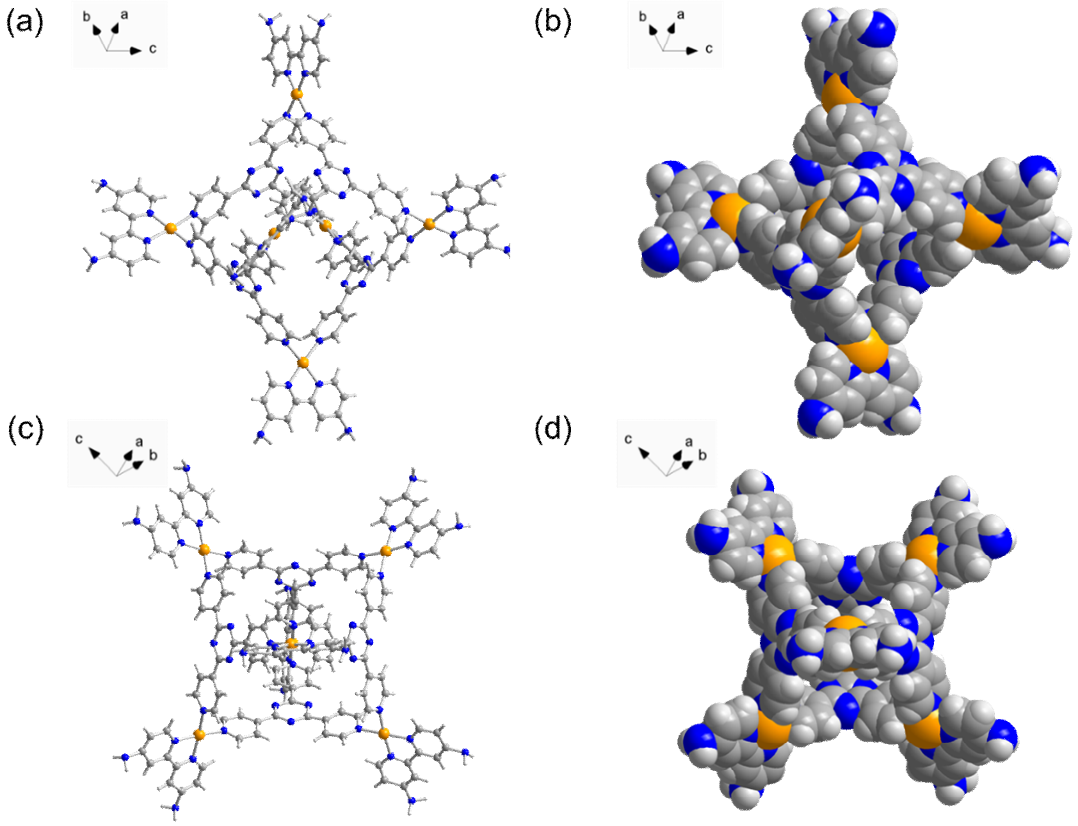

Supplementary Figure 2. Molecular modeling structure of **PCC-5**. (a) (c) Ball-and-stick model. (b) (d) Space-filling Model. (Orange: palladium atom; Gray: carbon atoms; Blue: nitrogen atoms; White: hydrogen atoms).

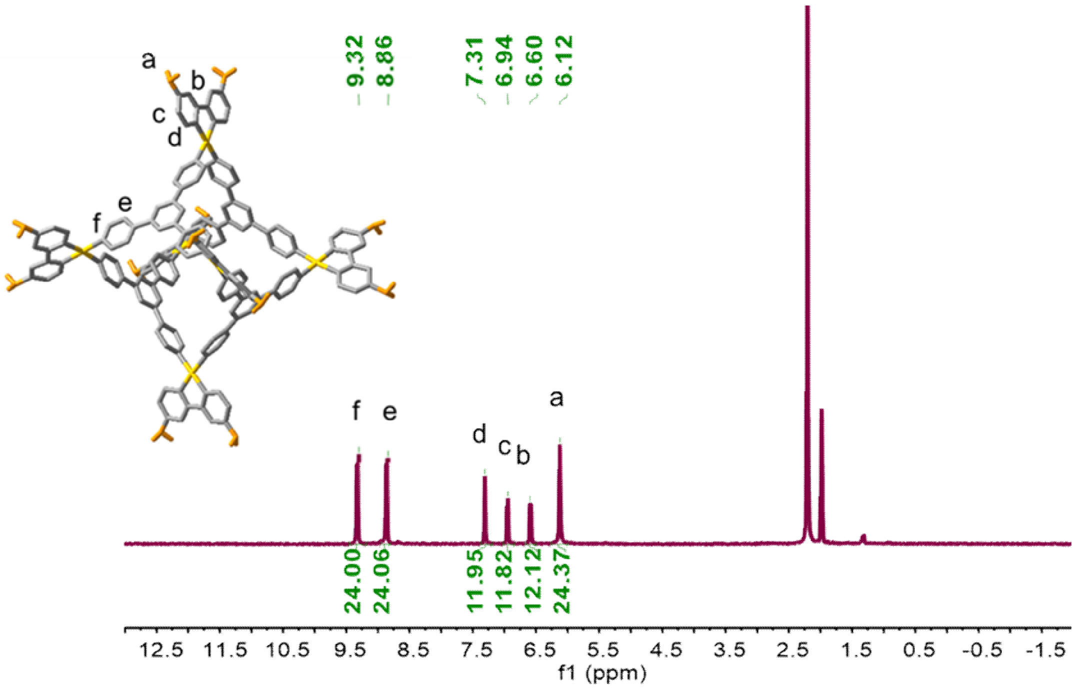

Supplementary Figure 3. ^{1}H NMR spectrum (400 MHz, 300 K) of **PCC-5** (in CD_3CN).

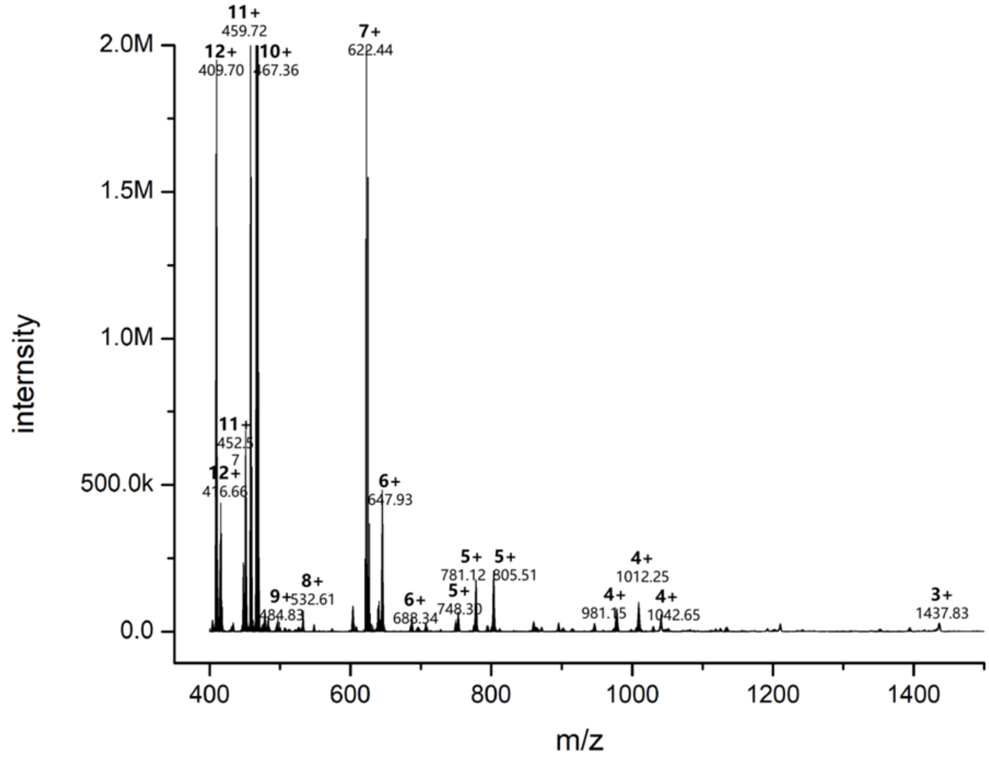

Supplementary Figure 4. ESI-MS spectrum of **PCC-5**.

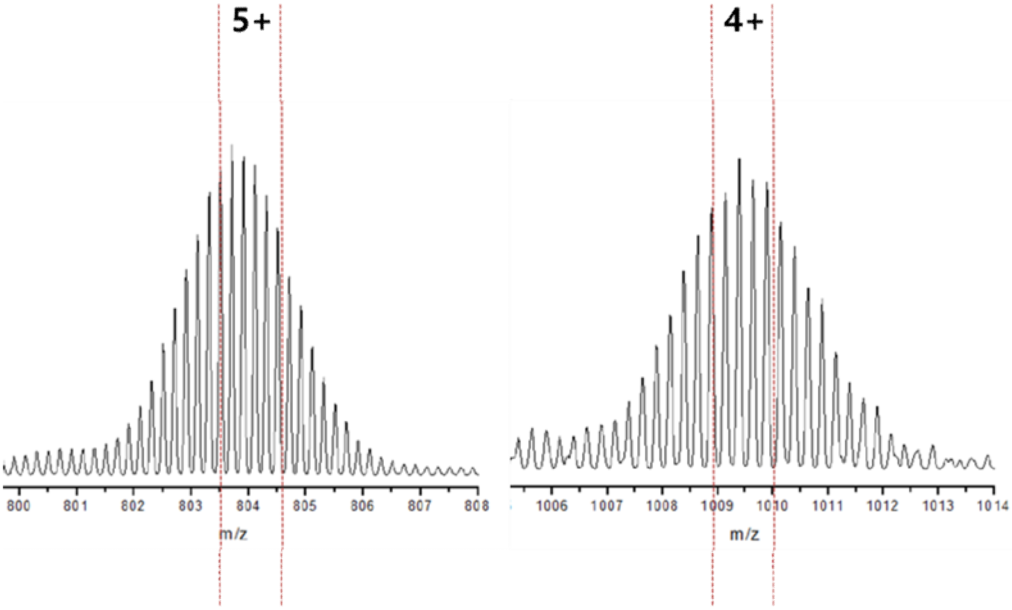

Supplementary Figure 5. Experimental isotopic distributions of ionic peaks belonging to 5+ and 4+ of **PCC-5**.

Supplementary Table 2. Experimental isotopic distributions of **PCC-5**.

Formula	Charge *z*	Calculated *m/z*	Experimental *m/z*
(M-0 PF_6^-) + 4MeCN	12+	410.06	409.70
(M-0 PF_6^-) + 6 MeCN	12+	416.90	416.66
(M-1 PF_6^-) + 9 MeCN	11+	452.82	452.57
(M-1 PF_6^-) + 11 MeCN	11+	460.28	459.72
(M-2 PF_6^-) + 5 MeCN	10+	467.19	467.36
(M-3 PF_6^-) + 1 MeCN	9+	484.74	484.83
(M-4 PF_6^-) + 2 MeCN	8+	532.35	532.61
(M-5 PF_6^-) + 8 MeCN	7+	622.87	622.44
(M-6 PF_6^-) + 0 MeCN	6+	647.80	647.93
(M-6 PF_6^-) + 6 MeCN	6+	688.05	688.34
(M-7 PF_6^-) + 0 MeCN	5+	748.36	748.30
(M-7 PF_6^-) + 4 MeCN	5+	781.02	781.12
(M-7 PF_6^-) + 6 MeCN	5+	805.83	805.51
(M-8 PF_6^-) + 8 MeCN	4+	981.30	981.15
(M-8 PF_6^-) + 11 MeCN	4+	1012.08	1012.25
(M-8 PF_6^-) + 11 MeCN	4+	1042.86	1042.65
(M-9 PF_6^-) + 21 MeCN	3+	1437.98	1437.83

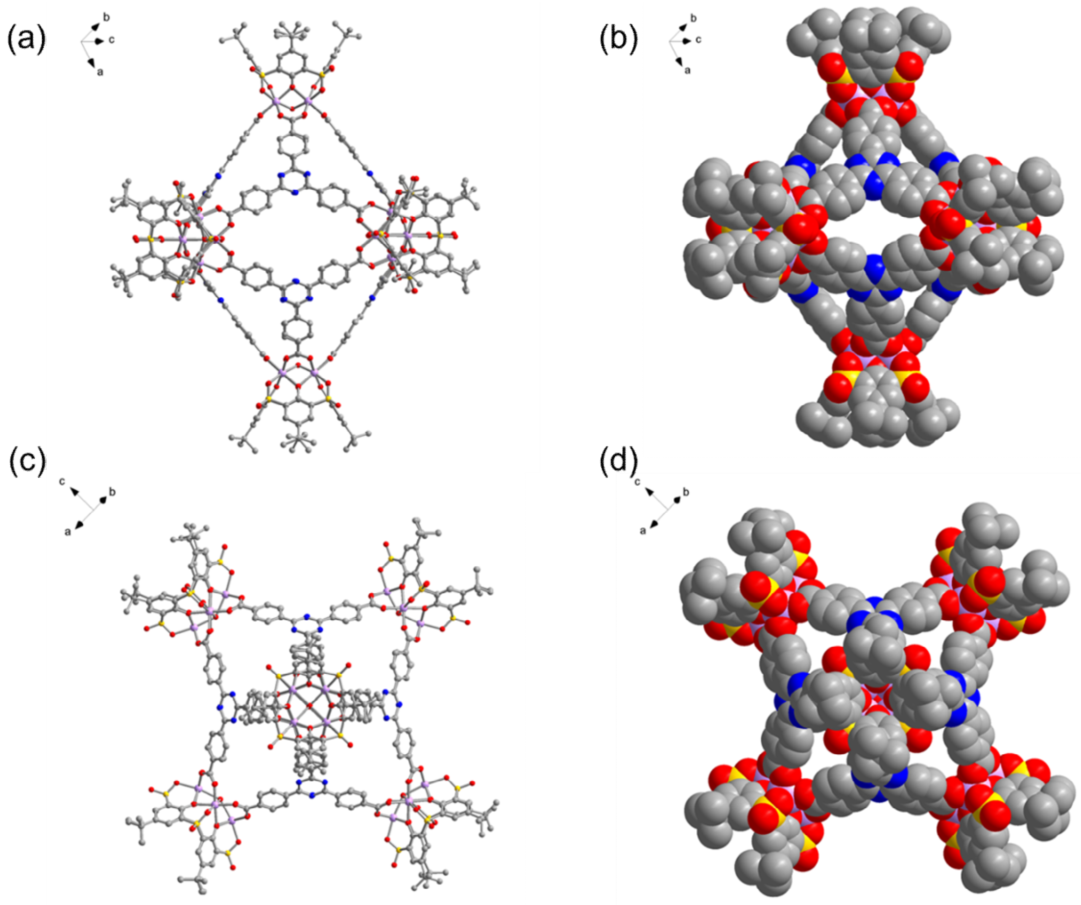

Supplementary Figure 6. Single crystal X-ray structure of **PCC-2b**. (a) (c) Ball-and-stick model. (b) (d) Space-filling Model. (Red: oxygen atom; Gray: carbon atoms; Blue: nitrogen atoms; Yellow: sulfur atoms; Purple: cobalt atom; Hydrogen atoms are omitted for clarity).

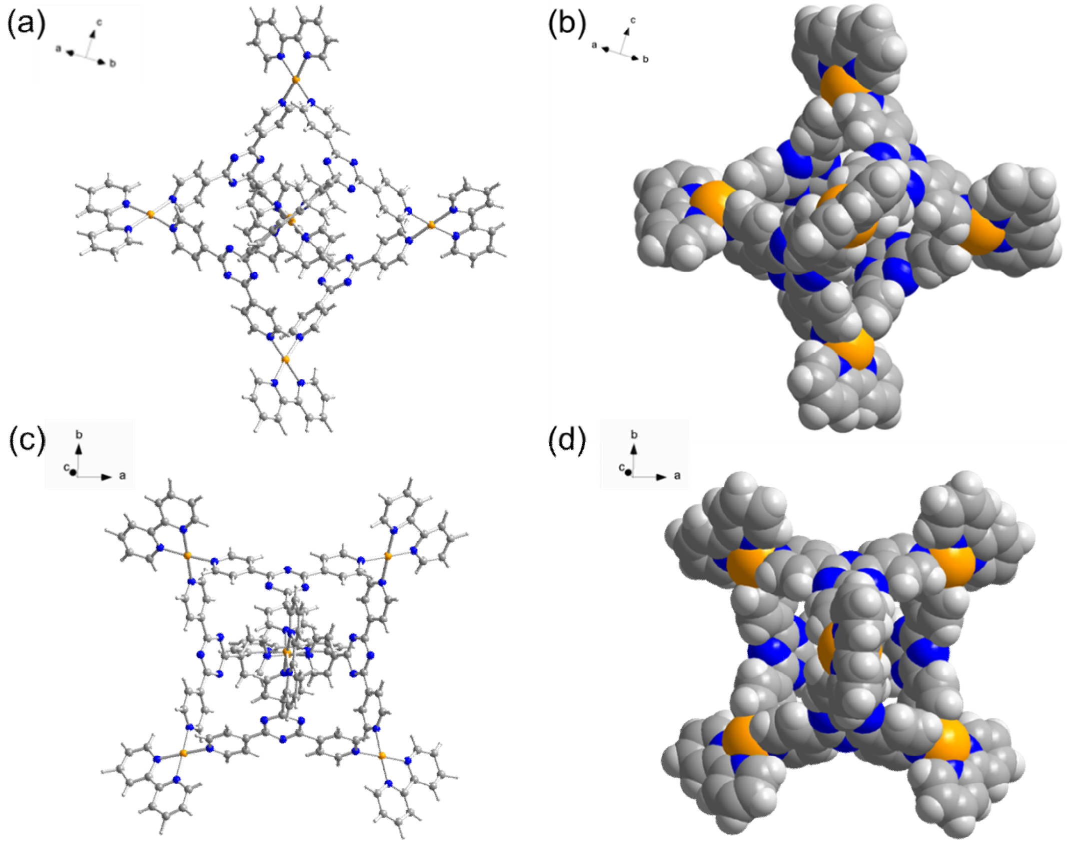

Supplementary Figure 7. Single crystal X-ray structure of **PCC-3**. (a) (c) Ball-and-stick model. (b) (d) Space-filling Model. (Orange: palladium atom; Gray: carbon atoms; Blue: nitrogen atoms; White: hydrogen atoms).

Section 3. Characterizations of PCN-222@PCCs

(a)

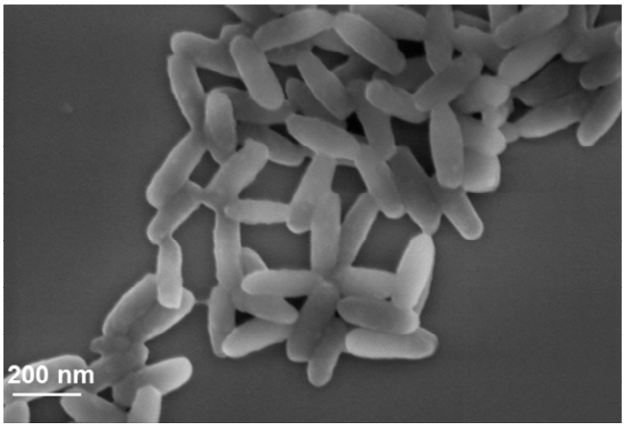

(b)

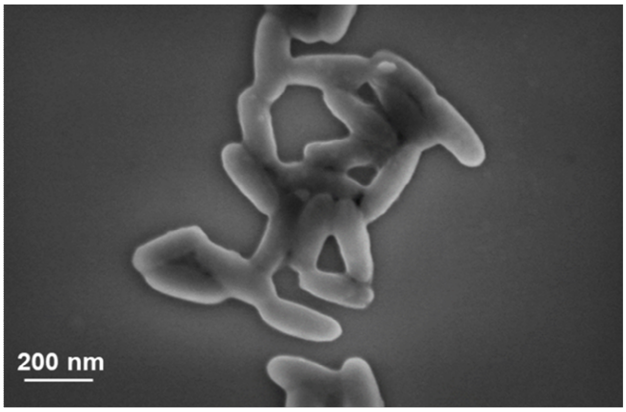

(c)

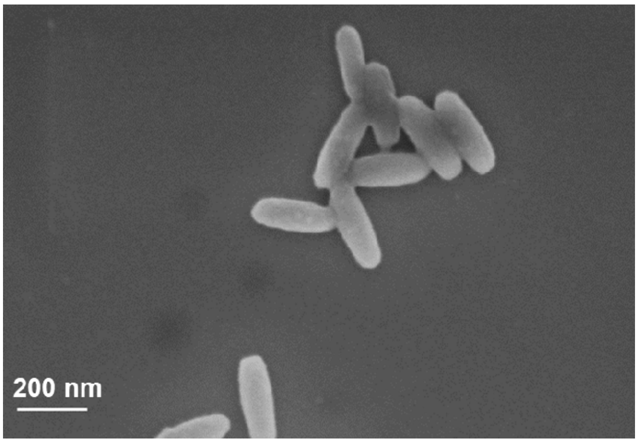

Supplementary Figure 8. SEM images of **PCN-222** (a), **PCN-222@PCC-4** (b), and **PCN-222@PCC-5** (c). Scale bar = 200 nm.

(a)

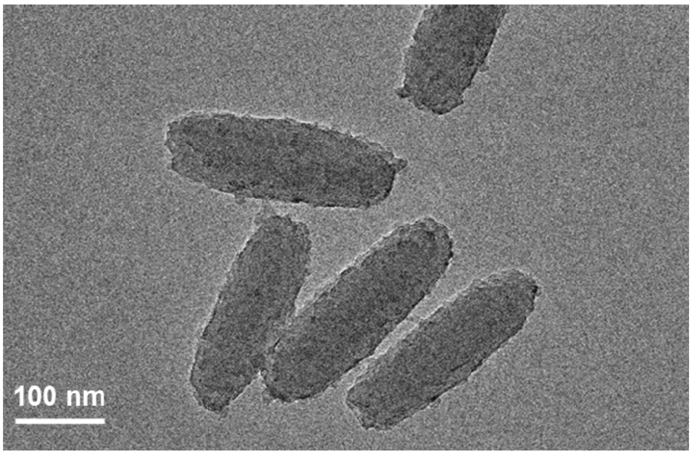

(b)

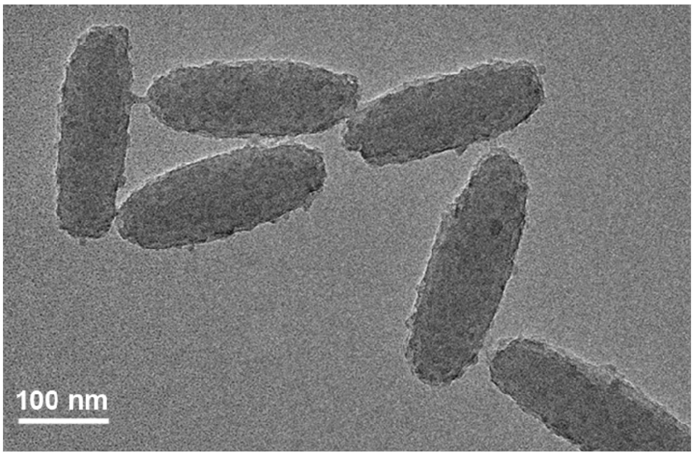

(c)

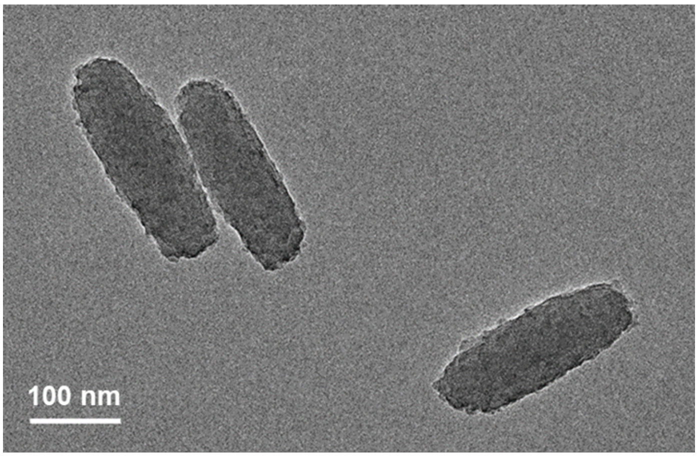

Supplementary Figure 9. TEM of **PCN-222** (a), **PCN-222@PCC-4** (b) and **PCN-222@PCC-5** (c). Scale bar = 100 nm.

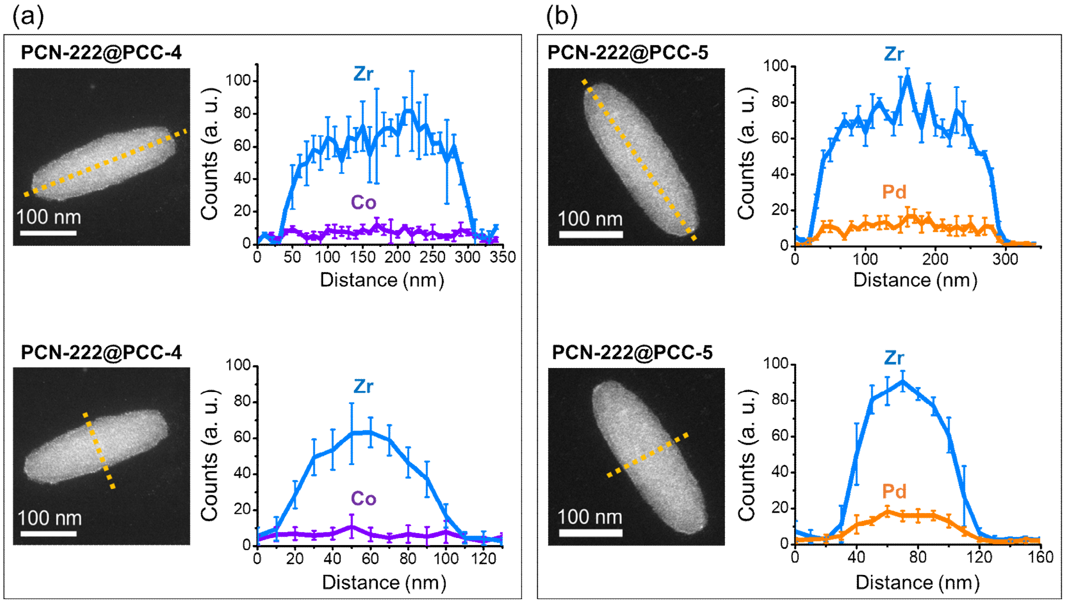

Supplementary Figure 10. STEM images and linear scanning analysis with normalized intensities of (a) **PCN-222@PCC-4**, (b) **PCN-222@PCN-5**. Data are presented as the mean ± SD.

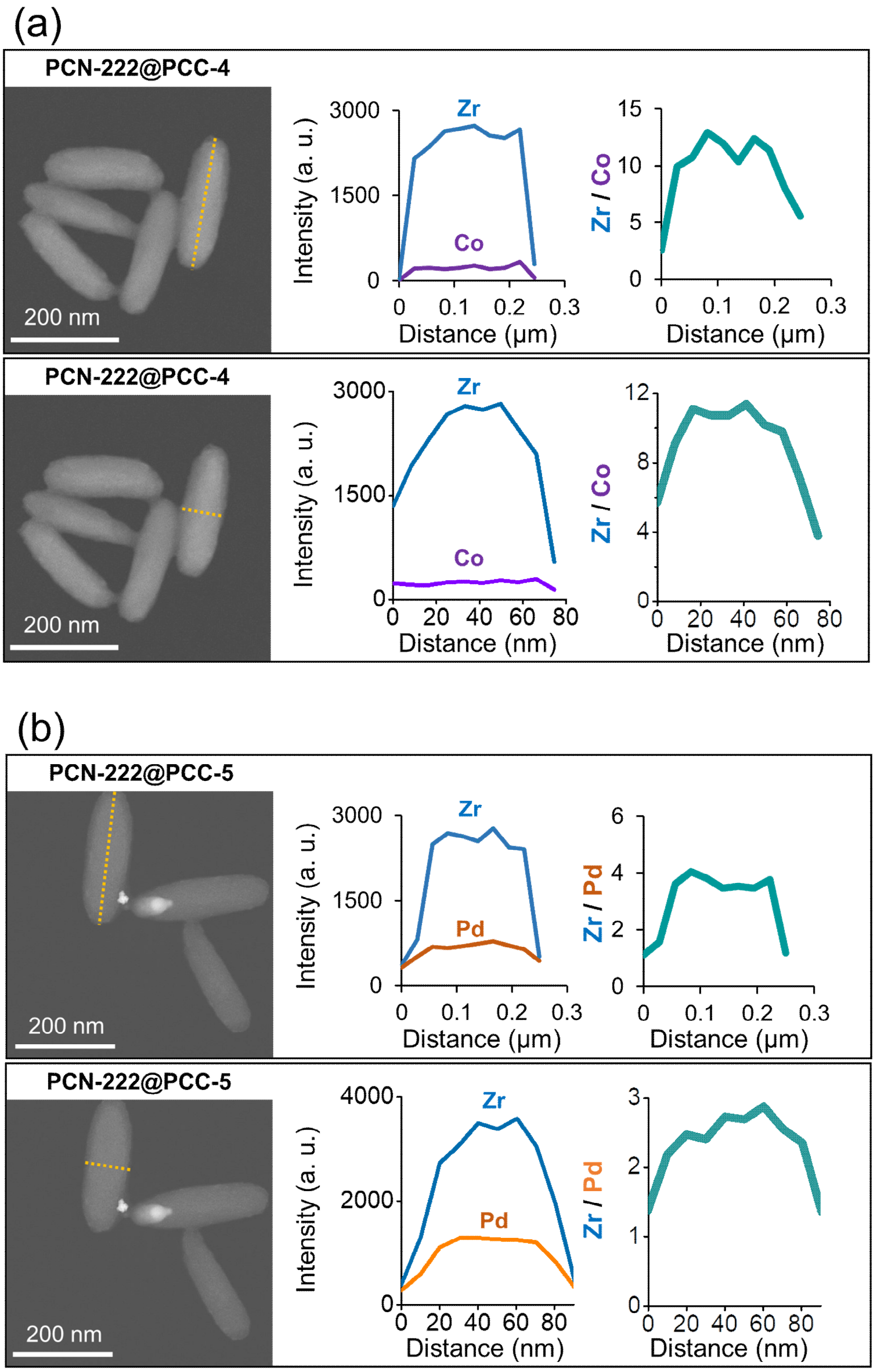

Supplementary Figure 11. Examinations of the (a) Zr/Co and (b) Zr/Pd element ratios on PCN-222@PCCs samples. The curves demonstrated a lower Zr/PCCs ratio at the thin edge and a higher Zr/PCC ratio at the thick center.

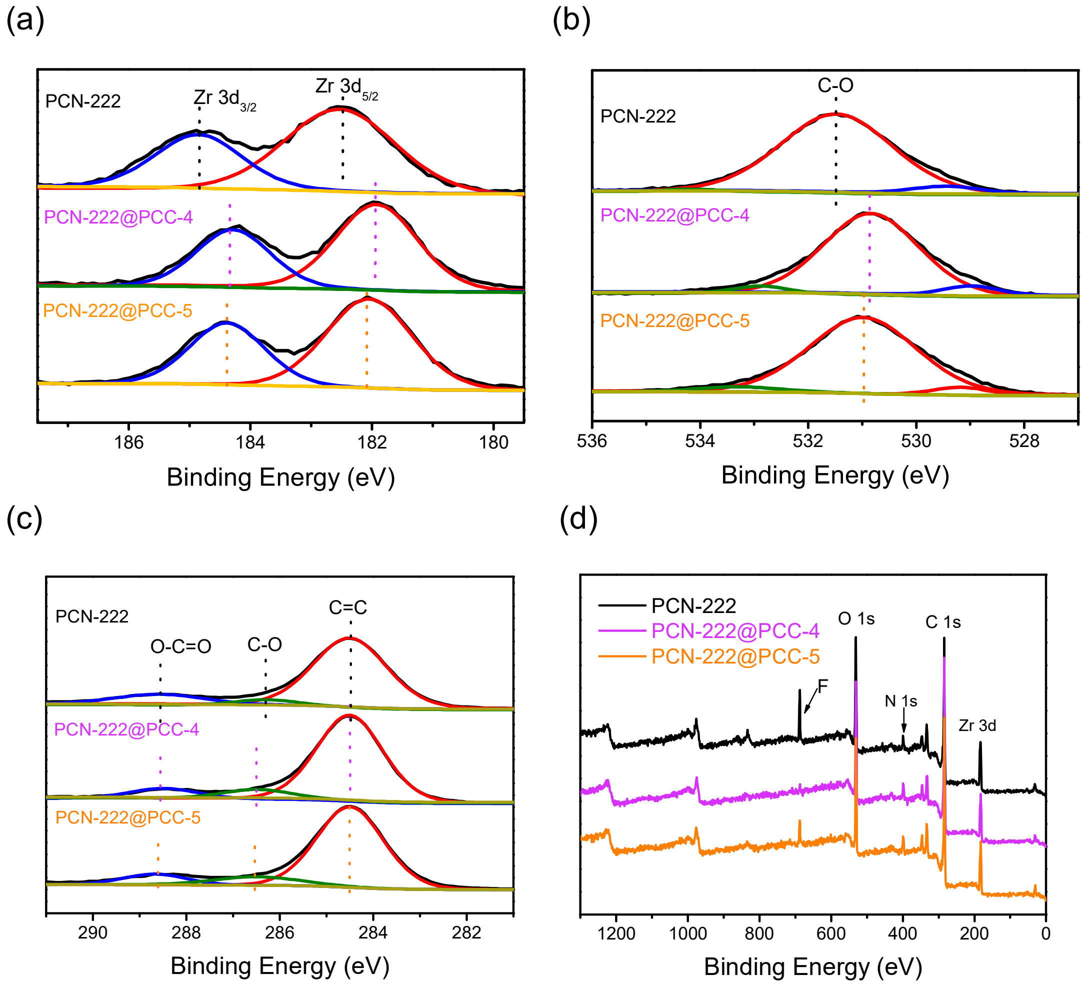

Supplementary Figure 12. *XPS* spectra of **PCN-222**, **PCN-222@PCC-4**, and **PCN-222@PCC-5**. (a) Zr 3*d*, (b) O 1*s*, (c) C 1*s*, and (d) survey spectra.

(a)

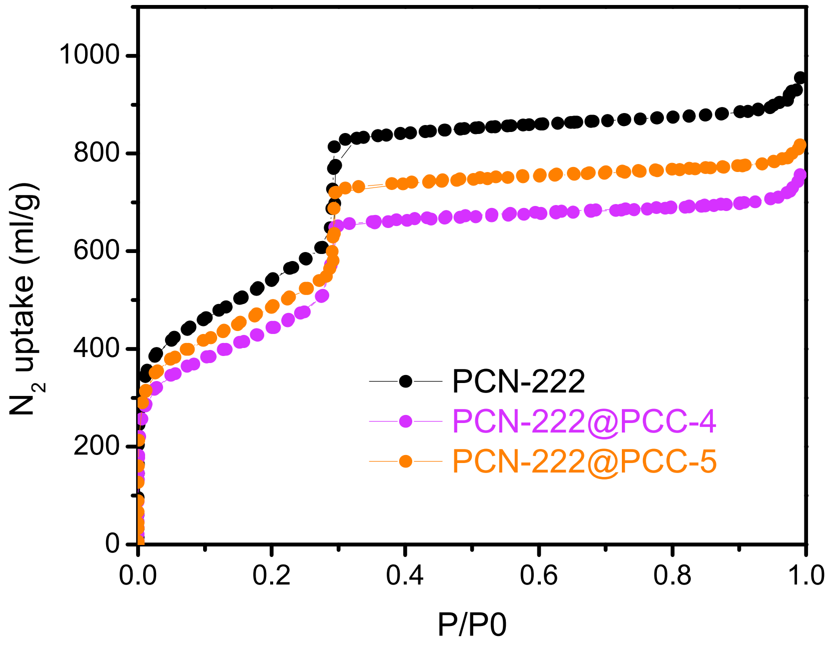

(b)

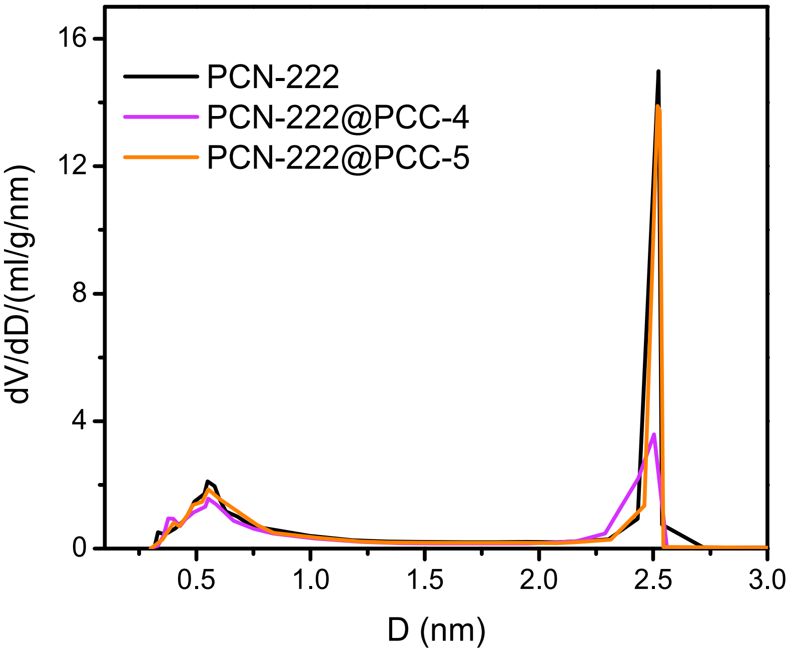

Supplementary Figure 13. The Nitrogen adsorption isotherms (a) and pore size distributions (b) of **PCN-222**, **PCN-222@PCC-**4, and **PCN-222@PCC-5**.

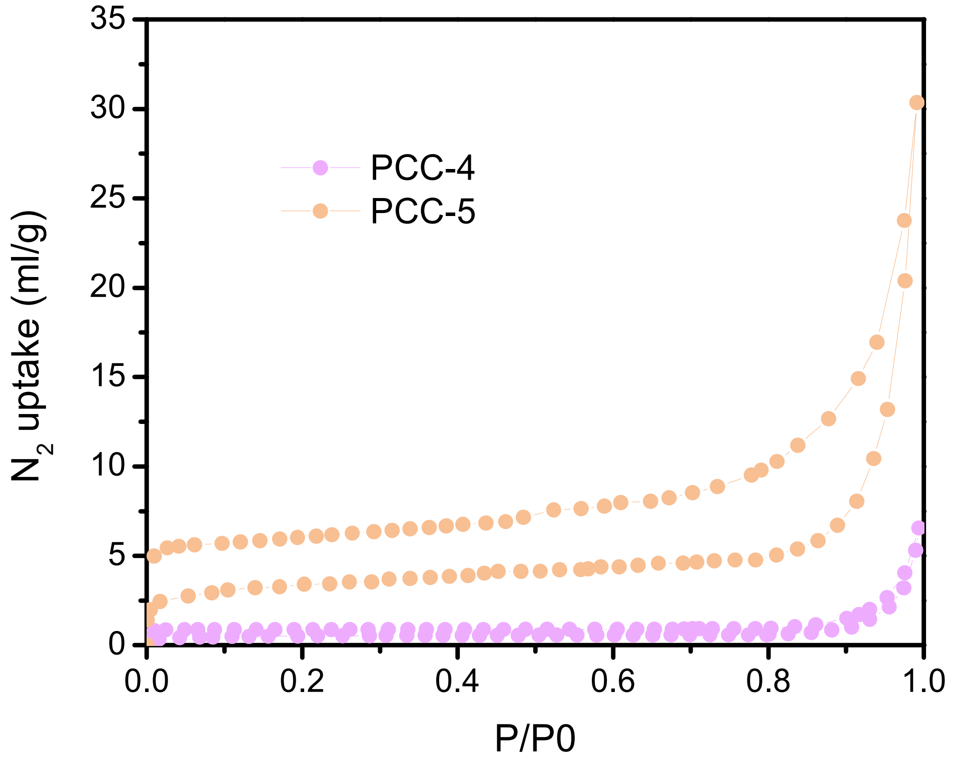

Supplementary Figure 14. The Nitrogen adsorption and desorption of **PCC-4** and **PCC-5**.

Supplementary Table 3. ICP-OES data of **PCN-222@PCCs**

	V_0 (mL)	C_{Zr} (mg/L)	C_{Co} (mg/L)	C_{Pd} (mg/L)	PCC/PCN-222@PCC (wt%)
PCN-222@PCC-4	10	24.24	0.76	/	5.40
PCN-222 mixed with PCC-4	10	12.33	0.10	/	1.46
PCN-222@PCC-2b	10	11.12	0.07	/	1.02
PCN-222@PCC-5	10	21.97	/	0.26	1.98
PCN-222 mixed with PCC-5	10	13.67	/	0.13	1.60
PCN-222@PCC-3	10	12.93	/	0.08	0.79

The molecular formula of PCN-222 is $Zr_6O_{32}N_8C_{96}H_{53}$, and the relative molecular weight is 2377.83, in which Zr accounts for 23.02% of the relative molecular weight.

The molecular formula for the PCC-4 is $C_{336}H_{144}S_{48}N_{24}O_{198}Co_{24}Na_{24}$, the relative molecular weight is 11190.05, in which Co accounts for 12.64% of the relative molecular weight.

The molecular formula for the PCC-5 is $Pd_6N_{48}C_{132}H_{108}P_{12}F_{72}$, the relative molecular weight is 4744.68, in which Pd accounts for 13.46% of the relative molecular weight.

The molecular formula for the PCC-2b is $C_{432}H_{360}S_{24}N_{24}O_{126}Co_{24}$, the relative molecular weight is 10087.52, in which Co accounts for 14.02% of the relative molecular weight.

The molecular formula for the PCC-3 is $Pd_6C_{132}H_{96}N_{48}O_{36}$, the relative molecular weight is 3569.00, in which Pd accounts for 17.89% of the relative molecular weight.

In PCN-222@PCC-4, the concentration of Co is 0.76 mg/L, Zr is 24.24 mg/L, and the constant volume is 10 ml.

M_{Co}=0.76×10×10^{-3}=0.0076 mg, M_{PCC-4}=0.0076/0.1264≈0.06013 mg;

M_{Zr}=24.24×10×10^{-3}=0.2424 mg, $M_{PCN-222}$=0.2424/0.2302≈1.05300 mg;

$M_{PCN-222@PCC-4}$=0.06013+1.05300=1.11313 mg;

The mass proportion of PCC-4 in PCN-222@PCC-4 is 0.06013/1.11313≈ 5.40%.

In PCN-222 mixed with PCC-4, the concentration of Co is 0.10 mg/L, Zr is 12.33 mg/L, and the constant volume is 10 ml.

M_{Co}=0.10×10×10^{-3}=0.001 mg, M_{PCC-4}=0.001/0.1264≈0.00791 mg

M_{Zr}=12.33×10×10^{-3}=0.1233 mg, $M_{PCN-222}$=0.1233/0.2302≈0.53562 mg

$M_{\text{PCN-222 mix with PCC-4}}$=0.53562+0.00791=0.54353 mg

The mass proportion of PCC-4 in the PCN-222 mix with PCC-4 is 0.00791/0.54353≈1.46%.

In PCN-222@PCC-2b, the concentration of Co is 0.07 mg/L, Zr is 11.12 mg/L, and the constant volume is 10 ml.

M_{Co}=0.07×10×10^{-3}=0.0007 mg, M_{PCC-4}=0.0007/0.1402≈0.00499 mg,

M_{Zr}=11.12×10×10^{-3}=0.1112mg, $M_{PCN-222}$=0.1112/0.2302≈0.48306 mg。

$M_{PCN-222@PCC-2b}$=0.00554 mg+0.48306 mg=0.48805 mg

The mass proportion of PCC-2b in PCN-222@PCC-2b is 0.00499/0.48805≈1.02 %.

In PCN-222@PCC-5, the concentration of Pd is 0.26 mg/L, Zr is 21.97 mg/L, and the constant volume is 10 ml.

M_{Pd}=0.26×10×10^{-3}=0.0026 mg, M_{PCC-5}=0.0026/0.1346≈0.01932mg

M_{Zr}=21.97×10×10^{-3}=0.2197 mg, $M_{PCN-222}$=0.2197/0.2302≈0.95439 mg

$M_{PCN-222@PCC-5}$=0.01932+0.95439=0.97371

The mass proportion of PCC-5 in PCN-222@PCC-5 is 0.01932/0.97371≈1.98%

In PCN-222 mixed with PCC-5, the concentration of Pd is 0.13 mg/L, Zr is 13.67 mg/L, and the constant volume is 10 ml.

M_{Pd}=0.13×10×10^{-3}=0.0013 mg, M_{PCC-5}=0.0013/0.1346≈0. 00966 mg

M_{Zr}=13.67×10×10^{-3}=0.1367 mg, $M_{PCN-222}$=0.1367/0.2302≈0.59383 mg

$M_{PCN-222\ mix\ with\ PCC-5}$=0. 00966+0.59383=0.60349

The mass proportion of PCC-5 in PCN-222 mix with PCC-5 is 0. 00966/0.60349≈1.60 %

In PCN-222@PCC-3, the concentration of Pd is 0.08 mg/L, Zr is 12.93 mg/L, and the constant volume is 10 ml.

M_{Pd}=0.08×10×10^{-3}=0.0008 mg, M_{PCC-3}=0.0008/0.1789≈0.00447 mg

M_{Zr}=12.93×10×10^{-3}=0.1293 mg, $M_{PCN-222}$=0.1293/0.2302≈0.56167 mg

$M_{PCN-222@PCC-3}$=0.00447+0.56167=0.56614

The mass proportion of PCC-3 in PCN-222@PCC-3 is 0.00447/0.56614≈0.79 %

We conducted an evaluation of the chemical and thermal stability of the prepared **MOF@PCC** composites. We investigated the potential leaching of the cage by soaking **PCN-222@PCCs** in organic solvents that dissolve the cage, such as DMF and MeCN, for 24 hours. The supernatant was collected and analyzed using a UV-vis spectrometer (Supplementary Fig. 15). Interestingly, no characteristic peaks from the cage were detected, indicating that the coordination interactions between the cage and MOF are robust enough to prevent any potential leaching.

The stability of PCC alone was studied at 25°C, 40°C, 60°C, 80°C, and 100°C. It was found that PCC-4 remained stable under 25-80°C, while gradually decomposing at 100°C by showing a distinct UV-Vis spectrum (Supplementary Fig. 16a). In contrast, PCC-5 remained stable under 25-100°C by showing a consistent UV-vis peak (Supplementary Fig. 16b).

Then, the stability of PCN-222@PCCs and MIL-101@PCCs was studied at 25°C, 40°C, 60°C, 80°C, and 100°C by soaking the materials in the solvent and characterizing the supernatant by UV-Vis. For PCN-222@PCC-4 under 25-80°C, no characteristic peak belonging to PCC-4 was found, indicating the absence of PCC leaching or decomposing (Supplementary Fig. 17a). When the temperature was elevated to 100°C, little amount of decomposed PCC-4 was found in the supernatant (Supplementary Fig. 17b), which well-matched the results discussed above.

(a)

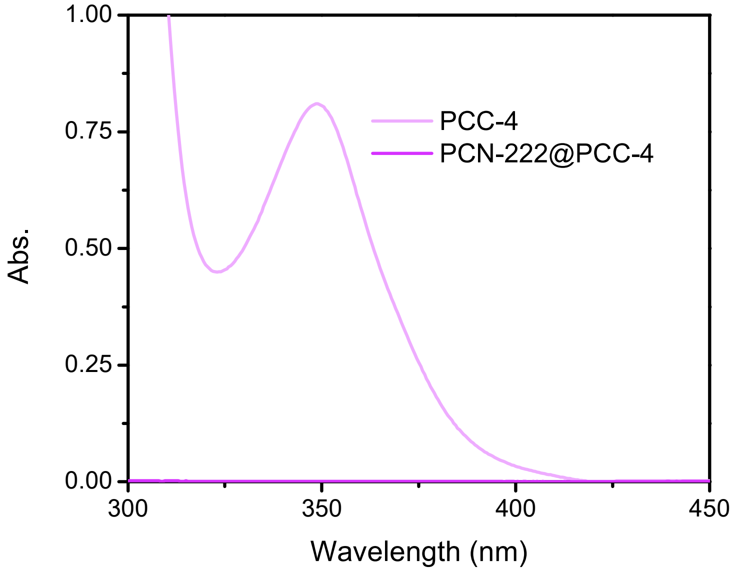

(b)

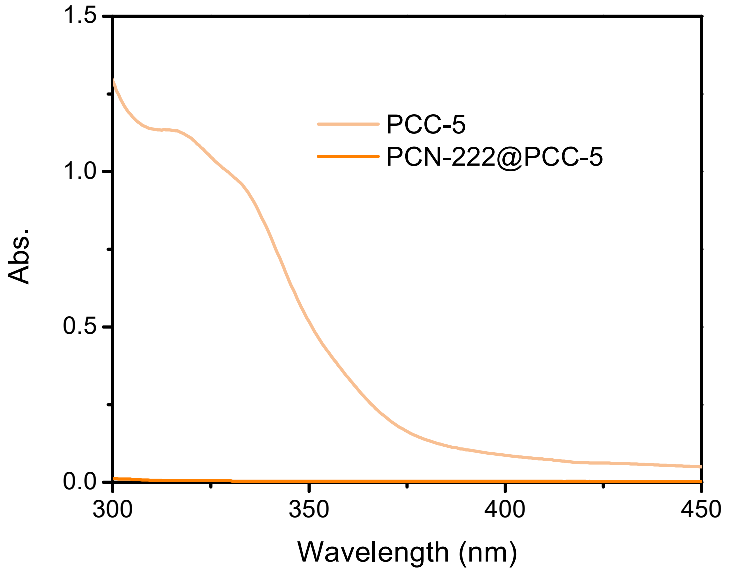

Supplementary Figure 15. **PCN-222@PCC-4** was immersed in DMF and the supernatant was detected after 24 h (a). **PCN-222@PCC-5** was immersed in CH_3CN and the supernatant was detected after 24 h (b).

(a)

(b)

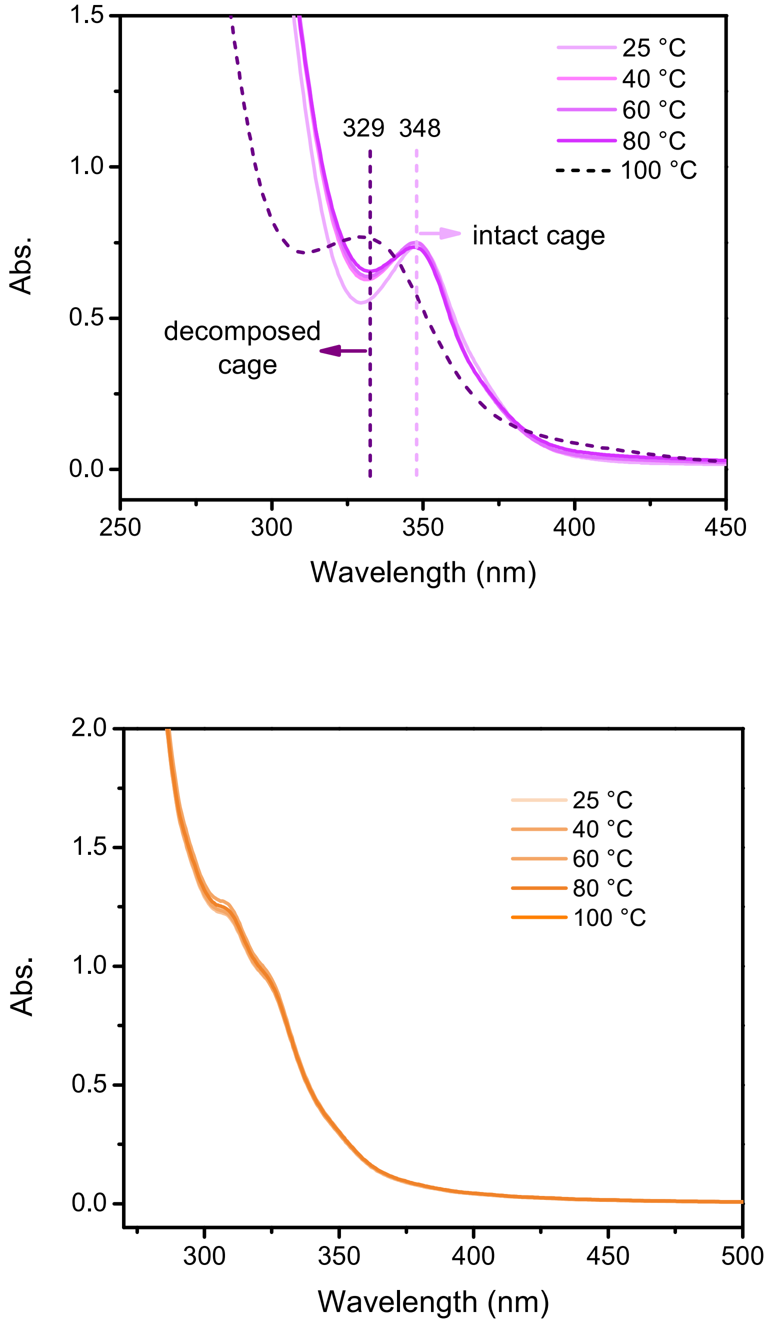

Supplementary Figure 16. The stability of **PCC-4** (a) and **PCC-5** (b) at different temperatures.

(a)

(b)

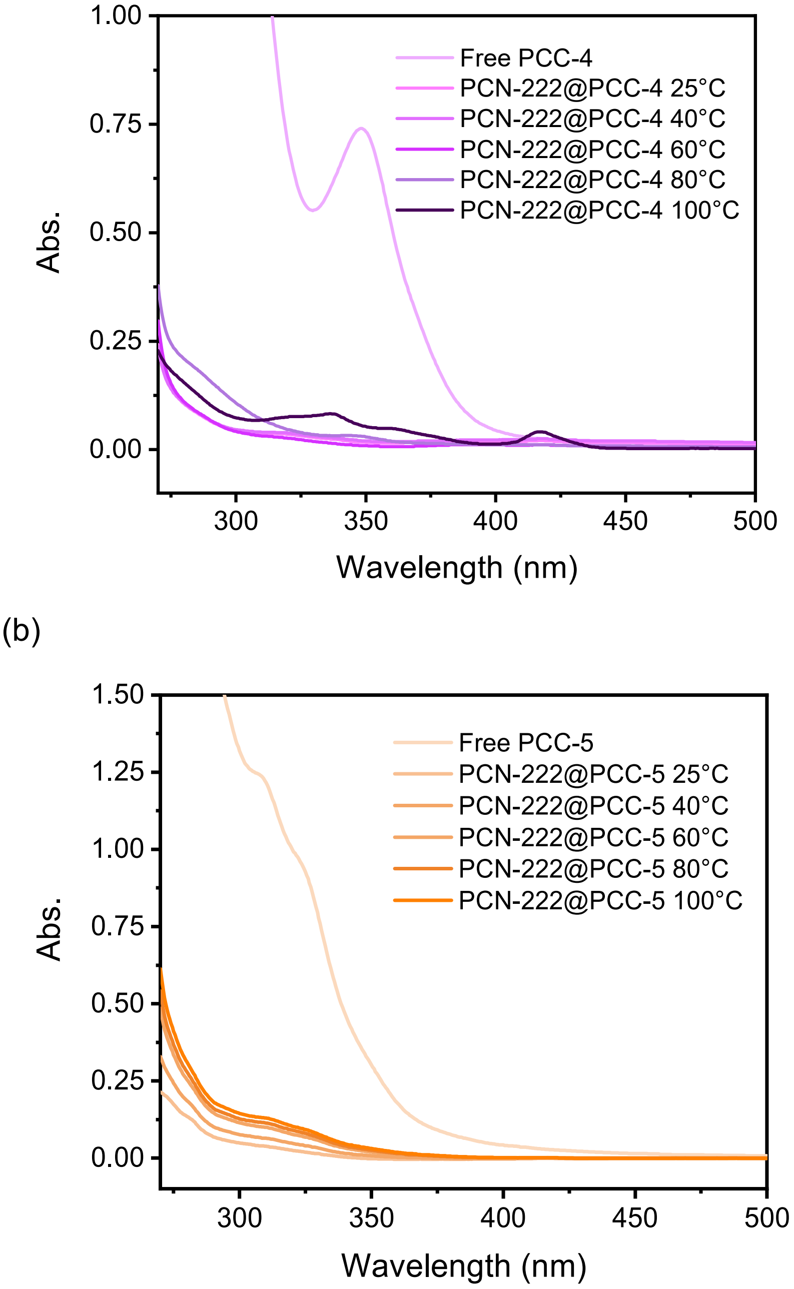

Supplementary Figure 17. The stability of **PCN-222@PCC-4** (a) and **PCN-222@PCC-5** (b) at different temperatures.

(a)

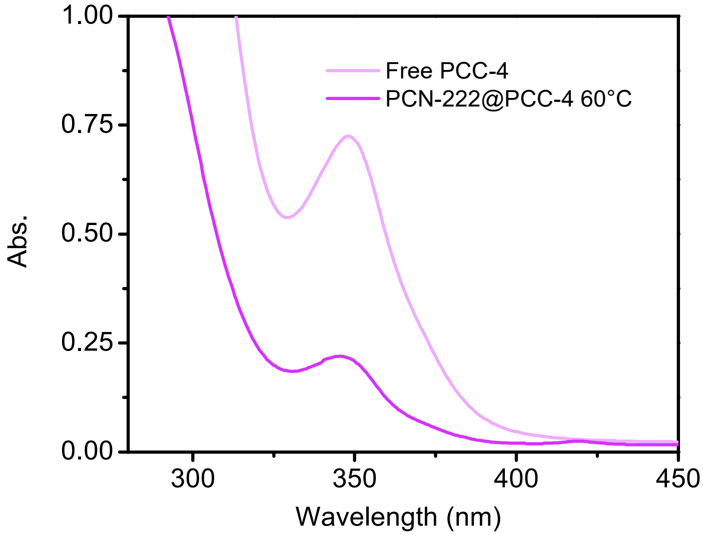

(b)

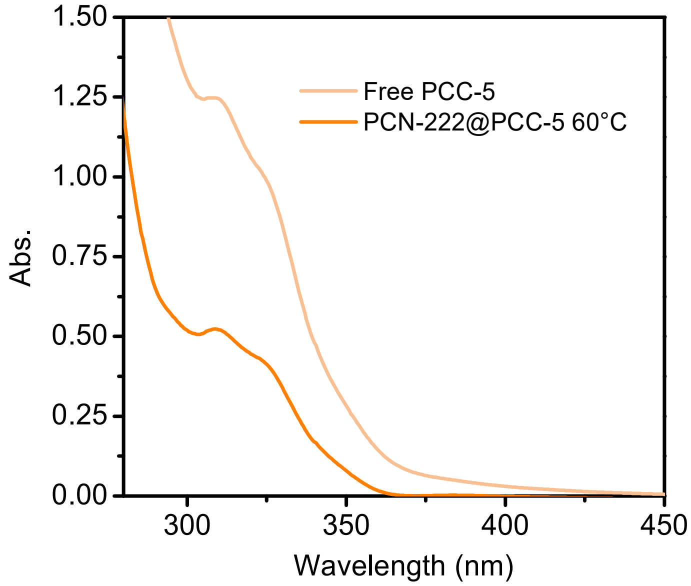

Supplementary Figure 18. UV-vis spectra of treated samples. **PCC-4** (a) and **PCC-5** (b). We utilized UV-vis spectroscopy to investigate the stability of **PCN-222@PCCs** in solution. To evaluate the stability of the PCCs on the surface of **PCN-222@PCC**, we attempted to remove the PCCs by repeatedly sonicating the composite and analyzing the supernatant solution by UV-vis. As demonstrated in Supplementary Fig. 18, the characteristic peak of the PCCs did not show any changes, suggesting that the PCCs remained stable on the surface of **PCN-222@PCC** and were not released into the solution.

In order to examine the role of secondary coordination sites on PCCs, control groups were synthesized consisting of **PCC-2b** and **PCC-3**, which lack secondary coordination sites. These PCCs were introduced to **PCN-222** via the same modification process as mentioned earlier to prepare **PCN-222@PCC-2b** and **PCN-222@PCC-3** composites. The obtained composites were characterized using SEM, PXRD, zeta potential, and N_2 adsorption isotherms (Supplementary Figs. 19-22). As expected, the non-covalent modification of PCCs did not alter the crystal morphology, particle size, or crystallinity of the host MOF. However, the N_2 uptake capacity was significantly decreased by approximately 36% after modification with **PCC-2b** or **PCC-3** (Supplementary Fig. 22), in contrast to the groups with secondary coordination sites (Figure 2i). This is possibly because the PCCs without secondary coordination sites are smaller in size and can fill the pores, obstructing the accessible voids of mesoporous MOFs. Conversely, PCCs with secondary coordination sites predominantly modify the surface of MOFs without diminishing porosity. The interactions between cage and MOF for **PCC-2b** and **PCC-3** were further evaluated using ICP-OES, where the number of loaded cages on MOF was determined after collecting the particles and removing the supernatant. As anticipated, the cage loading amount on MOF was only approximately 10.2 mg/g for **PCC-2b** and 7.9 mg/g for **PCC-3**, which were significantly lower than their counterparts with secondary coordination sites (Supplementary Table. 3 and Supplementary Fig. 23), further validating the significance of coordination interactions between cage and MOF.

(a)

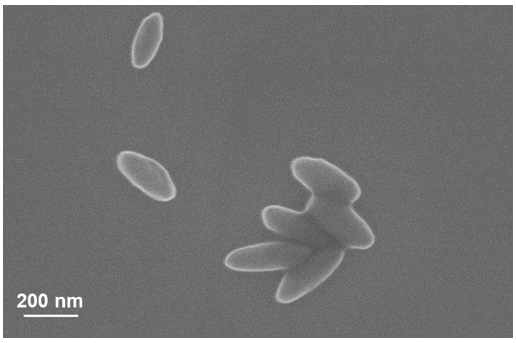

(b)

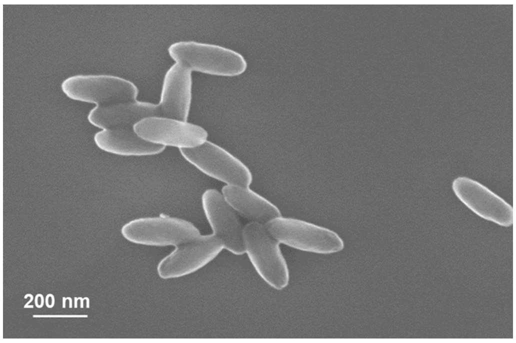

Supplementary Figure 19. SEM of **PCN-222@PCC-2b** (a) and **PCN-222@PCC-3** (b). Scale bar = 200 nm.

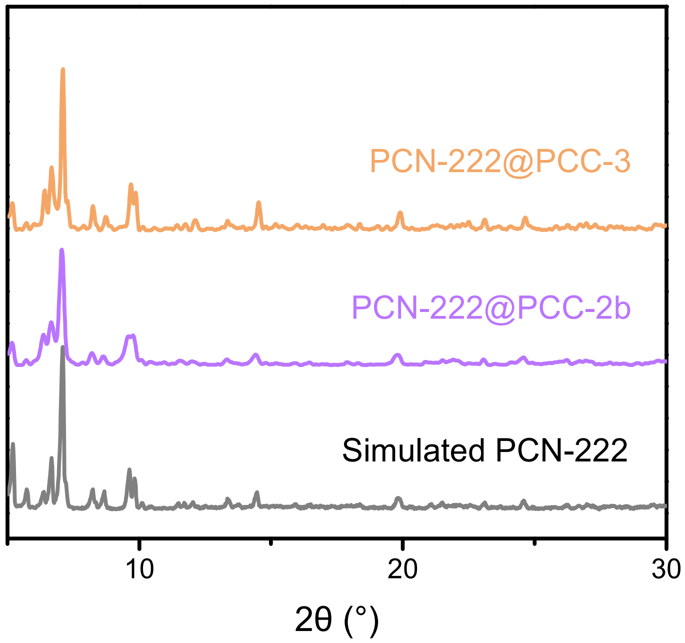

Supplementary Figure 20. PXRD of **PCN-222**, **PCN-222@PCC-2b** and **PCN-222@PCC-3**.

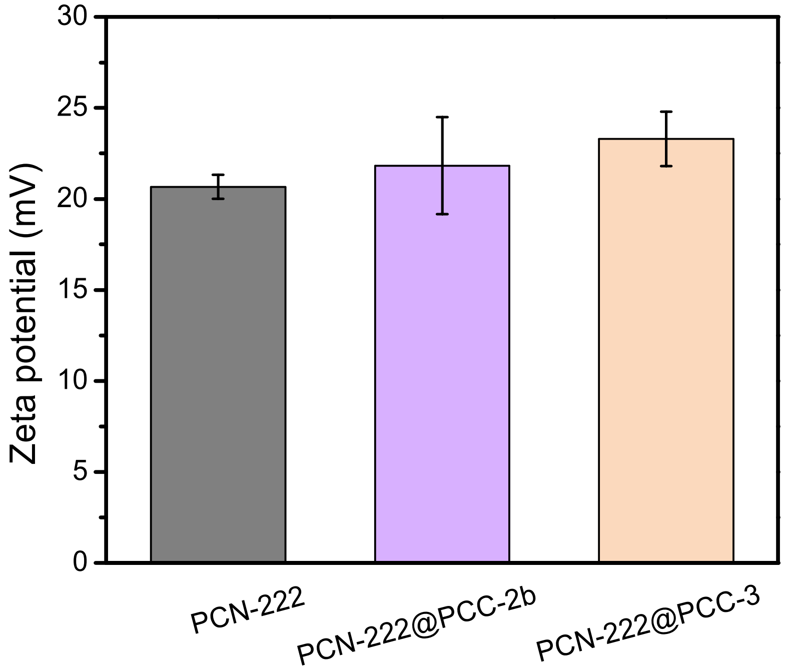

Supplementary Figure 21. Zeta potential of **PCN-222**, **PCN-222@PCC-2b** and **PCN-222@PCC-3**. Data are presented as the mean ± SD.

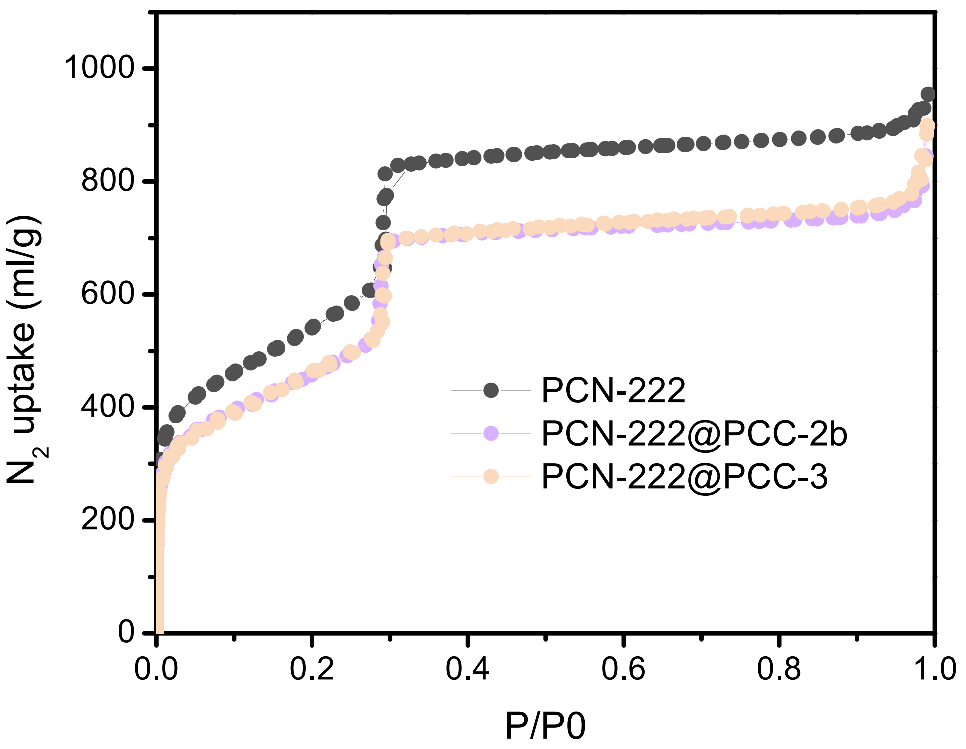

Supplementary Figure 22. Nitrogen adsorption and desorption of **PCN-222**, **PCN-222@PCC-2b**, and **PCN-222@PCC-3**.

(a)

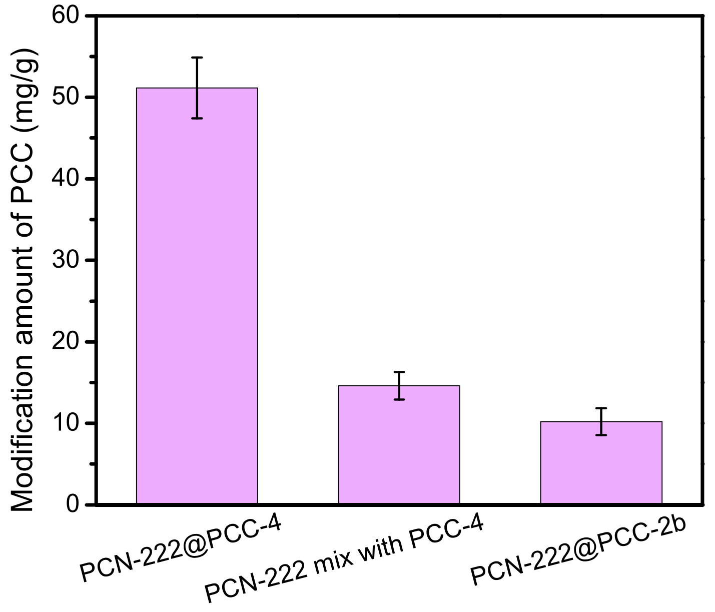

(b)

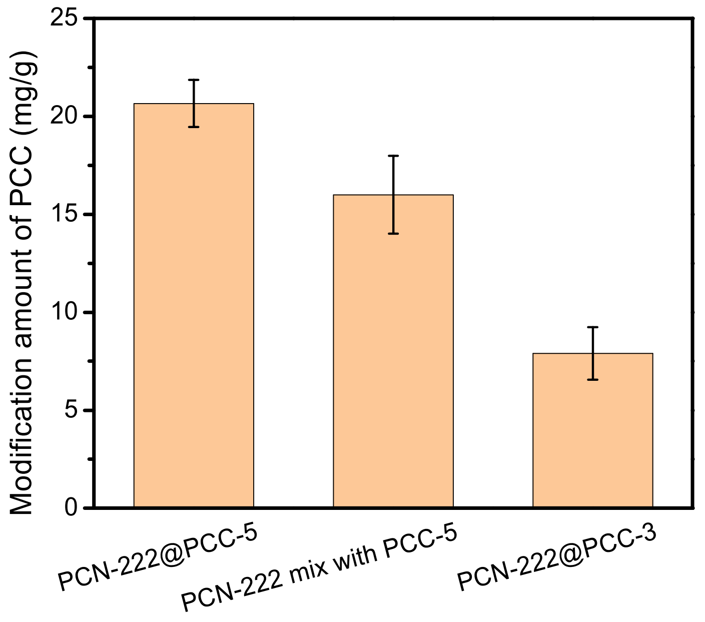

Supplementary Figure 23. The loading amount of **PCCs** in different composites. Data are presented as the mean ± SD.

Section 4. Theoretical Calculations of PCN-222@PCCs

The cell parameters of PCN-222 ($Zr_{18}C_{396}O_{147}N_{32}H_{228}$) are as follows:

a=42.262 Å, α= 90 °

b=42.262 Å, β= 90 °

c=16.951 Å, γ=120 °

V = 26219.6 A^3 = 26.2196 nm^3

Suppose PCN-222 is a cylinder with a height of 200 nm and a bottom diameter of 50 nm.

The base area of PCN-222 is 3.14×25×25= 1962.5 nm^2

The volume of PCN-222 is 392500 nm^3.

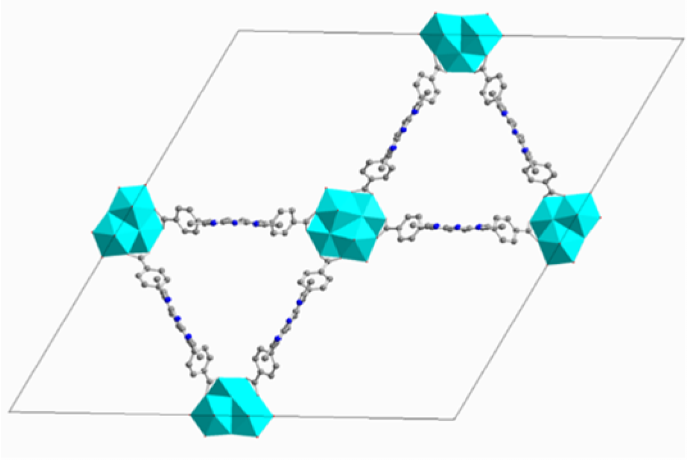

{001} of PCN-222

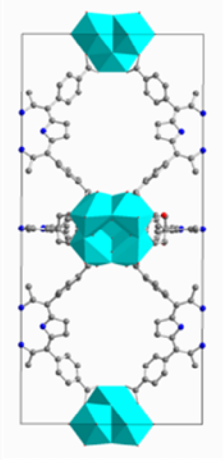

{100} of PCN-222

PCN-222 has a cell volume of 26.22 nm^3 (containing 3 Zr_6 clusters), so PCN-222 nanoparticles contain a total of Zr_6 clusters =392500/26.22×3=44908.5.

The bottom surface of PCN-222 nanoparticles is {001}, and the {001} surface of each single cell contains three equivalent groups of Zr_6 clusters. The side is {100}, and the {001} side of each cell contains an equivalent set of Zr_6 clusters.

The {001} surface area of PCN-222 was 15.73 nm^2.

The {100} area of PCN-222 was 7.16 nm^2.

The surface Zr_6 cluster of PCN-222 has = (1962.5/15.73) ×3×2+31400/7.16=5134.

According to the ICP-OES:

	Zr (mg)	Co (mg)	Pd (mg)
PCN-222@PCC-4	2.4242	0.0764	/
PCN-222@PCC-5	2.1978	/	0.0260

The number of Zr_6 clusters and PCC can be calculated as follows:

	Zr_6	PCC-4	PCC-5
PCN-222@PCC-4	98.80	1.20	/
PCN-222@PCC-5	98.99	/	1.01

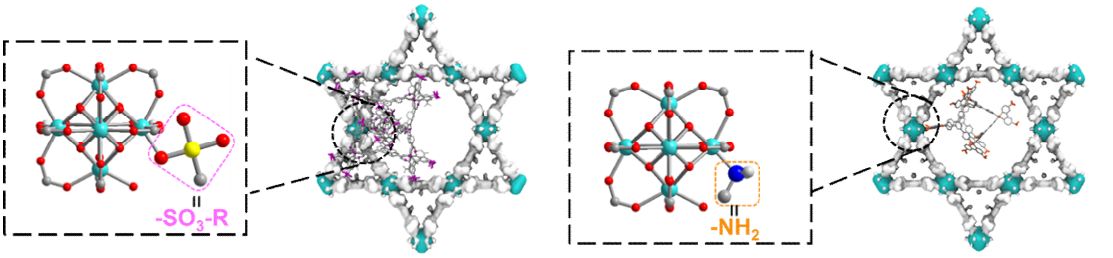

PCN-222@PCC-4 PCN-222@PCC-5

According to the calculation, each PCN-222 nanoparticle can support 538.90 PCC-4 or 453.58 PCC-5.

PCC-4: Surface Zr_6 cluster = 538.9/5134≈10.50%; PCC-5: Surface Zr_6 cluster =453.58/ 5134≈ 8.83%.

In PCN-222@PCC, the amount of PCC modification is about 10% of the number of Zr_6 clusters on the surface, so it can be considered that PCCs are mainly distributed on the surface of PCN-222.

Section 5. Dye adsorptions of PCN-222@PCCs

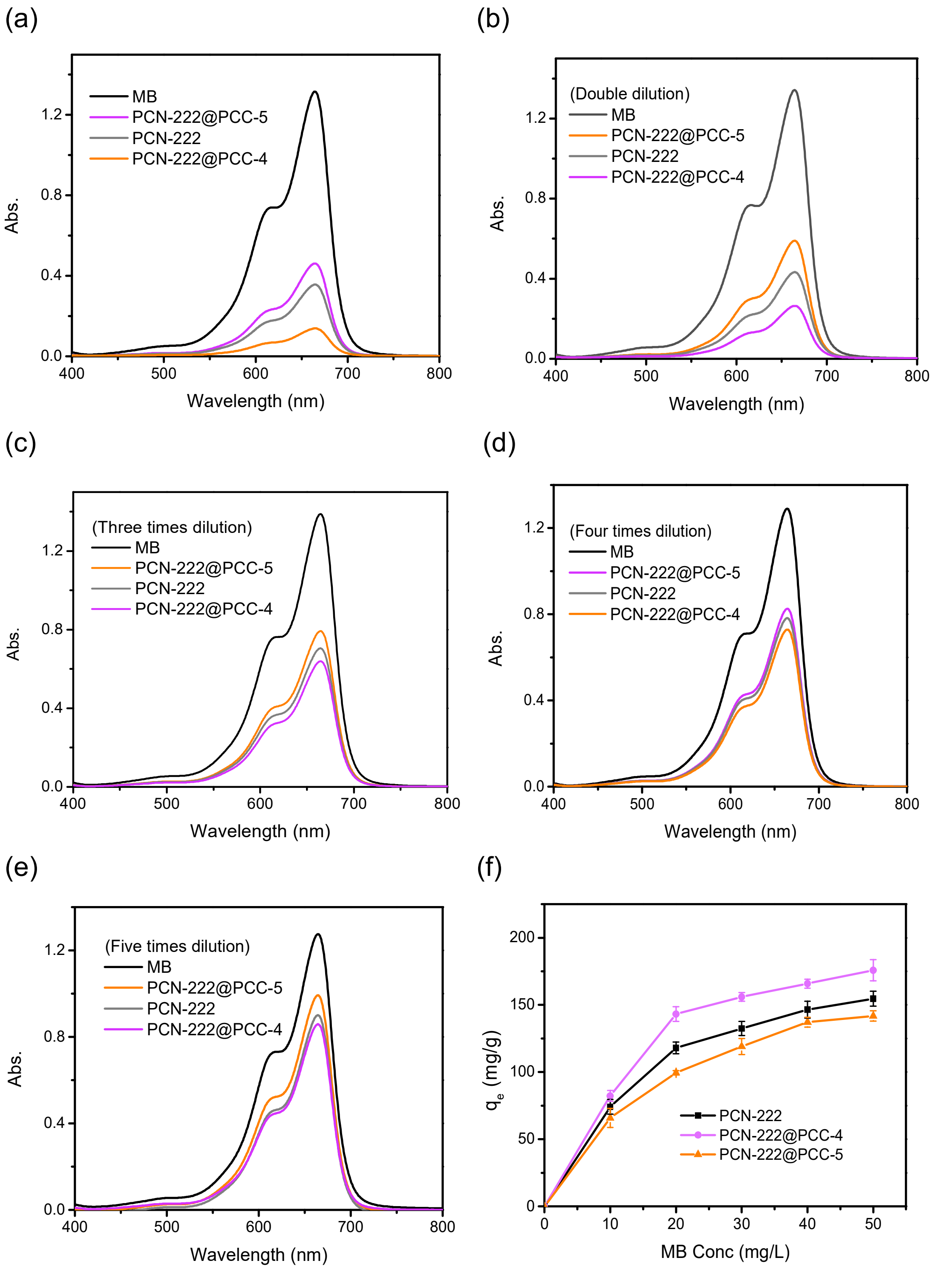

Supplementary Figure 24. Saturated adsorption capacity of **PCN-222** and **PCN-222@PCCs** for methylene blue at 10 mg/L (a), 20 mg/L (b), 30 mg/L (c), 40 mg/L (d), 50 mg/L (e), and the saturation adsorption curves at different concentrations. Data are presented as the mean ± SD (f).

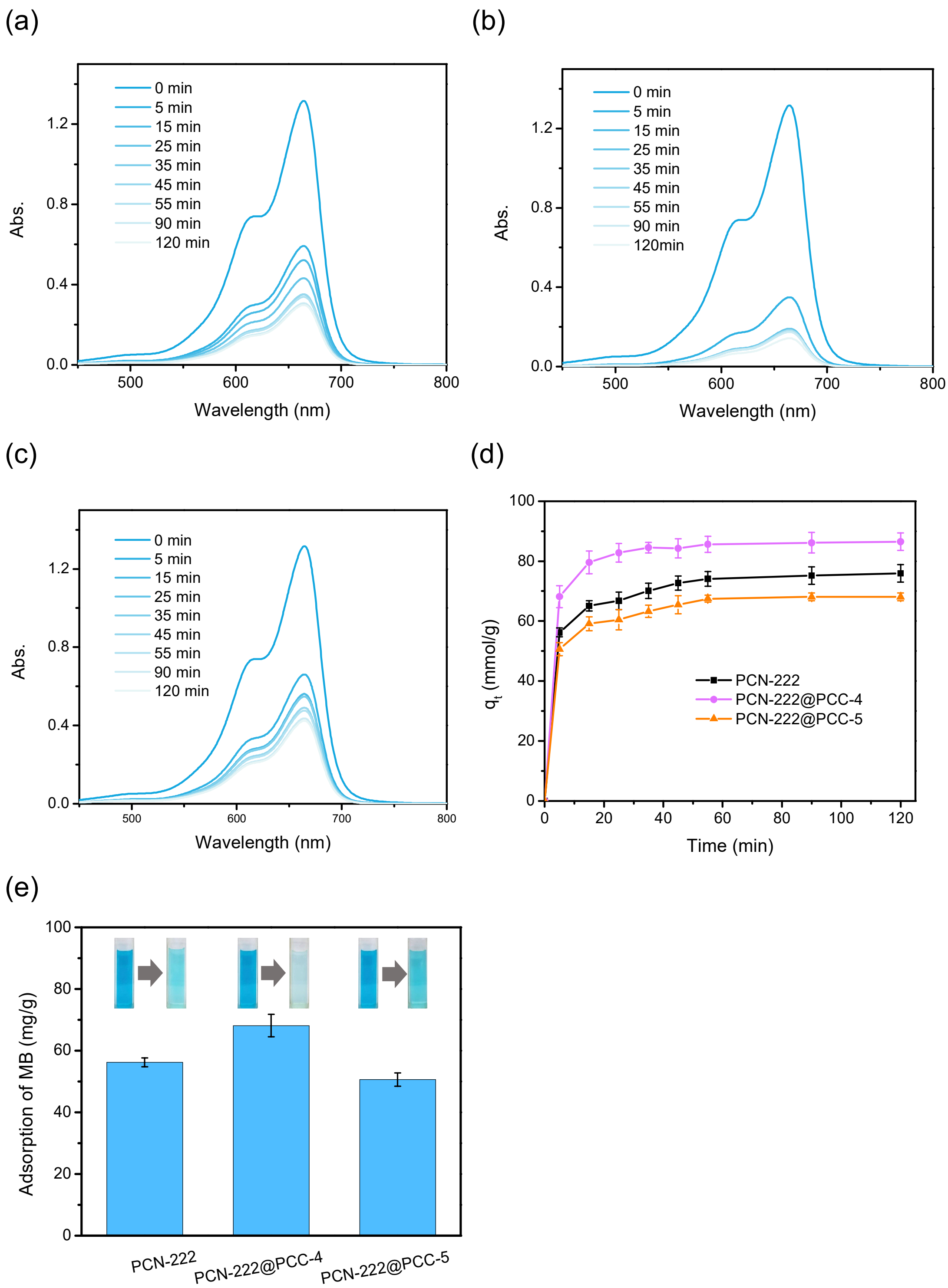

Supplementary Figure 25. The adsorption capacity of methylene blue by **PCN-222** (a), **PCN-222@PCC-4** (b), and **PCN-222@PCC-5** (c) at different times in 10 mg/L methylene blue aqueous solution; Time-dependent adsorption plot of three materials with time. Data are presented as the mean ± SD (d); the adsorption capacity of **PCN-222** and **PCN-222@PCCs** in 10 mg/L methylene blue aqueous solution within 5 min. Data are presented as the mean ± SD (e).

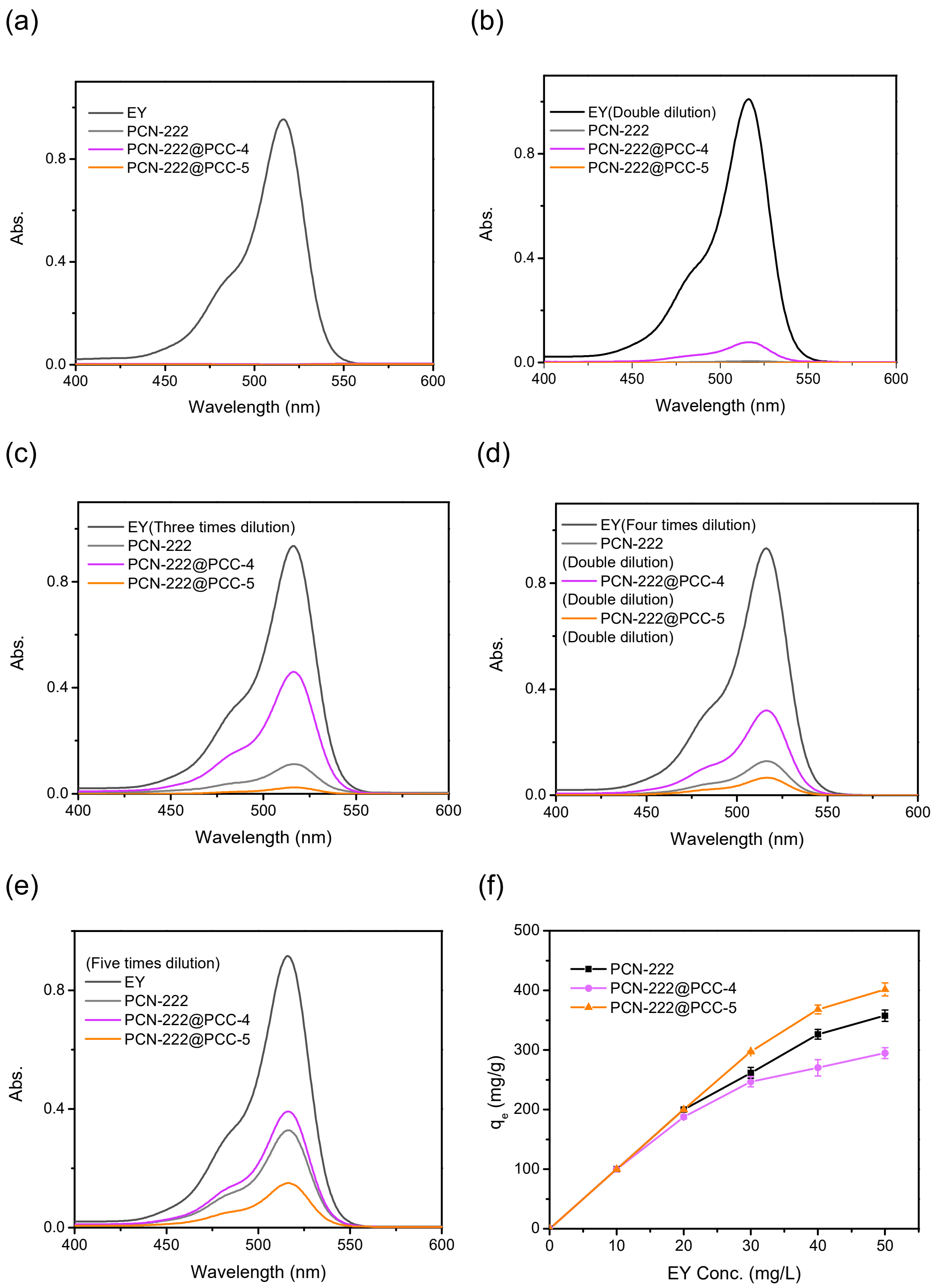

Supplementary Figure 26. The saturated adsorption capacity of **PCN-222** and **PCN-222@PCCs** for Eosin Y at 10 mg/L (a), 20 mg/L (b), 30 mg/L (c), 40 mg/L (d), 50 mg/L (e), and the saturation adsorption curves of **PCN-222** and **PCN-222@PCCs** at different concentrations. Data are presented as the mean ± SD (f).

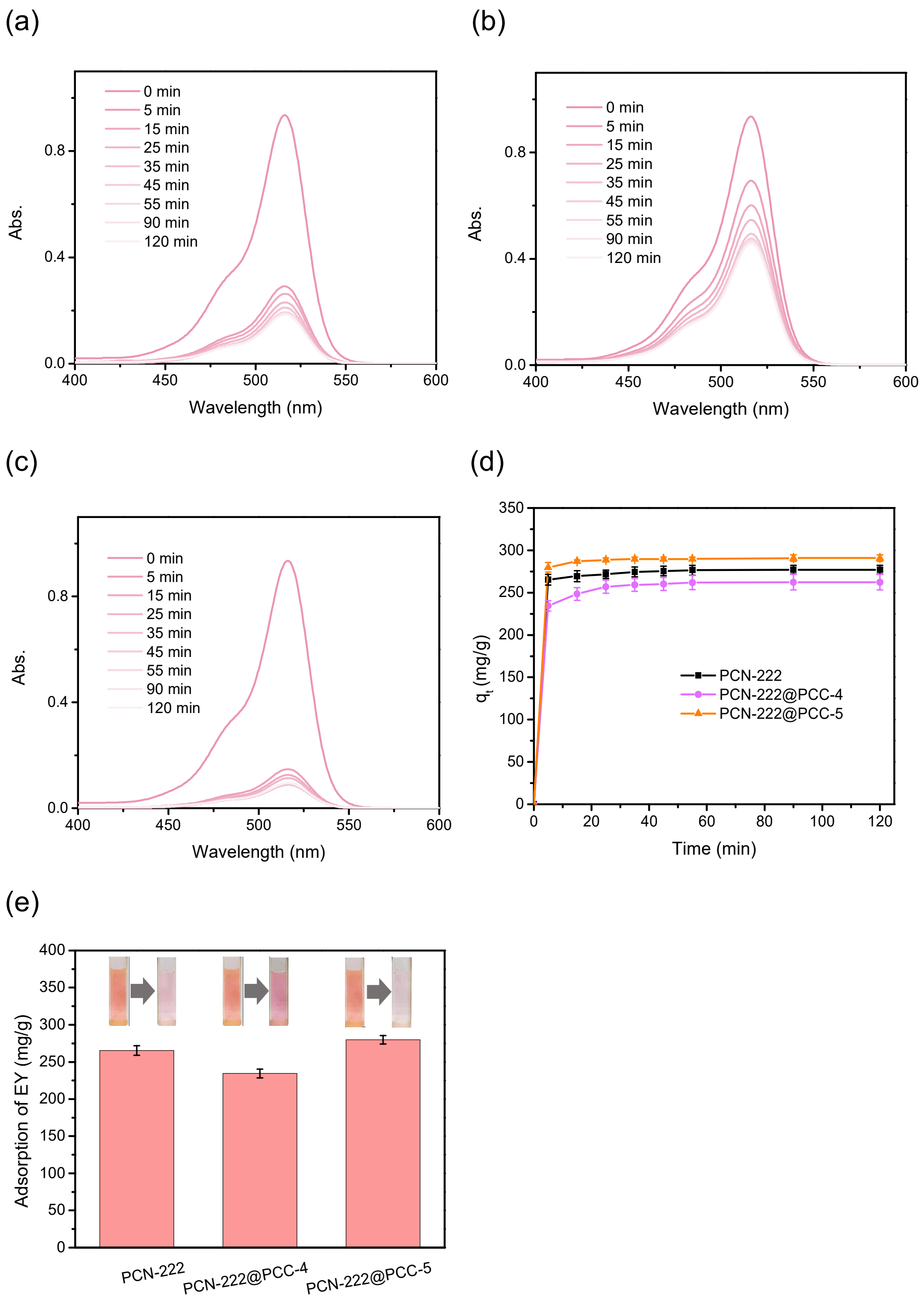

Supplementary Figure 27. The adsorption capacity of Eosin Y by **PCN-222** (a), **PCN-222@PCC-4** (b), and **PCN-222@PCC-5** (c) at different times in 30 mg/L eosin Y aqueous solution; adsorption curves of three materials with time. Data are presented as the mean ± SD (d); the adsorption capacity of **PCN-222** and **PCN-222@PCCs** in 30 mg/L methylene blue aqueous solution within 5 min. Data are presented as the mean ± SD (e).

(a)

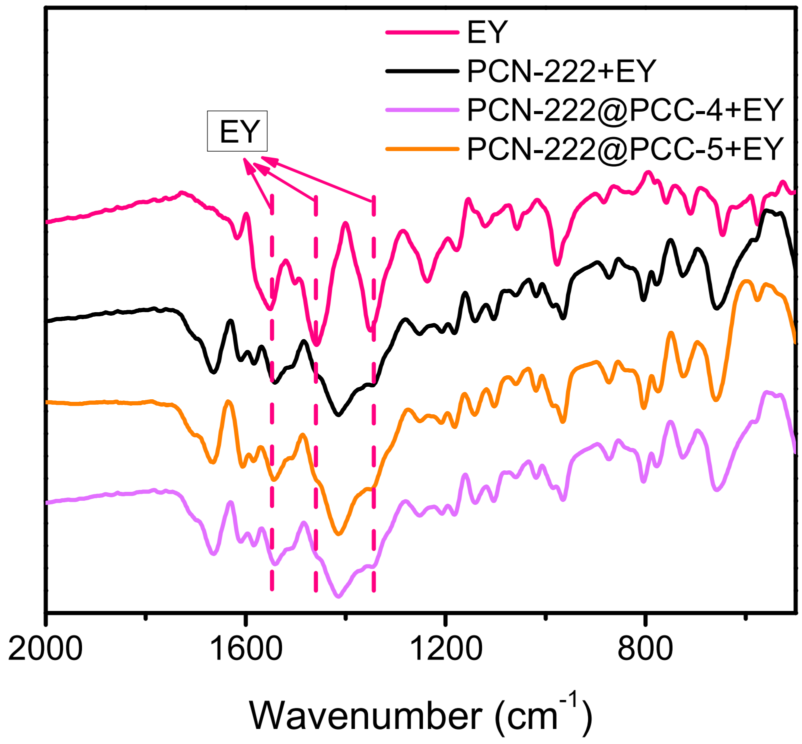

(b)

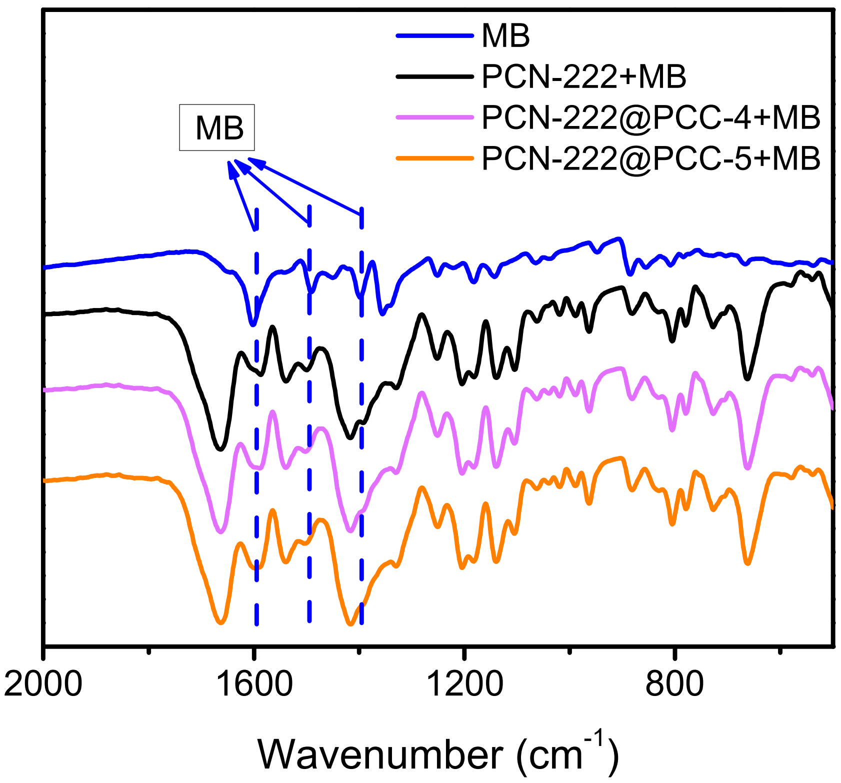

Supplementary Figure 28. The FT-IR spectra of **PCN-222@PCCs** after the dye adsorption. (a) After EY adsorption. (b) After MB adsorption.

To investigate the ability of **PCN-222@PCCs** to repeatedly adsorb dyes, we conducted the following experiments: 2 mg of **PCN-222@PCCs** were suspended in 40 mL of a cationic dye solution (Eosin Y and Methylene Blue) with a concentration of 40 mg/L. After allowing the mixture to stand for 2 hours, the solid was collected by centrifugation. Following dye adsorption, the **PCN-222@PCCs** were washed with MeCN and the solution was concentrated for UV-Vis analysis. As a control, pristine **PCN-222** was subjected to the same procedure for the two types of dye molecules. Our results showed that most cationic dyes could be efficiently desorbed, while only some of the anionic dyes could be removed. For Methylene Blue (cationic, +1), the release percentage for **PCN-222**, **PCN-222@PCC-4**, and **PCN-222@PCC-5** was 65.5%, 72.7%, and 63.0%, respectively. In contrast, for Eosin Y (anionic, -2), the release percentage for **PCN-222**, **PCN-222@PCC-4**, and **PCN-222@PCC-5** was 26.2%, 27.8%, and 34.7%, respectively. It was observed that **PCN-222** alone could not release all of the dyes due to the high surface area and porosity interacting with the adsorbed dye molecules, thereby retaining some of the encapsulated molecules within the surface or the pores.

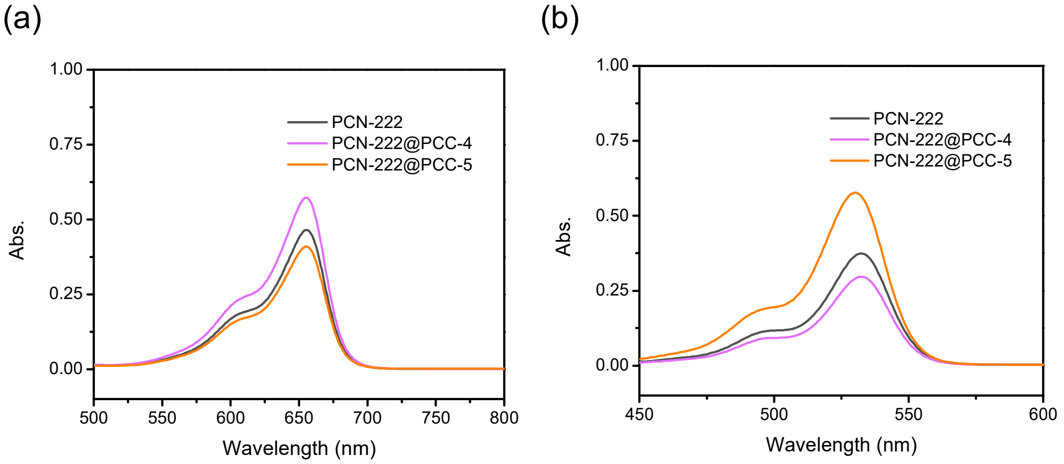

Supplementary Figure 29. UV-vis spectra of dye molecules released from **PCN-222@PCCs**. (a) MB. (b) EY.

Supplementary Table 4. Dye release from **PCN-222@PCCs**.

**Materials**	**Adsorption capacity of MB (mg)**	**The release of MB (mg)**	**Release rate of MB (%)**
PCN-222	0.29	0.19	65.5
PCN-222@PCC-4	0.33	0.24	72.7
PCN-222@PCC-5	0.27	0.17	63.0
**Materials**	**Adsorption capacity of EY (mg)**	**The release of EY (mg)**	**Release rate of EY (%)**
PCN-222	0.64	0.17	26.5
PCN-222@PCC-4	0.54	0.15	27.8
PCN-222@PCC-5	0.75	0.27	34.7

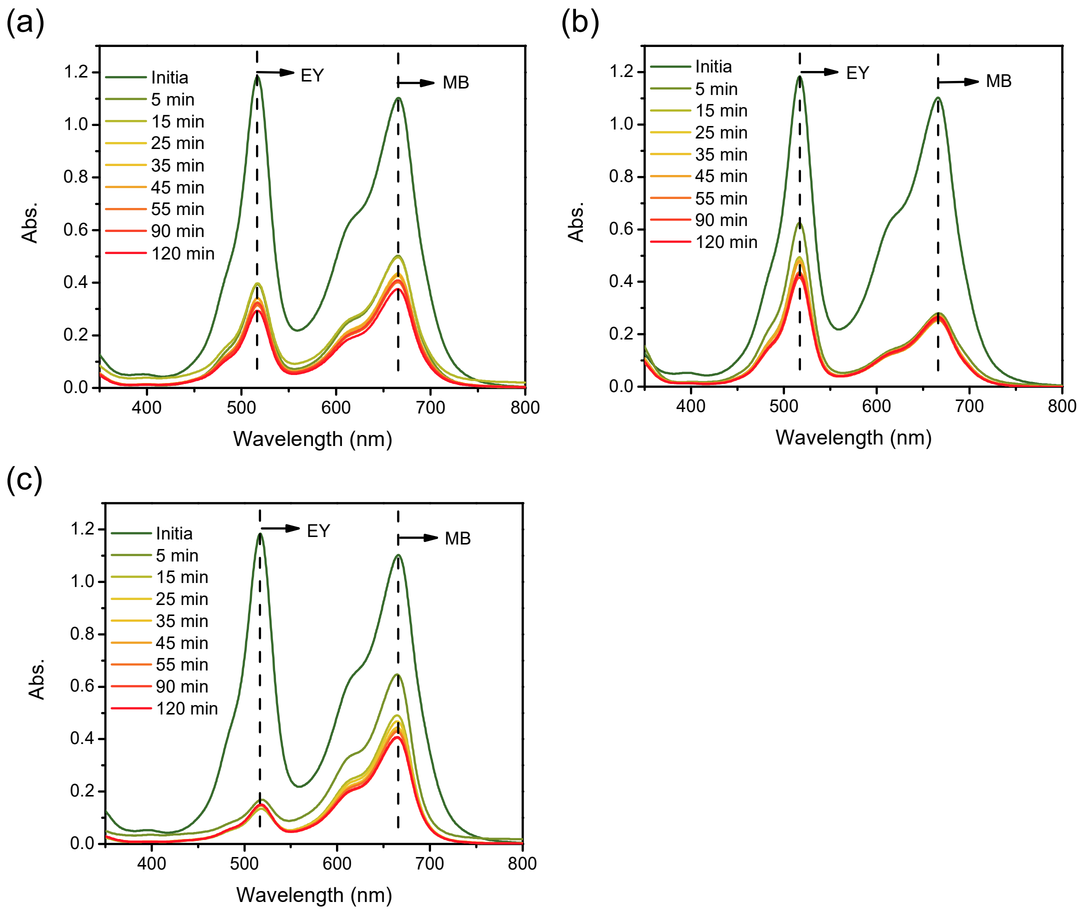

Supplementary Figure 30. Adsorption behavior of Rhodamine B and methyl orange mixed dyes by (a) **PCN-222**, (b) **PCN-222@PCC-4**, and (c) **PCN-222@PCC-5**.

Supplementary Table 5. The dye adsorption capacity of PCN-222 and **PCN-222@PCCs** in the first 5 min.

	**The total amount of dye adsorbed (mg/g)**	**The amount of MB (mg/g)**	**The amount of EY (mg/g)**
PCN-222	439.9	212.1	227.8
PCN-222@PCC-4	424.3	257.6	166.7
PCN-222@PCC-5	438.2	157.6	280.6

Section 6. Catalytic reactions of PCN-222@PCCs

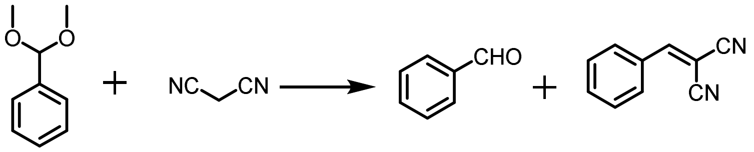

1 2 3 4

Supplementary Table 6. Catalytic activity and selectivity of **PCN-222** modified PCC components.

Entries	Catalyst	Conv. (%)	C Yield (%)	D Yield (%)
1	blank	13.4	3.2	10.2
2	PCC-4	7.7	2.1	5.6
3	PCC-5	100	77.8	22.2
4	PCN-222	100	100	52.7
5	PCN-222@PCC-4	100	24.6	75.4
6	PCN-222@PCC-5	100	89.7	10.3
7	PCN-222@PCC-2b	100	50.9	49.1
8	PCN-222@PCC-3	100	47.7	52.3
9	PCN-222@TSSC	100	48.3	51.7
10	PCN-222@Co	100	57.8	42.2
11	PCN-222@TATB	100	44.8	55.2
12	PCN-222@NH_2- Bipyridine	100	18.4	81.6
13	PCN-222@Pd	100	52.3	47.7
14	PCN-222@TPT	100	45.7	54.3
15	PCN-222 mix with PCC-4	100	50.9	49.1
16	PCN-222 mix with PCC-5	100	49.8	50.2

Reaction condition: Benzaldehyde dimethyl acetal (1.33 mmol), malononitrile (2.0 mmol), CH_3CN: H_2O =17: 3 (10 mL), 10 mg catalyst, 50 °C for 5 h.

(a)

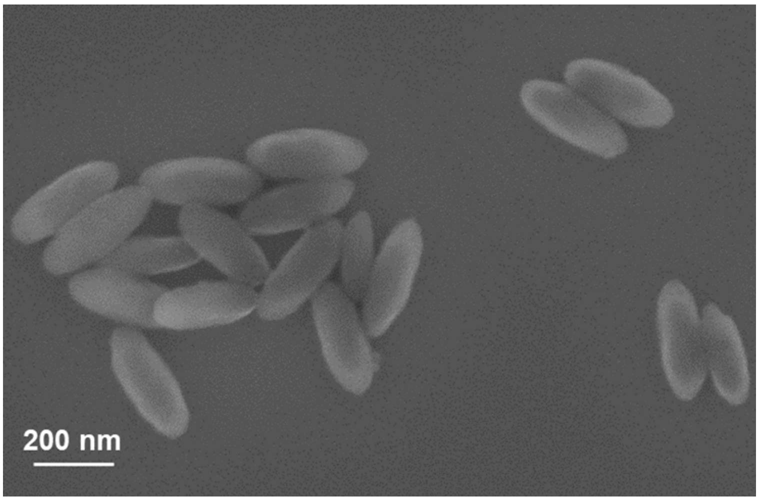

(b)

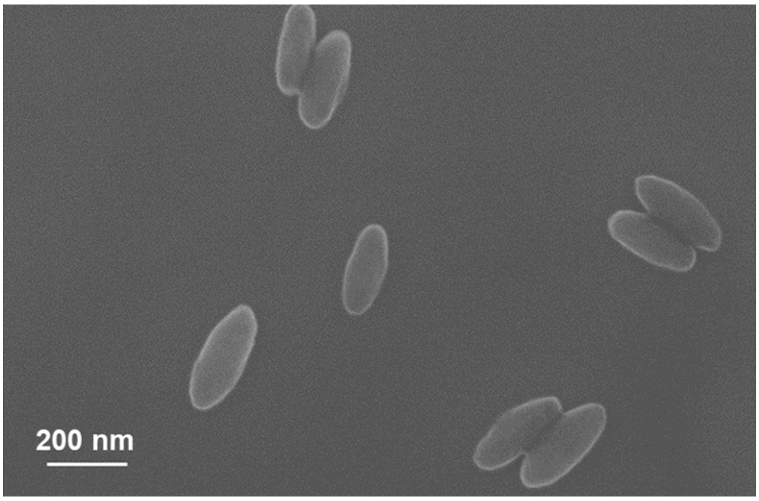

Supplementary Figure 31. SEM images of **PCN-222@PCC-4** (a) and **PCN-222@PCC-5** (b) after 10 times of recycling. Scale bar = 200 nm.

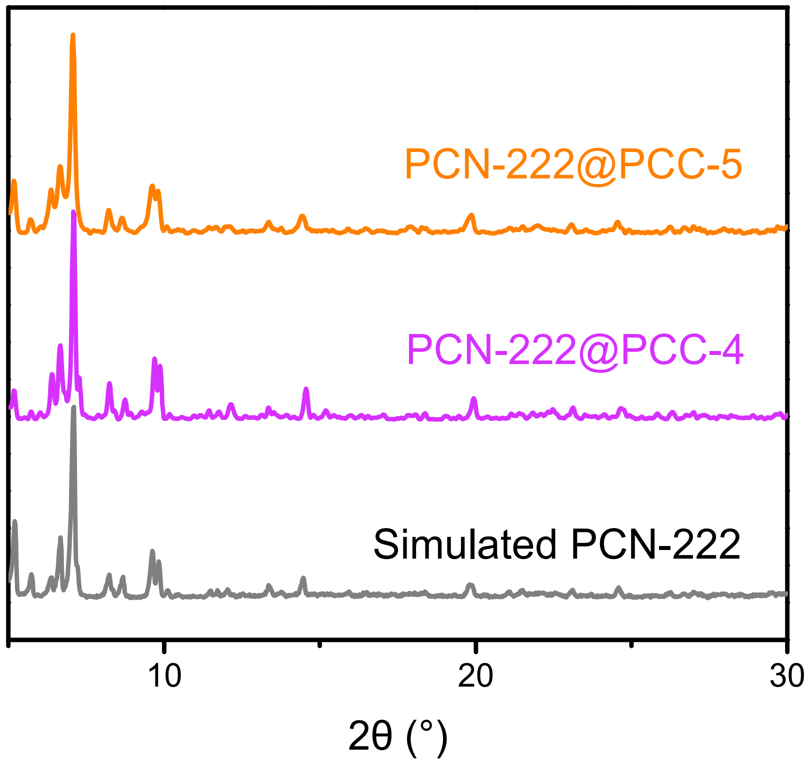

Supplementary Figure 32. PXRD patterns of **PCN-222@PCC-4** and **PCN-222@PCC-5** after 10 times of recycling.

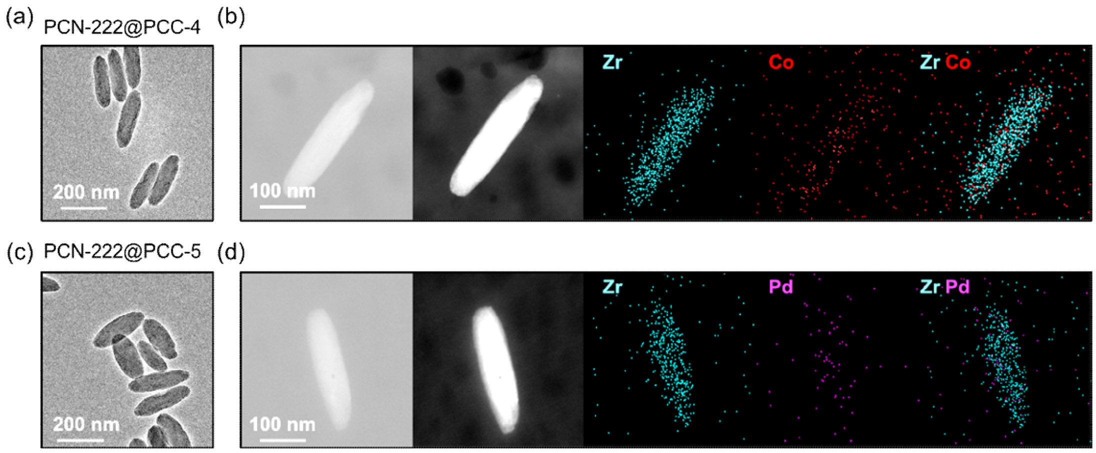

Supplementary Figure 33. The STEM images of **PCN-222@PCCs** after 10 times of recycling.

Supplementary Table 7. ICP-OES data of **PCN-222@PCCs** after 10 cycles.

	V_0 （mL)	C_{Zr} (mg/L)	C_{Co} (mg/L)	C_{Pd} (mg/L)	PCC/PCN-222@PCC (wt%)	The amount of PCC loss (wt%)
PCN-222@PCC-4	10	10.20	0.29	/	5.12	8.9
PCN-222@PCC-5	10	10.02	/	0.10	1.68	15.2

Subsequently, the reaction mechanism of **MOF@PCC** was investigated by time-dependent nuclear magnetic resonance (NMR) and gas chromatography (GC), with the purpose of providing a clear understanding of the reaction profile catalyzed by each component. To achieve this, four catalysts were evaluated separately, namely no catalyst (blank), **PCN-222**, **PCC-4**, and **PCC-5**. The first step of the sequential reaction (Supplementary Fig. 34a) was monitored by observing the conversion of the substrate to the product through the decrease of "proton a" and the increase of "proton b" (Supplementary Figs. 34b-34e). From the time-dependent conversion plot (Supplementary Fig. 34f), it was evident that the reaction could only proceed up to 25.6% after 5 hrs without a catalyst (blank). However, when **PCC-4** was used as the catalyst, the conversion was reduced to 15.4% after 5 hrs, indicating that the anionic cage inhibited the reaction. Conversely, both **PCN-222** and **PCC-5** significantly enhanced the reactivity by achieving 100% conversion within 4 hrs, demonstrating the promotion of reactivity. In comparison to **PCN-222**, the cationic **PCC-5** reached quantitative yield within 2 hrs, exhibiting faster kinetics than **PCN-222**.

The second step of the sequential reaction (Supplementary Fig. 34g) was monitored by observing the conversion of the substrate to the product through the decrease of "proton b" and the increase of "proton c" (Supplementary Figs. 34h-34k). From the time-dependent conversion plot (Supplementary Fig. 34l), it was evident that the reaction could only proceed up to 46.6% after 5 hrs without a catalyst (blank). However, when **PCC-5** was used as the catalyst, the conversion was reduced to 4.1% after 5 hrs, demonstrating that the cationic cage inhibited the reaction. The **PCN-222** catalyst yielded a moderate conversion rate of 56.8% after 5 hrs. On the other hand, when anionic **PCC-4** was used as the catalyst, the conversion was increased to 81.1% after 3 hrs, indicating the enhancement of reactivity. By closely examining the reaction profile (Supplementary Figs. 34f and 34l), it was demonstrated that anionic **PCC-4** inhibited the first step but promoted the second step, whereas cationic **PCC-5** enhanced the first step but inhibited the second step. Thus, by utilizing different catalysts in the individual steps of the sequential reaction, PCC was identified as the origin of product selectivity.

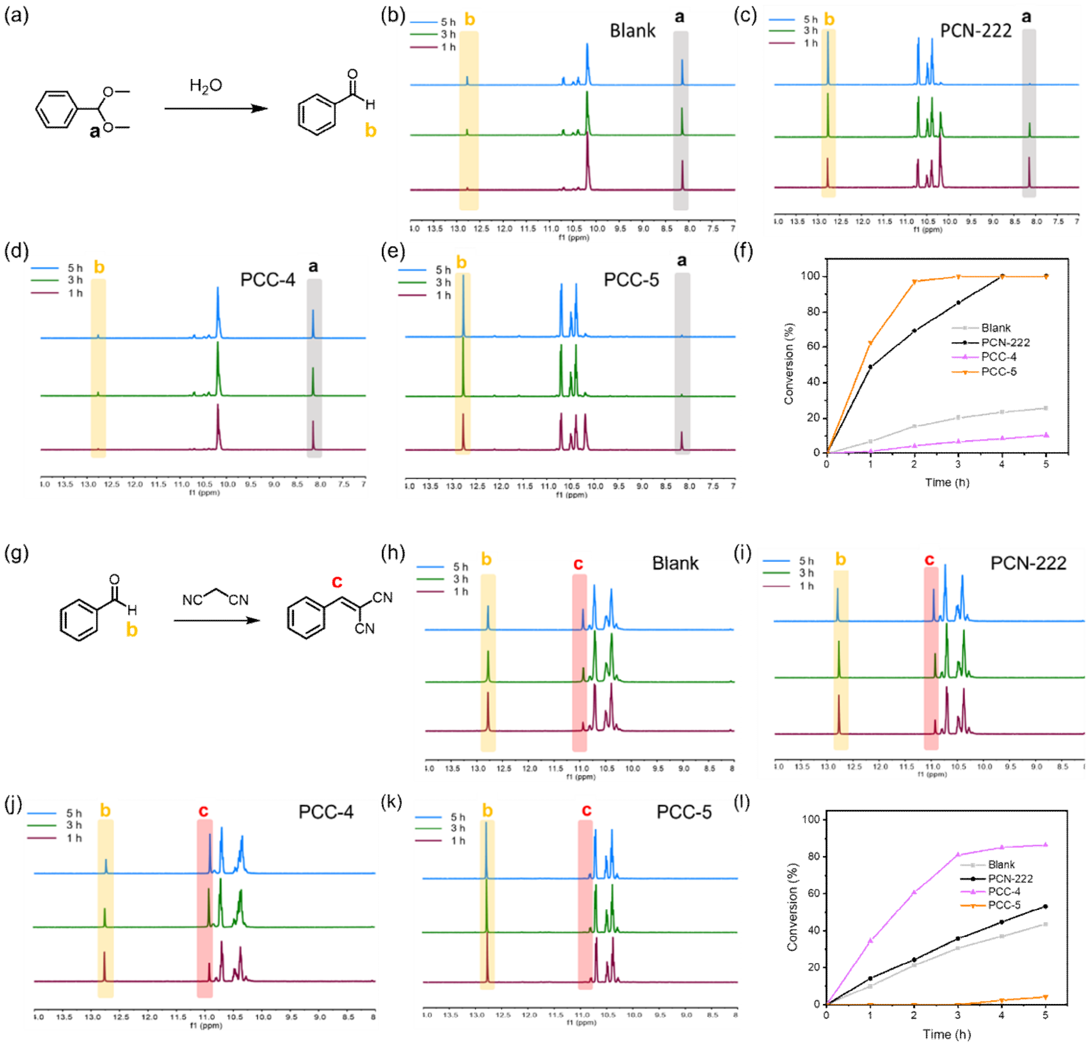

Supplementary Figure 34. Time-dependent reaction profiles of the sequential catalytic reaction monitored by NMR and GC. (a) Reaction scheme of the first step of the sequential reaction. NMR of the reaction profile after 1h, 3 h, and 5 h in the presence of blank (b), **PCN-222** (c), **PCC-4** (d), and **PCC-5** (e). (f) Substrate conversion plot in the presence of the different catalysts. (g) Reaction scheme of the second step of the sequential reaction. NMR of the reaction profile after 1h, 3 h, and 5 h in the presence of blank (h), **PCN-222** (i), **PCC-4** (j), and **PCC-5** (k). Substrate conversion plot in the presence of a different catalyst (l).

(a) **PCN-222**

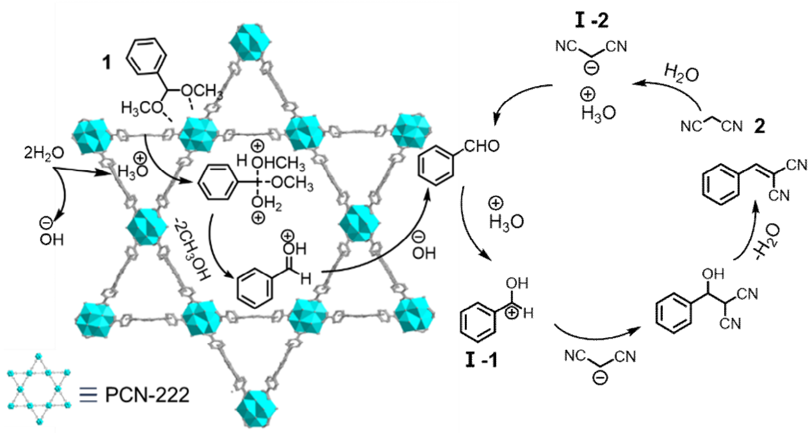

(b) **PCN-222@PCC-4**

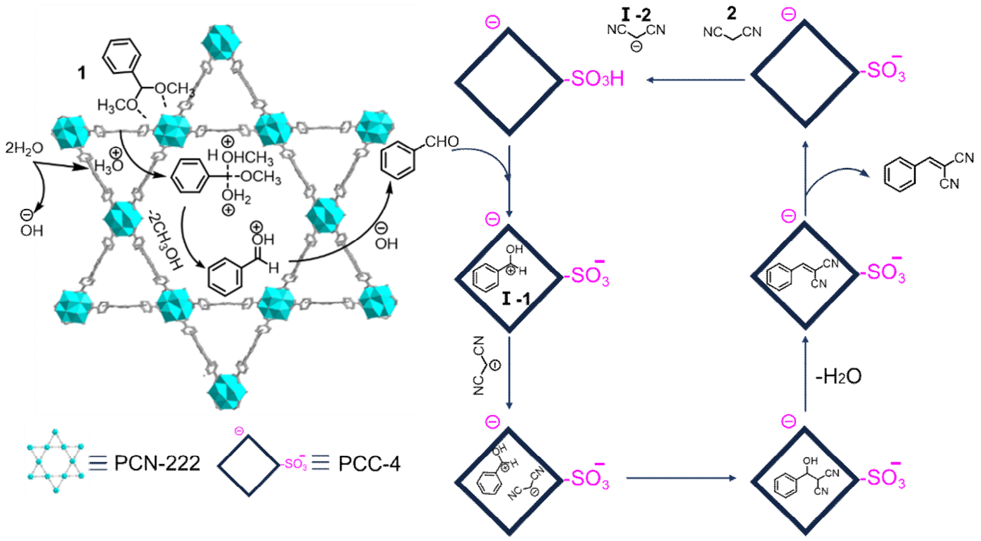

(c) **PCN-222@PCC-5**

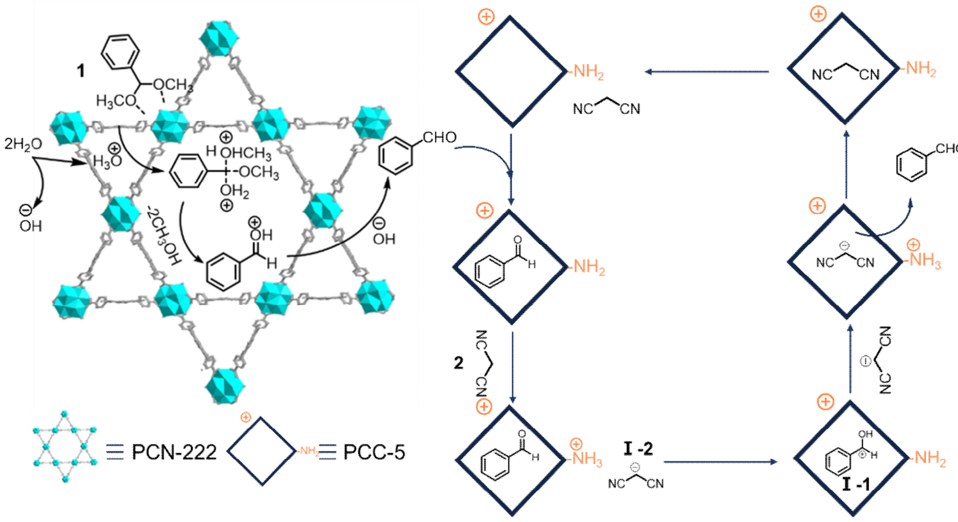

Supplementary Figure 35. Diagram of the proposed catalytic reaction mechanism of **PCN-222** (a), **PCN-222@PCC-4** (b), **PCN-222@PCC-5** (c).

Acid treatment and re-introduction of PCC-5: The **PCN-222@PCCs** were subjected to a surface etching procedure by placing them in a 1 M aqueous hydrochloric acid solution and soaking them for 2 hours with stirring. The resulting solids were collected by centrifugation, and the supernatant was repeatedly washed with deionized water until neutral pH was achieved. Subsequently, the obtained solid was immersed in DMF and soaked for 12 hours. The solid was then collected by centrifugation and washed twice with DMF to obtain **PCN-222**. The synthesis of **PCN-222@PCCs** was then carried out as described previously.

(a)

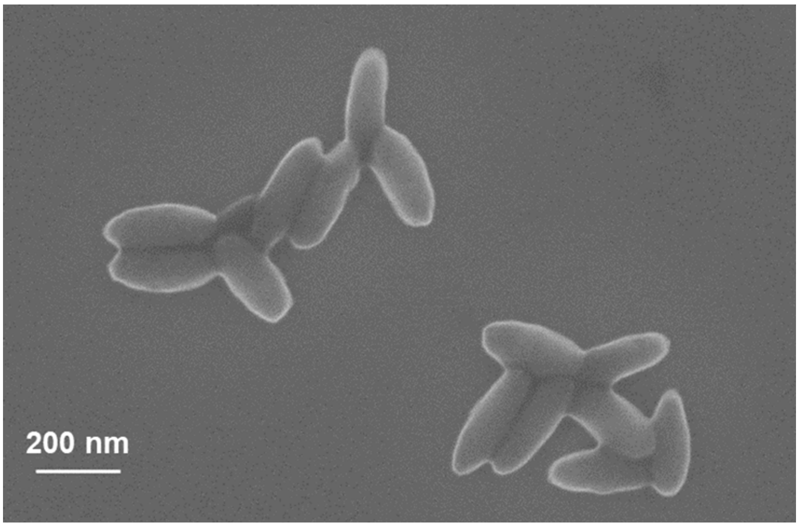

(b)

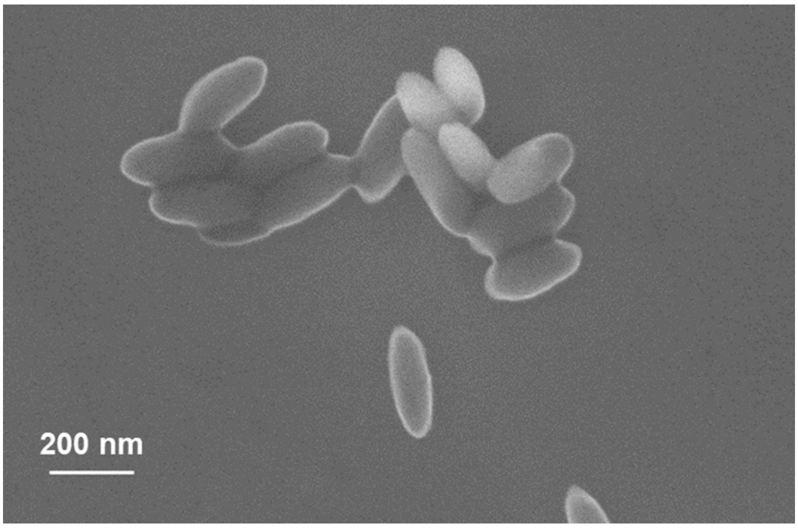

Supplementary Figure 36. SEM of **PCN-222@PCC-4** (a) and **PCN-222@PCC-5** (b) after reproducible modification. Scale bar = 200 nm.

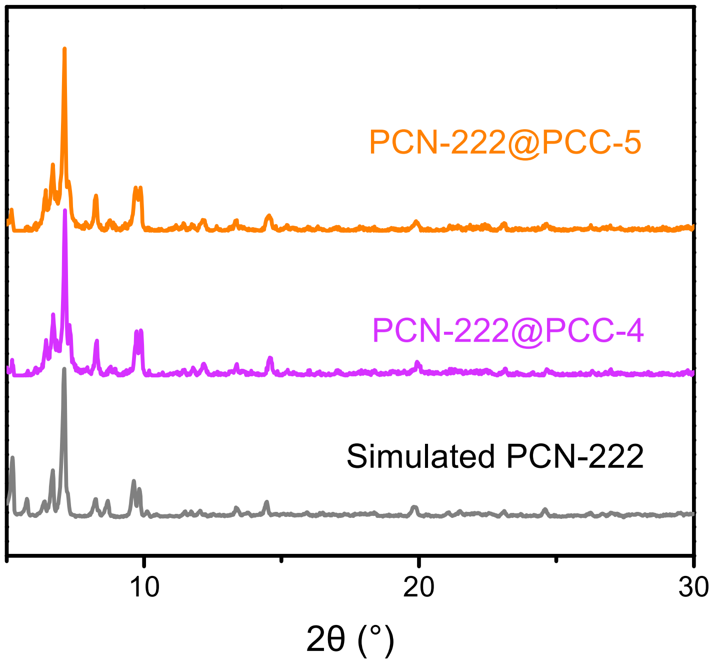

Supplementary Figure 37. PXRD of **PCN-222@PCCs** after reversible modification.

Supplementary Table 8. ICP-OES data of **PCN-222@PCCs** after surface etched.

	V_0 （mL）	C_{Zr} (mg/L)	C_{Co} (mg/L)	C_{Pd} (mg/L)	Co/Zr	Pd/Zr
PCN-222@PCC-4	10	24.24	0.76	/	0.031	/
Surface etched PCN-222@PCC-4	10	16.81	0.17	/	0.010	/
PCN-222@PCC-5	10	21.97	/	0.26	/	0.013
Surface etched PCN-222@PCC-5	10	14.97	/	0.09	/	0.006

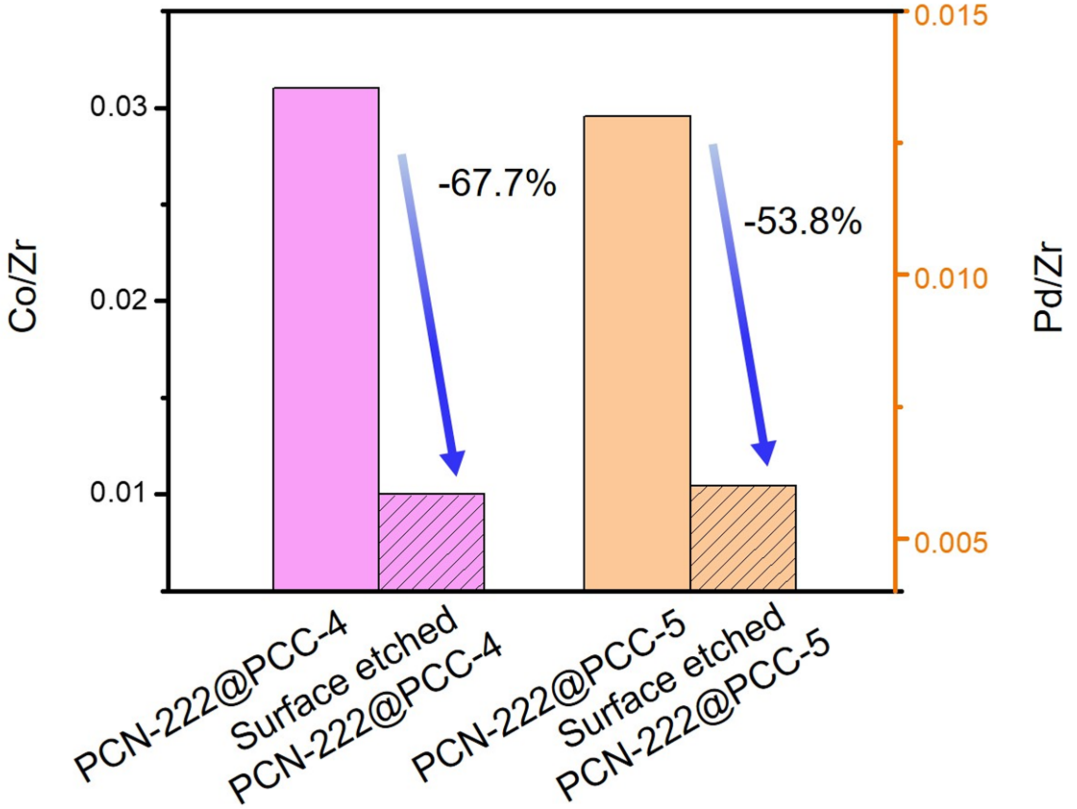

Supplementary Figure 38. The ratio Co/Zr of **PCN-222@PCC-4** before and after surface etching, and the ratio Pd/Zr of **PCN-222@PCC-5** before and after surface etching.

Section 7. Characterizations of MIL-101@PCCs

(a)

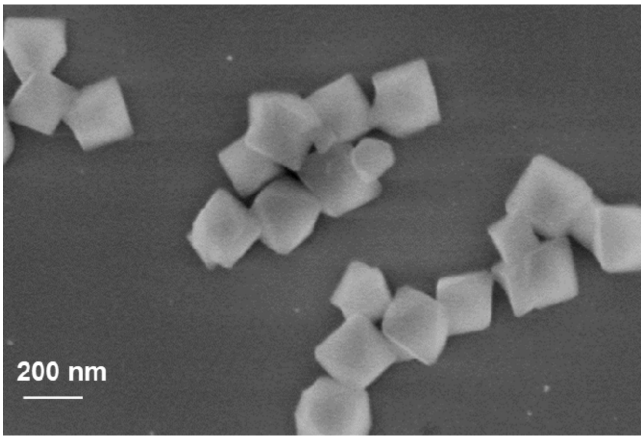

(b)

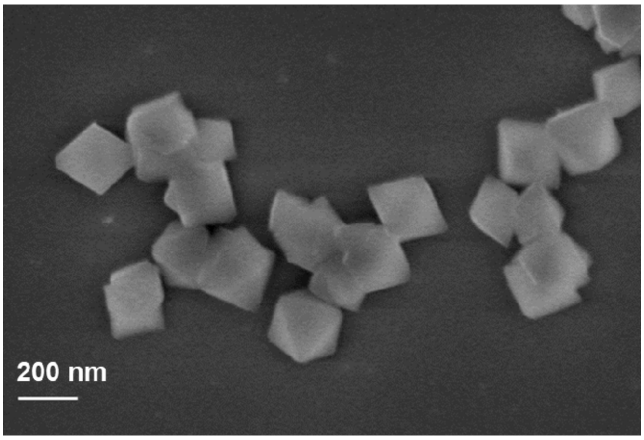

©

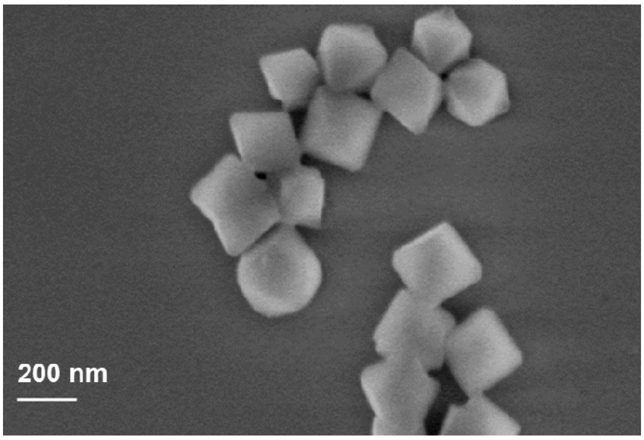

Supplementary Figure 39. SEM images of **MIL-101** (a), **MIL-101@PCC-4** (b), and **MIL-101@PCC-5** (c). Scale bar = 200 nm.

(a)

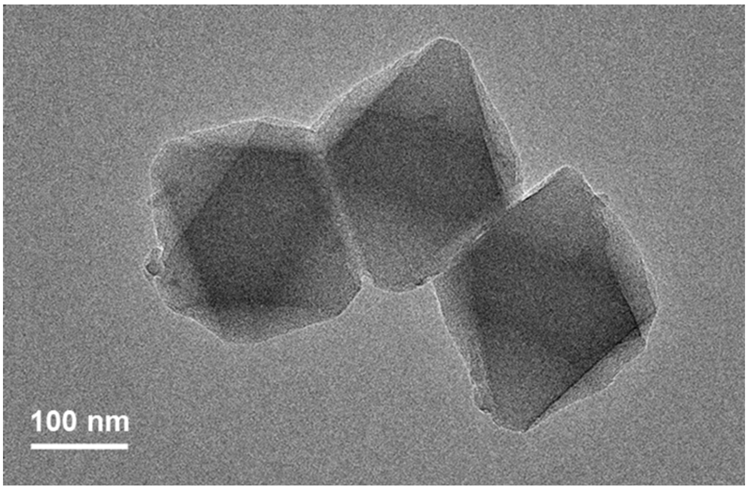

(b)

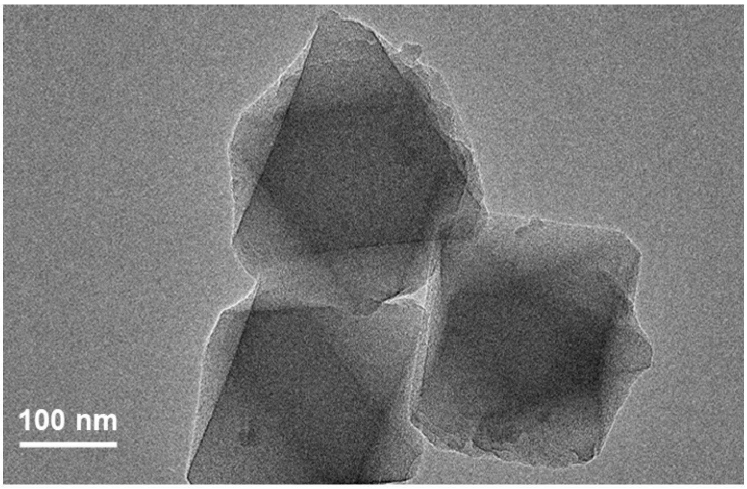

(c)

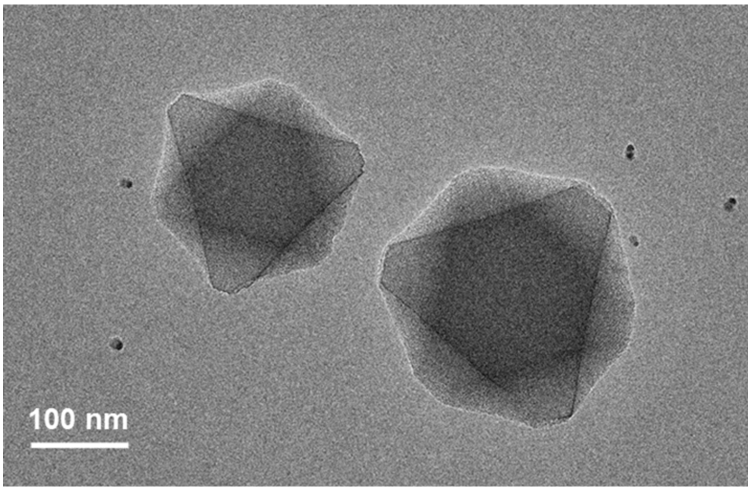

Supplementary Figure 40. TEM of **MIL-101** (a), **MIL-101@PCC-4** (b), and **MIL-101@PCC-5** (c). Scale bar = 100 nm.

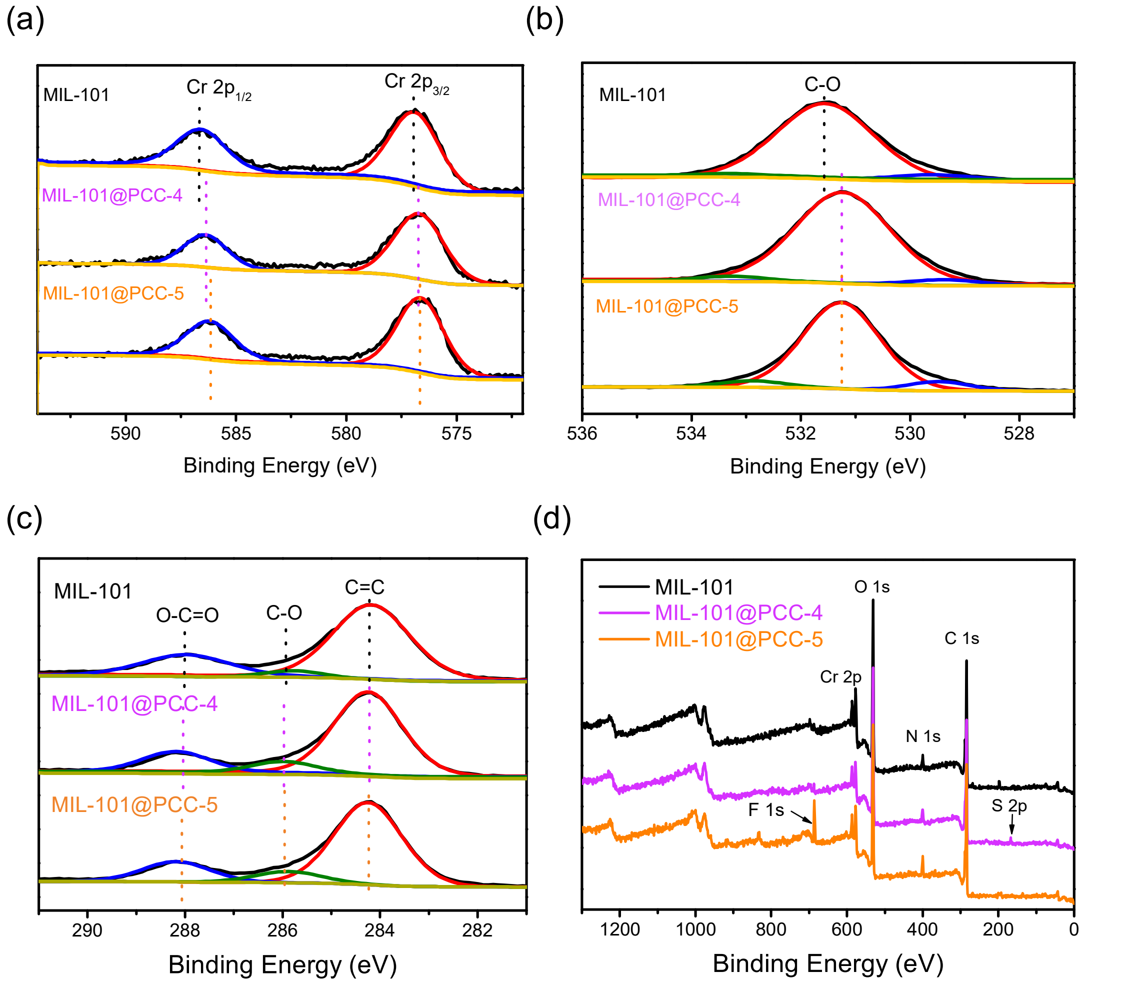

Supplementary Figure 41. *XPS* spectra of **MIL-101** and **MIL-101@PCCs**. (a) Cr 2*p*, (b) O 1s, (c) C 1*s*, and (d) survey spectra.

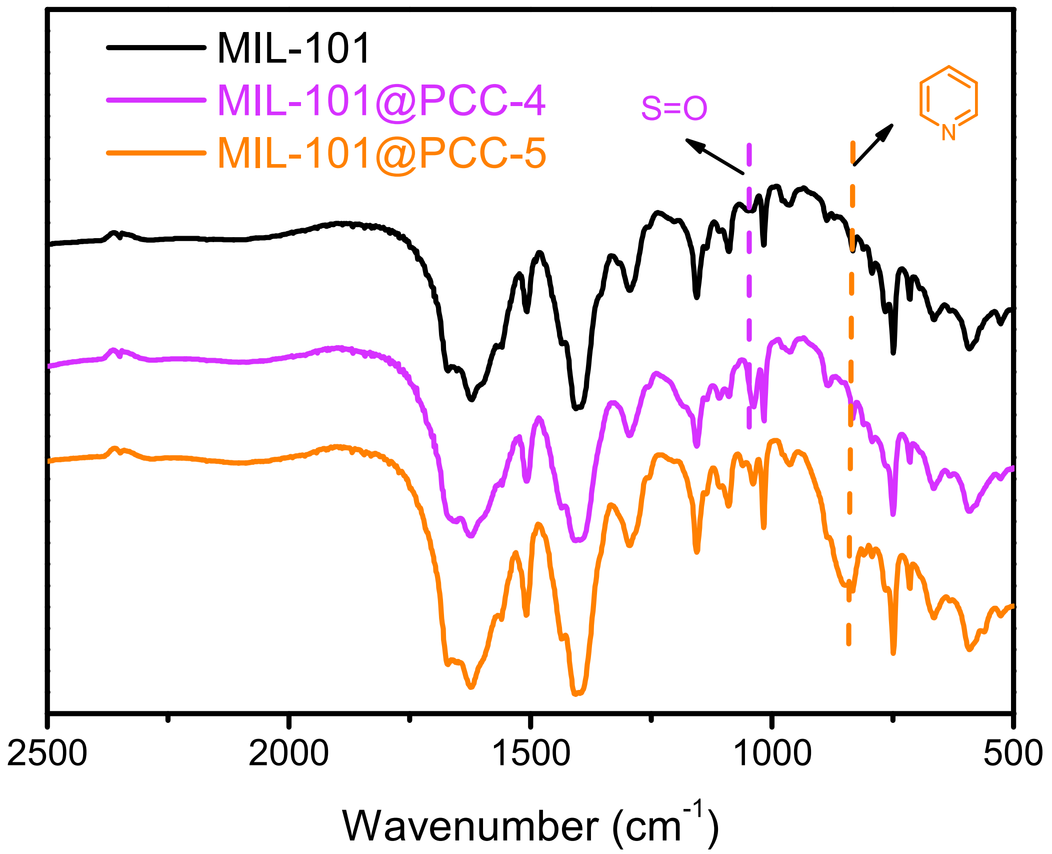

Supplementary Figure 42. FT-IR of **MIL-101** and **MIL-101@PCCs**.

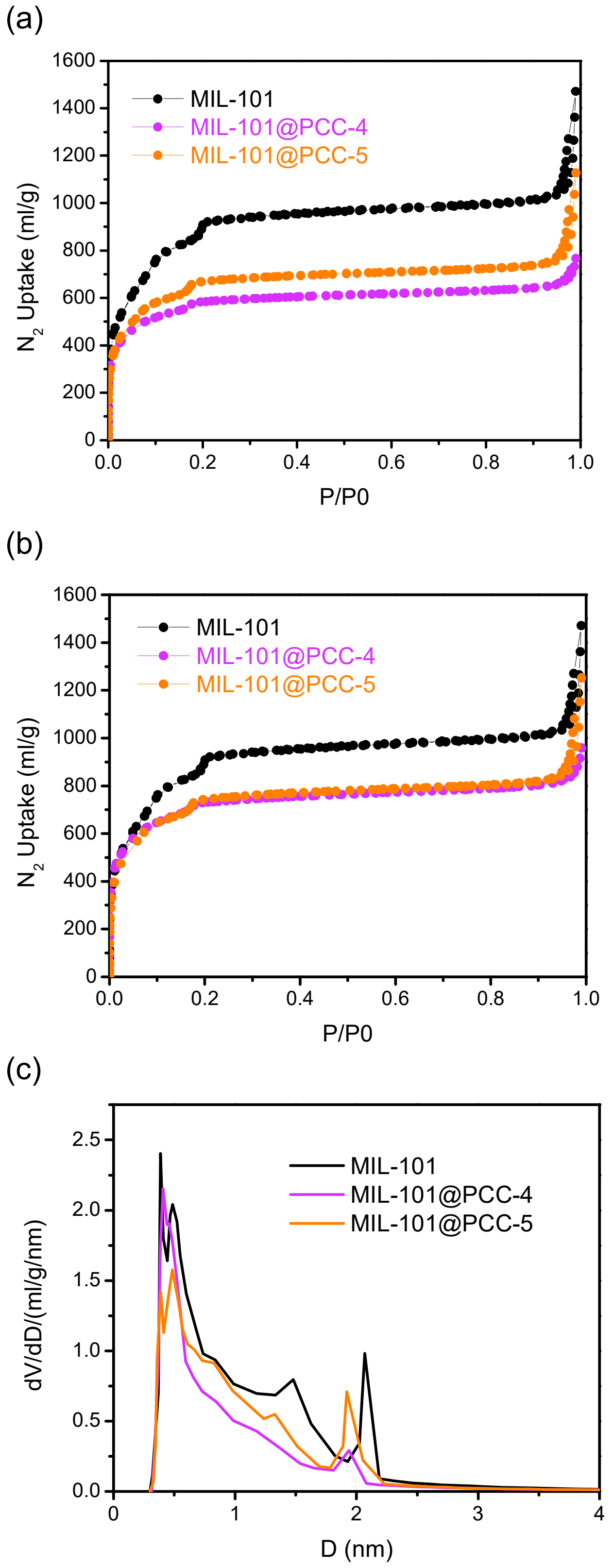

Supplementary Figure 43. N_2 adsorption isotherms of **MIL-101@PCCs** (a) and N_2 adsorption isotherms of **MIL-101@PCCs** after normalization (b), and the pore size distribution of **MIL-101@PCCs** (c) at 77 K.

For The characterization of **MIL-101@PCC-5** at temperatures ranging from 25-60°C did not reveal any peaks corresponding to **PCC-4**, indicating no leaching or decomposition of **PCC-4** (Supplementary Figs. 44-46). However, at 80°C, a small amount of intact **PCC-4** and **PCC-5** was detected in the supernatant. Further elevation of temperature to 100°C resulted in the detection of a characteristic peak corresponding to decomposed **PCC-4** (Supplementary Fig. 45). These findings suggest that **MOF@PCCs** can maintain their structural integrity between 25-80°C but undergo gradual decomposition at 100°C.

(a)

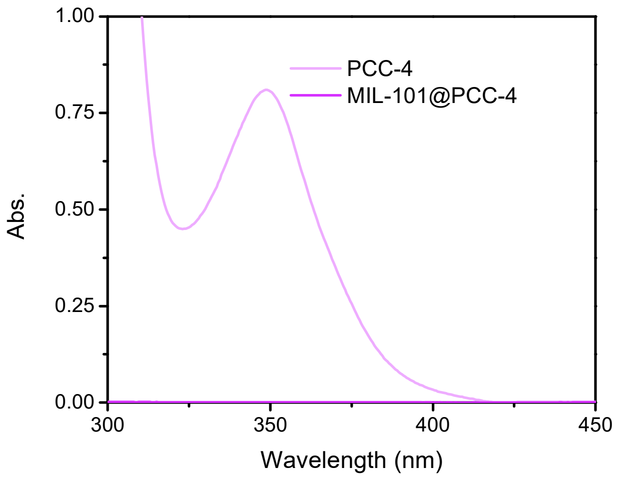

(b)

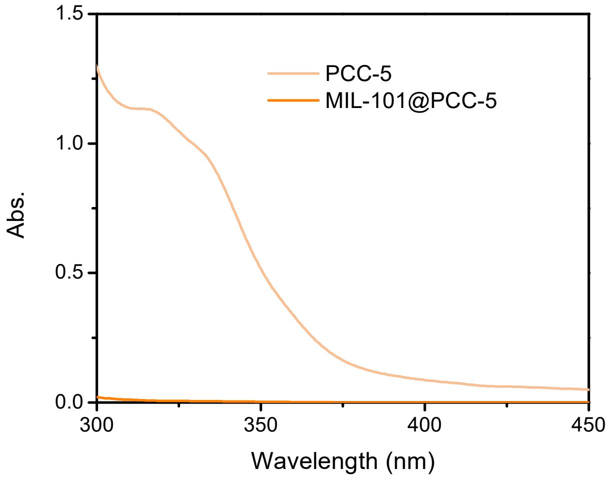

Supplementary Figure 44. **MIL-101@PCC-4** was immersed in DMF and the supernatant was detected after 24 h (a). **MIL-101@PCC-5** was immersed in CH_3CN and the supernatant was detected after 24 h (b).

(a)

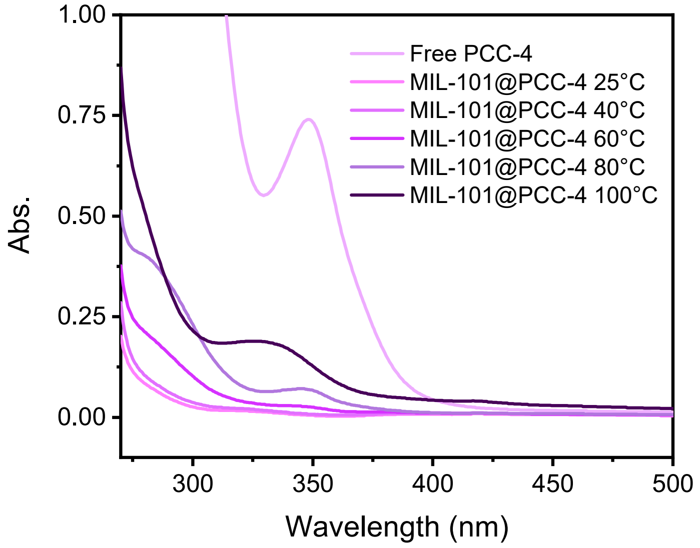

(b)

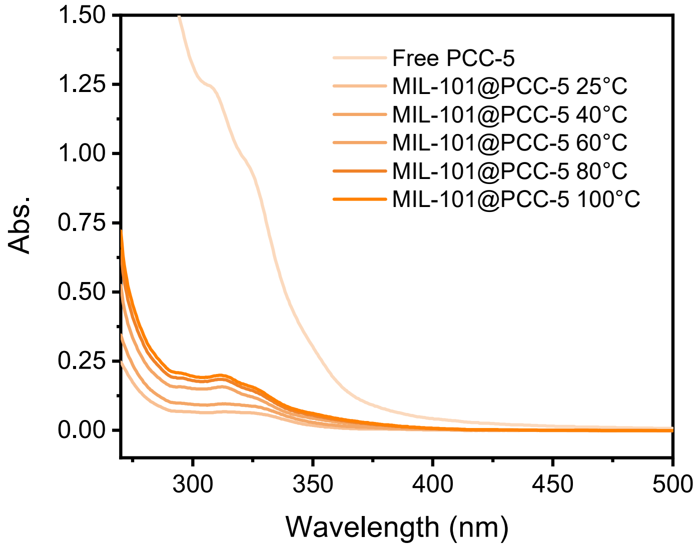

Supplementary Figure 45. The stability of **MIL-101@PCCs** at different temperatures.

We utilized UV-vis spectroscopy to investigate the state of the **MIL-101@PCCs** solution. To investigate the stability of the PCCs in the **MIL-101@PCC** composite, we attempted to separate the PCCs from the surface of **MIL-101@PCC** by sonicating the composite and analyzing the supernatant solution with UV-vis measurements. Our results, as illustrated in Supplementary Fig. 46, demonstrated that the characteristic peak of the separated PCC did not change, indicating that the PCC remained stable in the form of **MIL-101@PCC** and was not released into the solution.

(a)

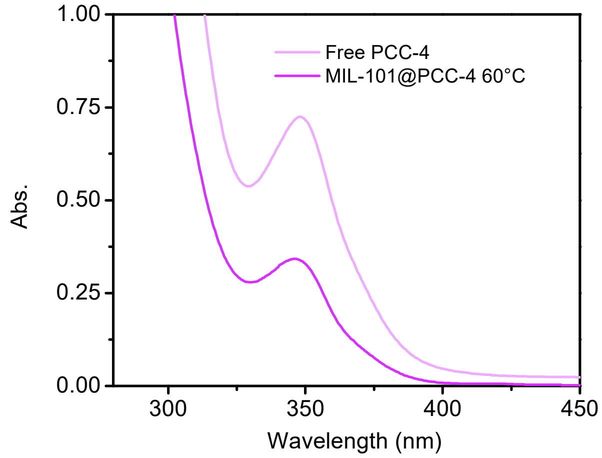

(b)

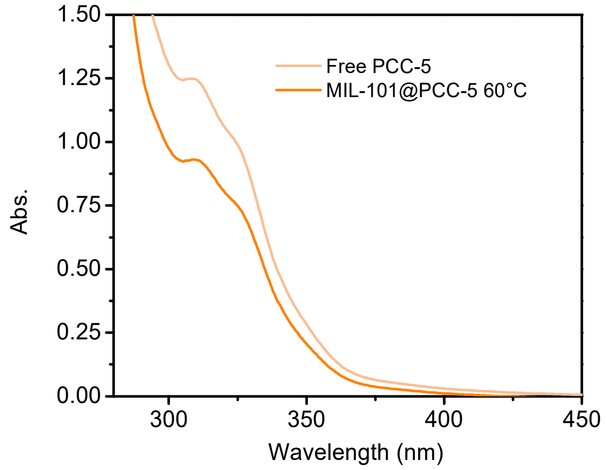

Supplementary Figure 46. UV detection of the state of the stripped **PCCs**.

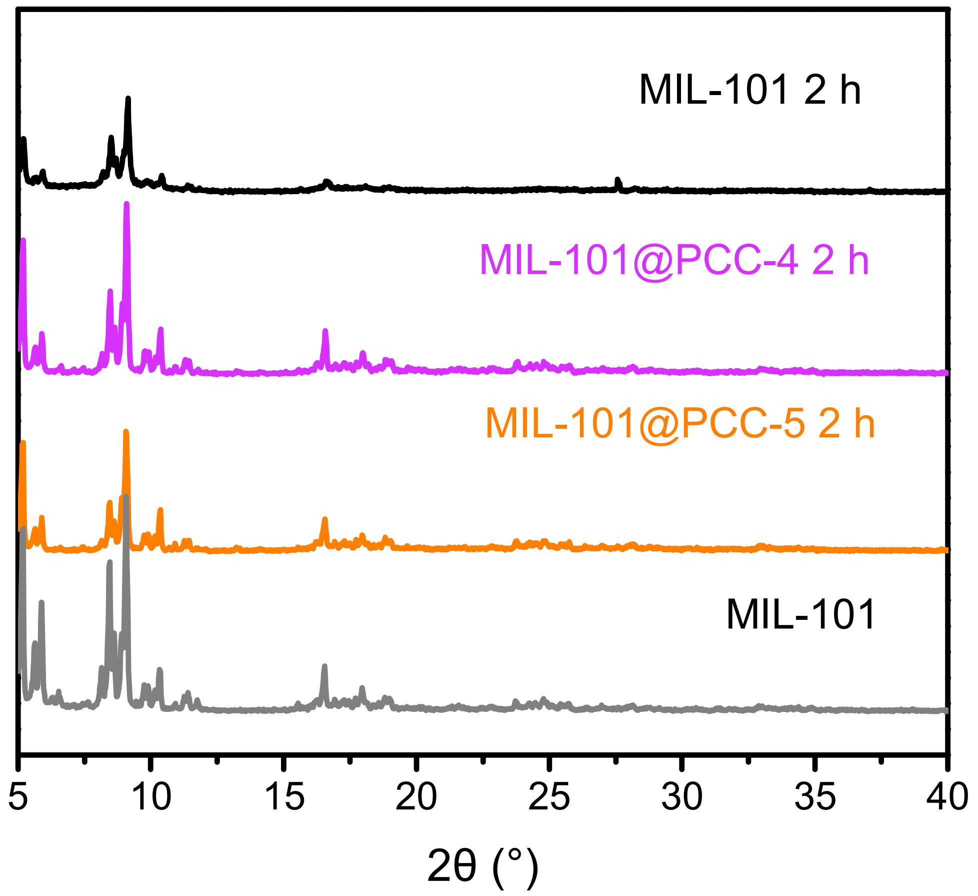

Supplementary Figure 47. Alkali stability of **MIL-101@PCCs** (condition: the materials were immersed in 0.04 M KOH aqueous solution).

(a)

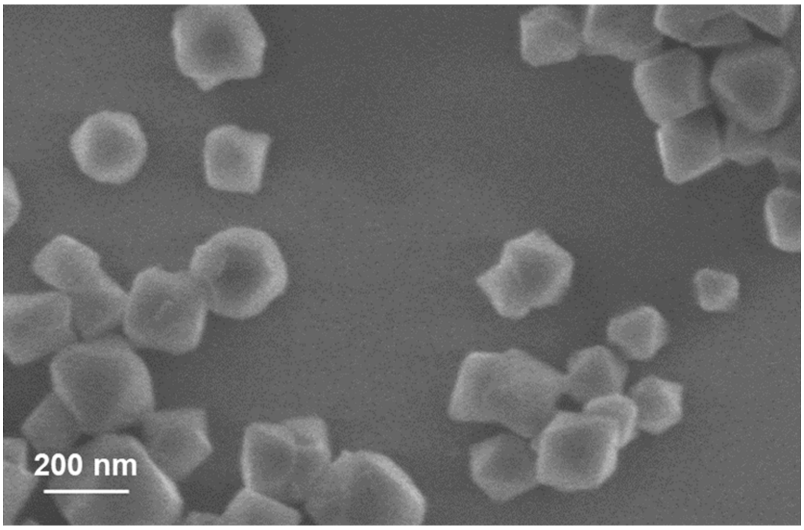

(b)

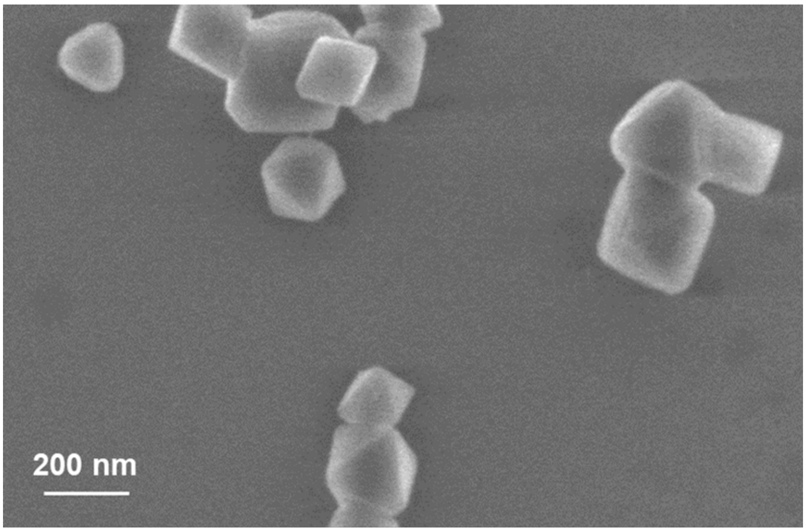

Supplementary Figure 48. SEM images of **MIL-101@PCC-2b** (a) and **MIL-101@PCC-3** (b). Scale bar = 200 nm.

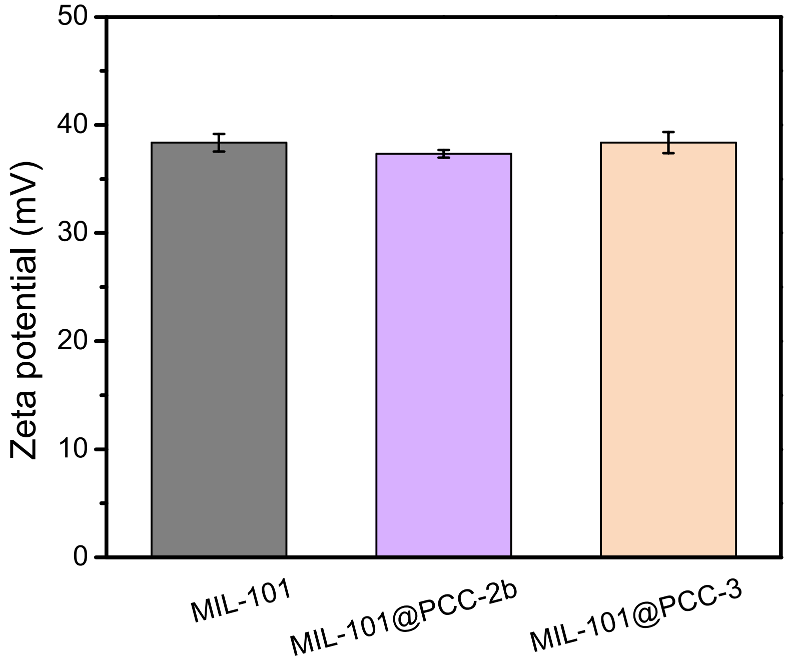

Supplementary Figure 49. Zeta potential of **MIL-101**, **MIL-101@PCC-2b** and **MIL-101@PCC-3**. Data are presented as the mean ± SD.

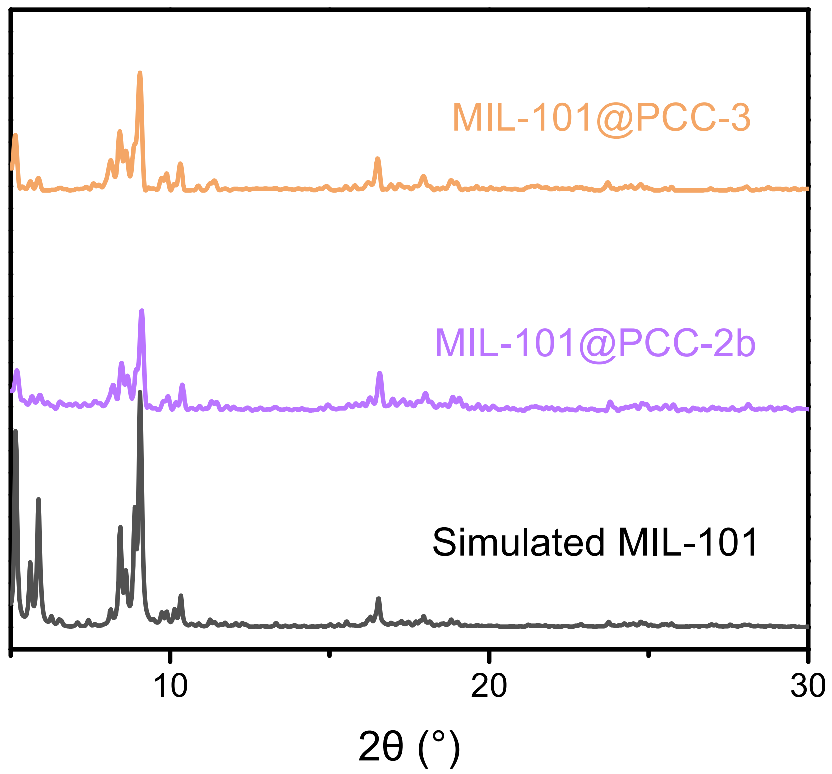

Supplementary Figure 50. PXRD of **MIL-101**, **MIL-101@PCC-2b,** and **MIL-101@PCC-3**.

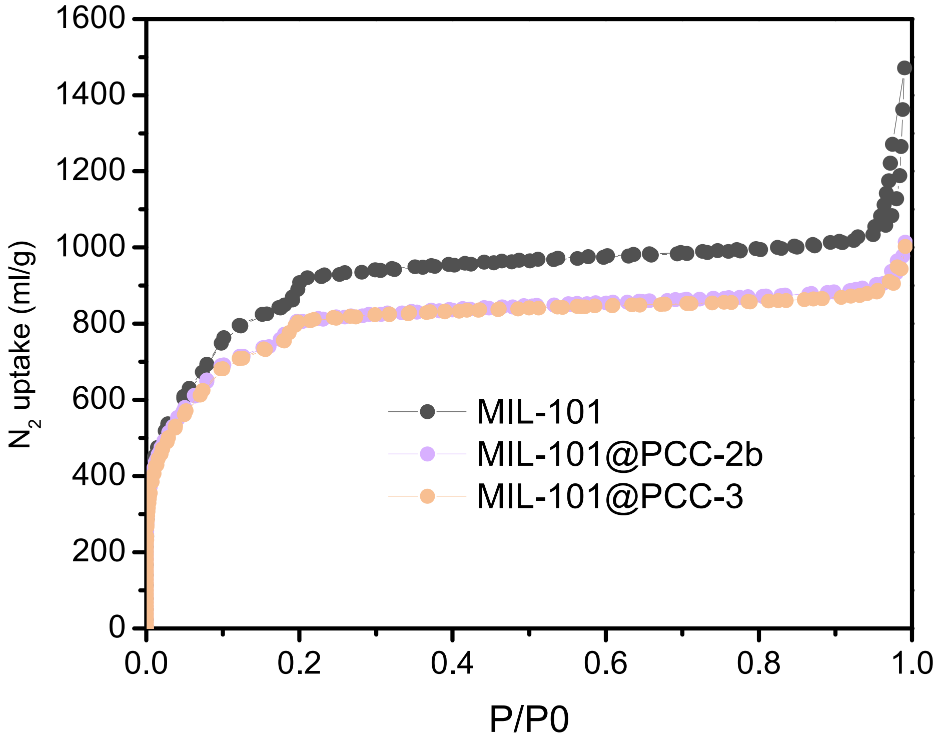

Supplementary Figure 51. Nitrogen adsorption and desorption of **MIL-101**, **MIL-101@PCC-2b**, and **MIL-222@PCC-3**.

Supplementary Table 9. ICP-OES data of **MIL-101@PCCs**.

	V_0（mL)	C_{Cr} (mg/L)	C_{Co} (mg/L)	C_{Pd} (mg/L)	PCC/MIL-101@PCC (wt%)
MIL-101@PCC-4	10	9.93	1.73	/	23.49
MIL-101 mixed with PCC-4	10	13.16	0.10	/	1.32
MIL-101@PCC-2b	10	12.53	0.42	/	5.06
MIL-101@PCC-5	10	9.79	/	0.53	8.22
MIL-101 mixed with PCC-5	10	9.03	/	0.02	0.37
MIL-101@PCC-3	10	12.95	/	0.12	1.14

The molecular formula of MIL-101 is $C_{136}Cr_{17}O_{91}$, and the relative molecular weight is 3968.53, in which Zr accounts for 22.27% of the relative molecular weight.

The molecular formula for the PCC-4 is $C_{336}H_{144}S_{48}N_{24}O_{198}Co_{24}Na_{24}$, the relative molecular weight is 11190.05, in which Co accounts for 12.64% of the relative molecular weight.

The molecular formula for the PCC-5 is $Pd_6N_{48}C_{132}H_{108}P_{12}F_{72}$, the relative molecular weight is 4744.68, in which Pd accounts for 13.46% of the relative molecular weight.

The molecular formula for the PCC-2b is $C_{432}H_{360}S_{24}N_{24}O_{126}Co_{24}$, the relative molecular weight is 10087.52, in which Co accounts for 14.02% of the relative molecular weight.

The molecular formula for the PCC-3 is $Pd_6C_{132}H_{96}N_{48}O_{36}$, the relative molecular weight is 3569.00, in which Pd accounts for 17.89% of the relative molecular weight.

In MIL-101@PCC-4, the concentration of Cr is 9.93 mg/L, Co is 1.73 mg/L, and the constant volume is 10 ml.

M_{Cr}=9.93×10×10^{-3}=0.0993 mg, $M_{MIL-101}$=0.0993/0.2227≈0.44589 mg;

M_{Co}=1.73×10×10^{-3}=0.0173 mg, M_{PCC-4}=0.0173/0.1264≈0.13687 mg;

$M_{MIL-101@PCC-4}$=0.44589+0.13687=0.58276 mg;

The mass proportion of PCC-4 in MIL-101@PCC-4 is 0.13687/0.58276≈ 23.49%.

In MIL-101 mixed with PCC-4, the concentration of Cr is 13.16 mg/L, Co is 0.10 mg/L, and the constant volume is 10 ml.

M_{Cr}=13.16×10×10^{-3}=0.1316 mg, $M_{MIL-101}$=0.1316/0.2227≈0.59093 mg;

M_{Co}=0.10×10×10^{-3}=0.0010 mg, M_{PCC-4}=0.0010/0.1264≈0.00791 mg;

$M_{MIL-101\ mix\ with\ PCC-4}$=0.59093+ 0.00791=0.59884 mg;

The mass proportion of PCC-4 in MIL-101@PCC-4 is 0.00791/0.59884≈ 1.32%.

.

In MIL-101@PCC-2b, the concentration of Cr is 12.53 mg/L, Co is 0.42 mg/L, and the constant volume is 10 ml.

M_{Cr}=12.53×10×10^{-3}=0.1253 mg, $M_{MIL-101}$=0.1253/0.2227≈0.56264 mg;

M_{Co}=0.42×10×10^{-3}=0.0042 mg, M_{PCC-2b}=0.0042/0.1402≈0.02996 mg;

$M_{MIL-101@PCC-2b}$=0.56264+0.02996=0.59260 mg;

The mass proportion of PCC-4 in MIL-101@PCC-2b is 0.02996/0.59260≈ 5.06%.

In MIL-101@PCC-5, the concentration of Cr is 9.79 mg/L, Pd is 0.53 mg/L, and the constant volume is 10 ml.

M_{Cr}=9.79×10×10^{-3}=0.0979 mg, $M_{MIL-101}$=0.0979/0.2227≈0.43960 mg;

M_{Pd}=0.53×10×10^{-3}=0.0053 mg, M_{PCC-5}=0.0053/0.1346≈0.03938 mg;

$M_{MIL\text{-}101@PCC\text{-}5}$=0.43960+0.03938=0.47898 mg;

The mass proportion of PCC-5 in MIL-101@PCC-5 is 0.03938/0.47898≈8.22 %

In MIL-101 mixed with PCC-5, the concentration of Cr is 9.03 mg/L, Pd is 0.02 mg/L, and the constant volume is 10 ml.

$M_{Cr}=9.03\times10\times10^{-3}$=0.0903 mg, $M_{MIL\text{-}101}$=0.0903/0.2227≈0.40548 mg

$M_{Pd}=0.02\times10\times10^{-3}$=0.0002 mg, $M_{PCC\text{-}5}$=0.0002/0.1346≈0.00149 mg

$M_{MIL\text{-}101 \text{ mix with } PCC\text{-}5}$=0.00149+0.40548=0.40697 mg

The mass proportion of PCC-5 in MIL-101 mix with PCC-5 is 0.00149/0.40697≈0.37 %

In MIL-101@PCC-3, the concentration of Cr is 12.95 mg/L, Pd is 0.12 mg/L, and the constant volume is 10 ml.

$M_{Cr}=12.95\times10\times10^{-3}$=0.1295 mg, $M_{MIL\text{-}101}$=0.1295/0.2227≈0.58150 mg;

$M_{Pd}=0.12\times10\times10^{-3}$=0.0012 mg, $M_{PCC\text{-}3}$=0.0012/0.1789≈0.00671 mg;

$M_{MIL\text{-}101@PCC\text{-}3}$=0.00671+0.58150=0.58821 mg;

The mass proportion of PCC-5 in MIL-101@PCC-3 is 0.00671/0.58821≈1.14 %

(a)

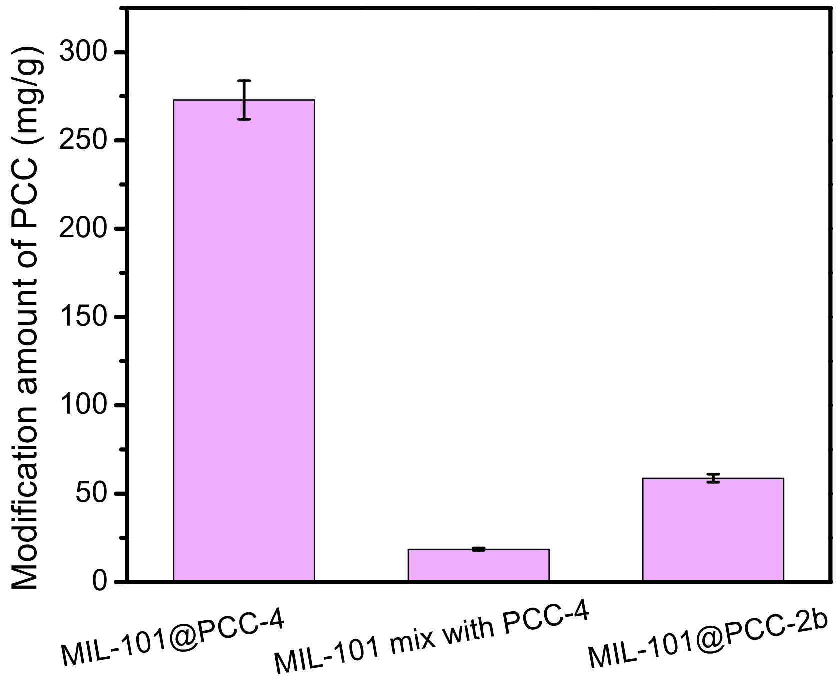

(b)

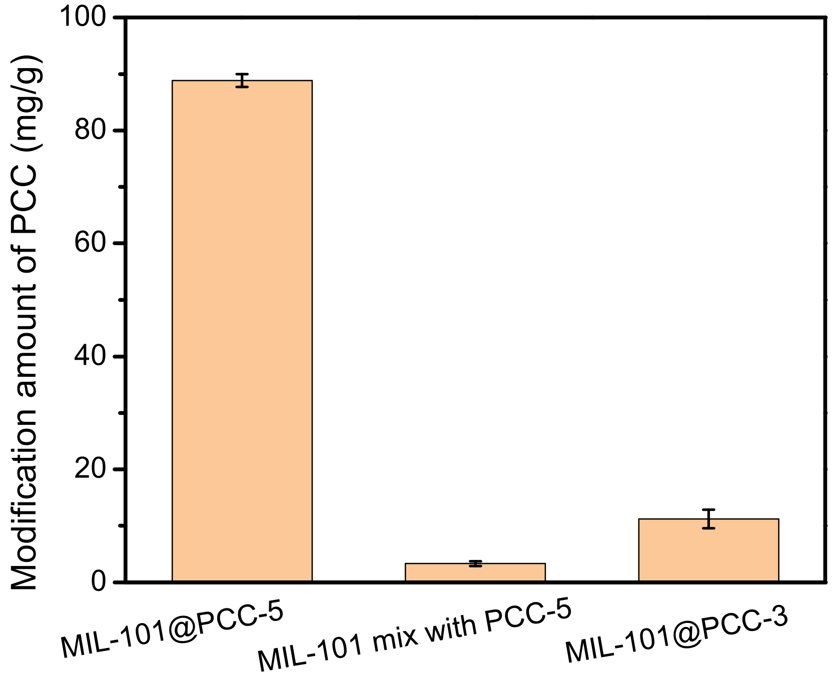

Supplementary Figure 52. The loading amount of **PCCs** in different **MIL-101** composites. Data are presented as the mean ± SD.

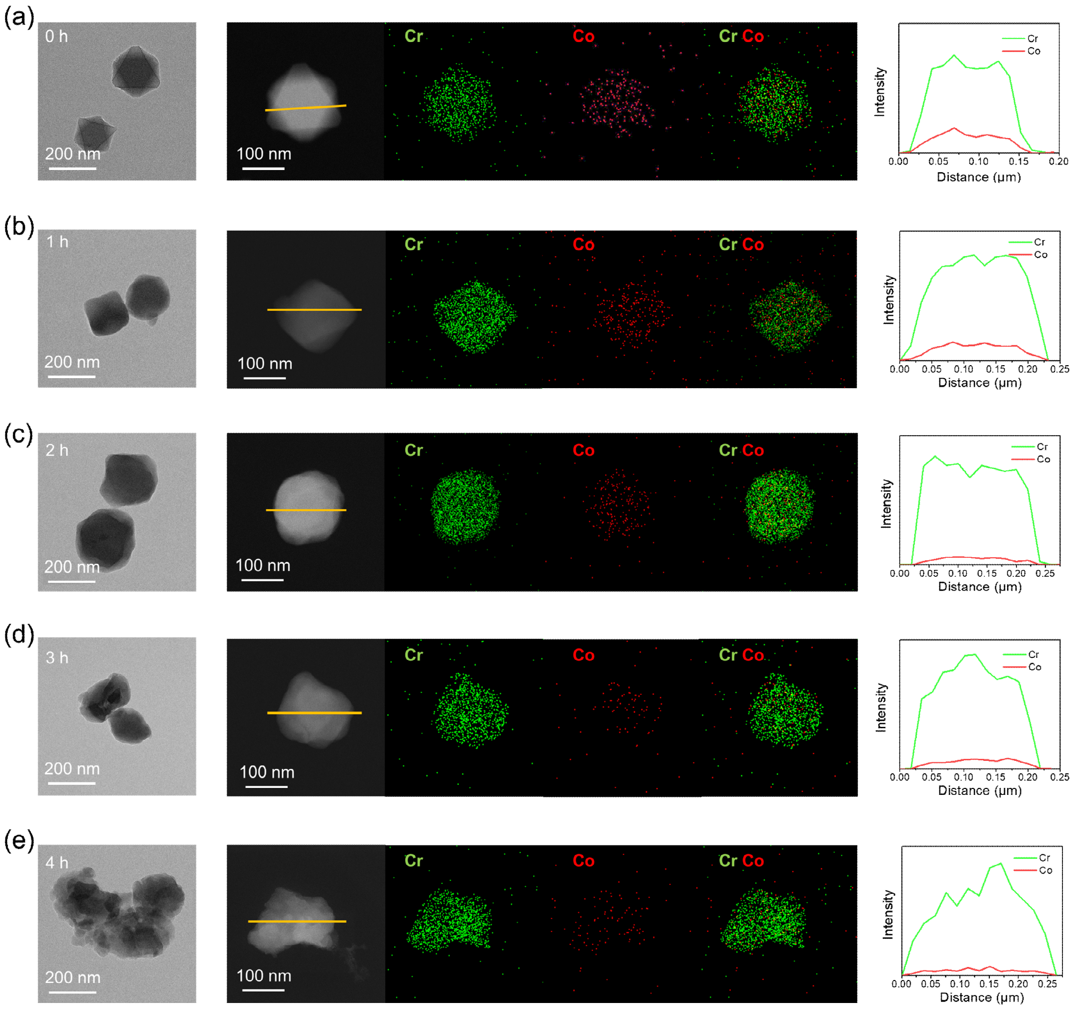

Supplementary Figure 53. STEM images, elemental mapping and linear scanning analysis of **MIL-101@PCC-4** particles following various durations of ball-milling. (a) 0 h, (b) 1 h, (c) 2 h, (d) 3 h, and (e) 4 h.

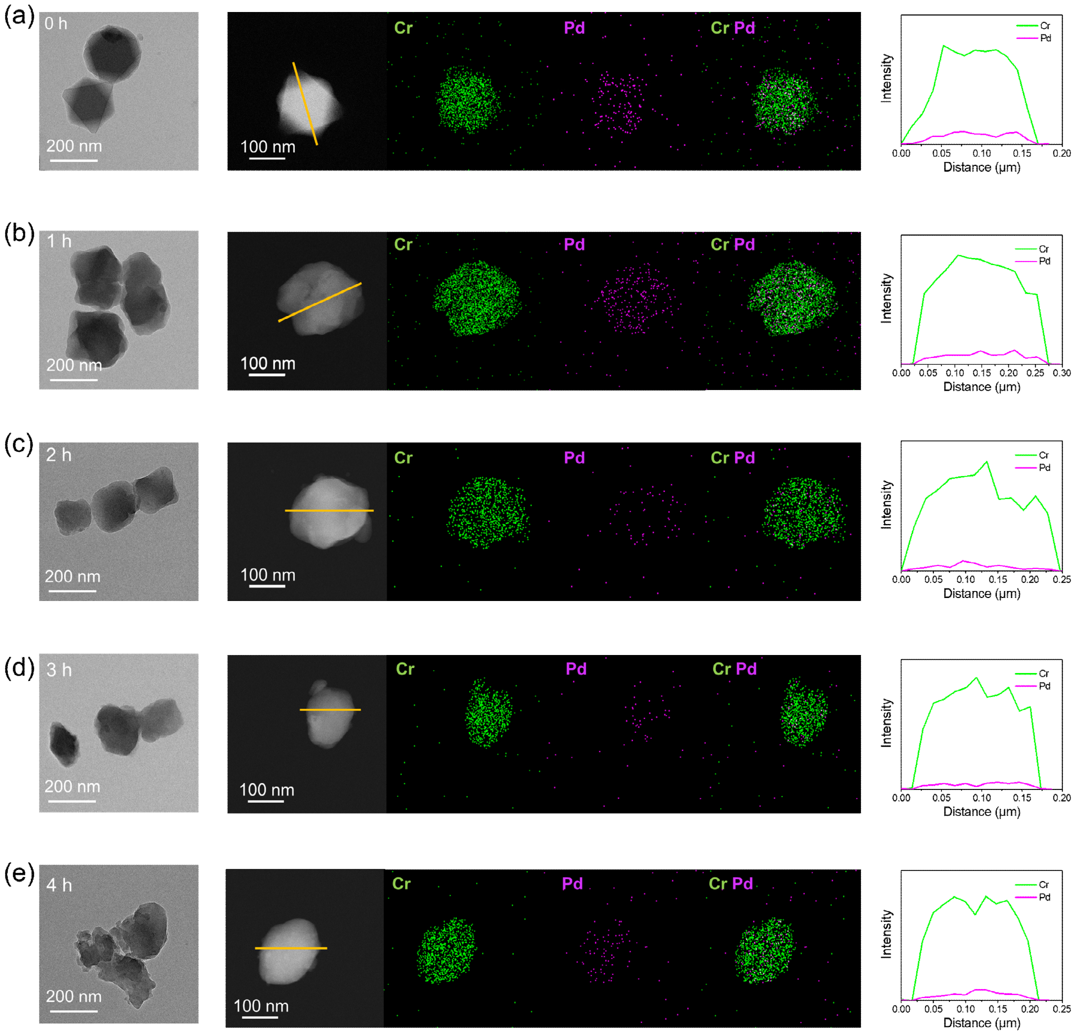

Supplementary Figure 54. STEM images, elemental mapping and linear scanning analysis of **MIL-101@PCC-5** particles following various durations of ball-milling. (a) 0 h, (b) 1 h, (c) 2 h, (d) 3 h, and (e) 4 h.

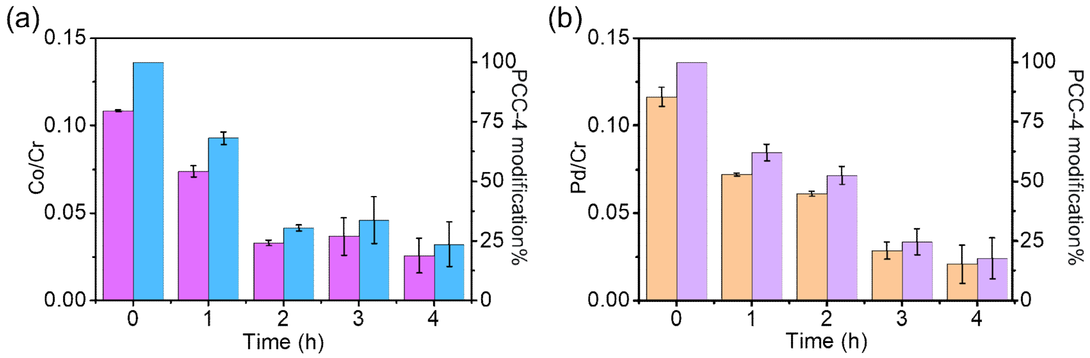

Supplementary Figure 55. Quantitative analysis of (a) the Co/Cr ratios and **PCC-4** modification percentage of **MIL-101@PCC-4** as well as (b) the Pd/Cr ratios and **PCC-5** modification percentage of **MIL-101@PCC-5** under ball-milling over time. Data are presented as the mean ± SD.

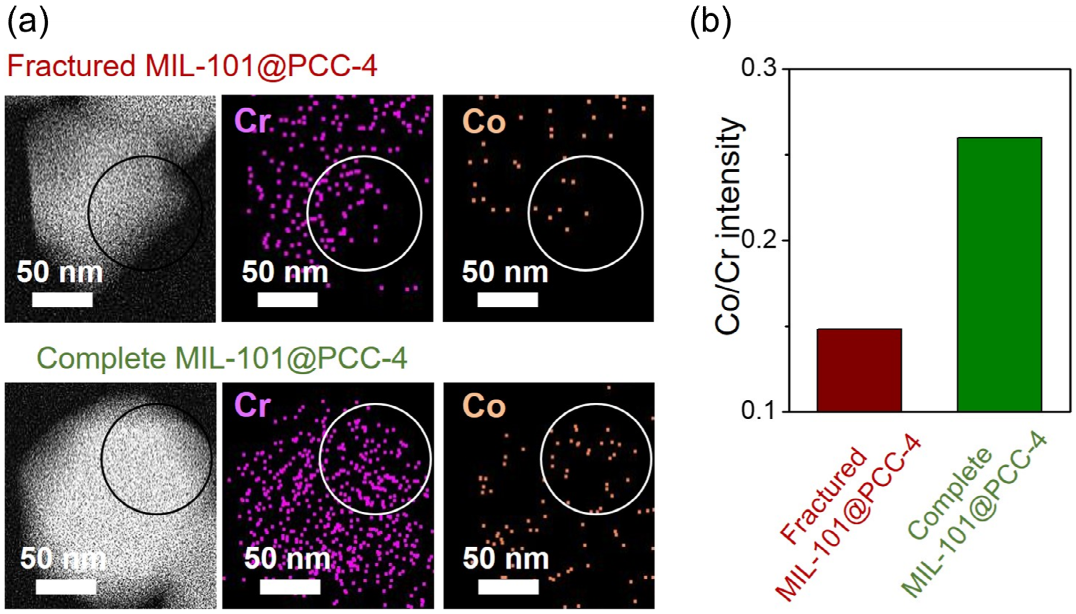

Supplementary Figure 56. (a)The EDX element mapping of **MIL-101@PCC-4** particles before and after fracturing the particle using probe sonicator (i). The comparison of the Co/Cr intensity ratios of the fractured and complete MIL-101@PCC-4 particles (j).

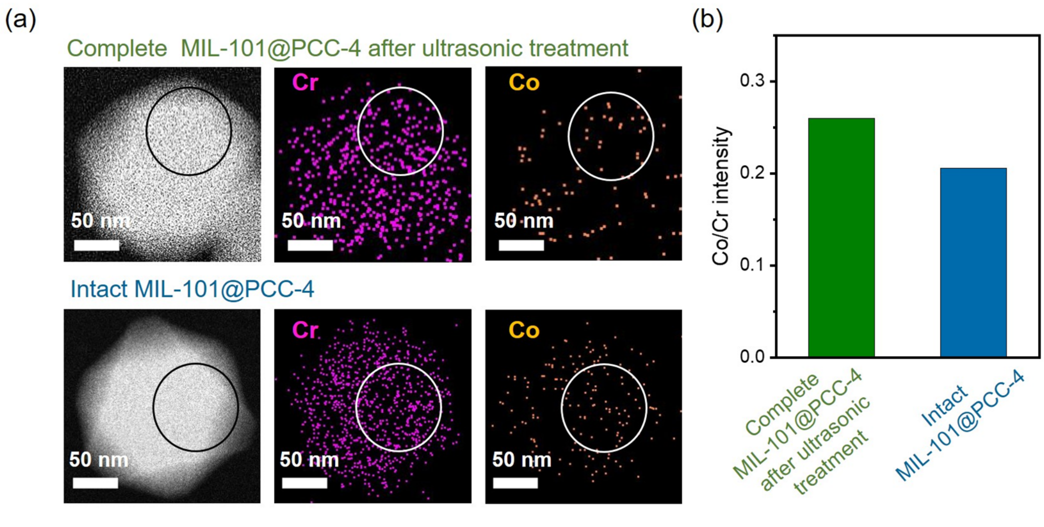

Supplementary Figure 57. (a) The EDX element mapping of complete **MIL-101@PCC-4** samples with and without probe sonication treatment. (b) Comparison of the Co/Cr ratio of **MIL-101@PCC-4** samples with and without probe sonication treatment (b).

Section 8. Theoretical Calculations of MIL-101@PCCs

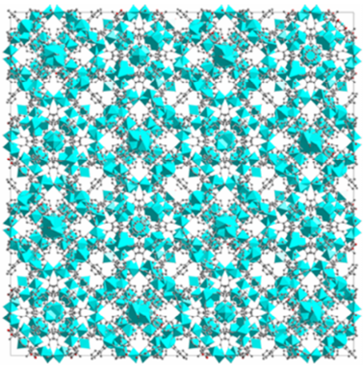

Cell of MIL-101

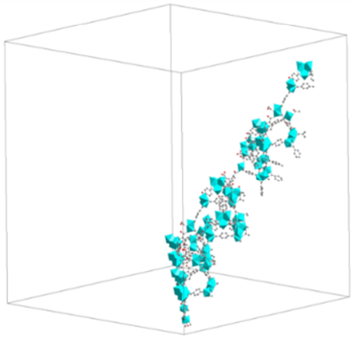

{111} of MIL-101

The cell parameters of MIL-101 (Cr_{840} O_{9100} C_{6948}) are as follows:
a=b=c=88.869Å
α=β=γ=90°
V=701860.31 $Å^3$=701.86031 nm^3

Suppose MIL-101 is a regular octahedron with edges of 200 nm.
The volume of MIL-101 is =3770666.7 nm^3
MIL-101 has a total Cr_3 cluster =3770666.7×840/3/701.86031≈1504269.0
MIL-101 has a surface area of 138560 nm^2

The area of {111} crystal surface of MIL-101 cell is 8.8869×√2/2×√3×8.8869×√2≈ 15.39 nm^2. And there are 72 Cr atoms on the plane of {111} crystal surface, namely 24 Cr_3 clusters, so the Cr_3 cluster on the surface of MIL-101 =138560/15.39×24≈216078.

According to the ICP-OES:

	Cr (mg)	Co (mg)	Pd (mg)
MIL-101@PCC-4	0.0993	0.0173	/
MIL-101@PCC-5	0.0979	/	0.0053

The number of Cr_3 clusters and PCC can be calculated as follows:

	Cr_3	PCC-4	PCC-5
MIL-101@PCC-4	98.11	1.89	/
MIL-101@PCC-5	98.69	/	1.31

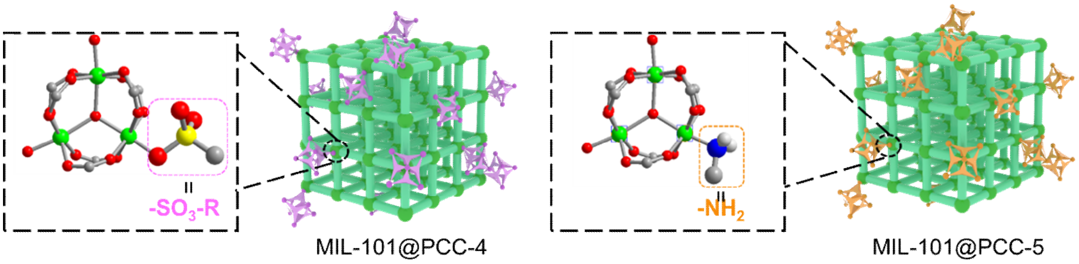

Each MIL-101 nanoparticle can support 28,430.68 PCC-4 or 19,705.92 PCC-5.

According to the ICP experiment, PCC-4: the surface Cr_3 cluster is ≈13.3%. PCC-5: the surface Cr_3 cluster ≈ 9.1%

In the material MIL-101@PCC, the amount of PCC modification was about 10% of the number of Cr_3 clusters on the surface, which suggested that PCCs were mainly distributed on the surface of MIL-101.

Section 9. Dye adsorptions of MIL-101@PCCs

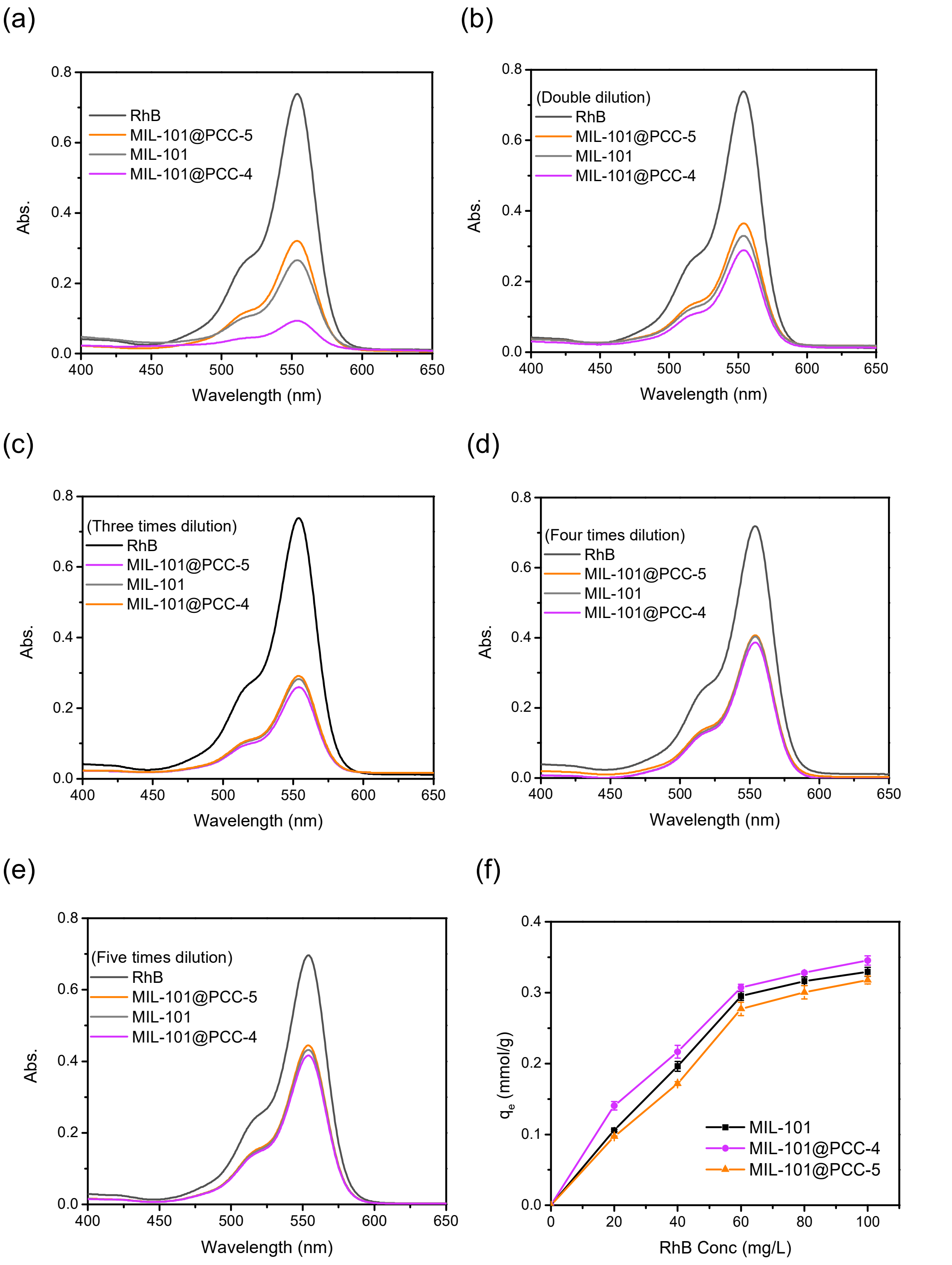

Supplementary Figure 58. The saturated adsorption capacity of **MIL-101** and **MIL-101@PCCs** for rhodamine B at 20 mg/L (a), 40 mg/L (b), 60 mg/L (c), 80 mg/L (d), 100 mg/L (e), and the saturation adsorption curves at different concentrations. Data are presented as the mean ± SD (f).

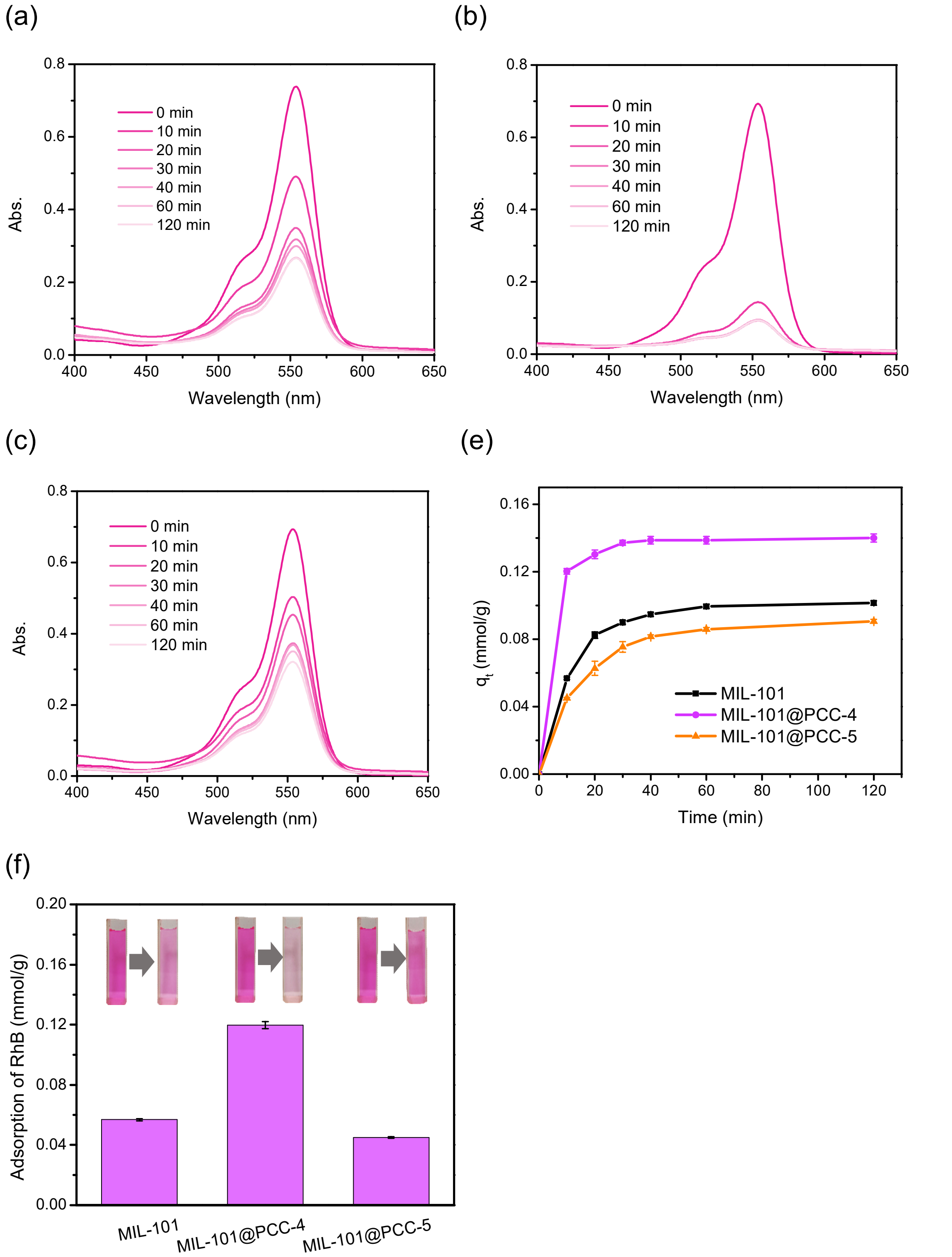

Supplementary Figure 59. The adsorption capacity of rhodamine B by **MIL-101** (a), **MIL-101@PCC-4** (b), and **MIL-101@PCC-5** (c) at different times in 20 mg/L rhodamine B aqueous solution; adsorption curves of three materials with time. Data are presented as the mean ± SD (d); the adsorption capacity of **MIL-101** and **MIL-101@PCCs** in 20 mg/L rhodamine B aqueous solution within 10 min. Data are presented as the mean ± SD (e).

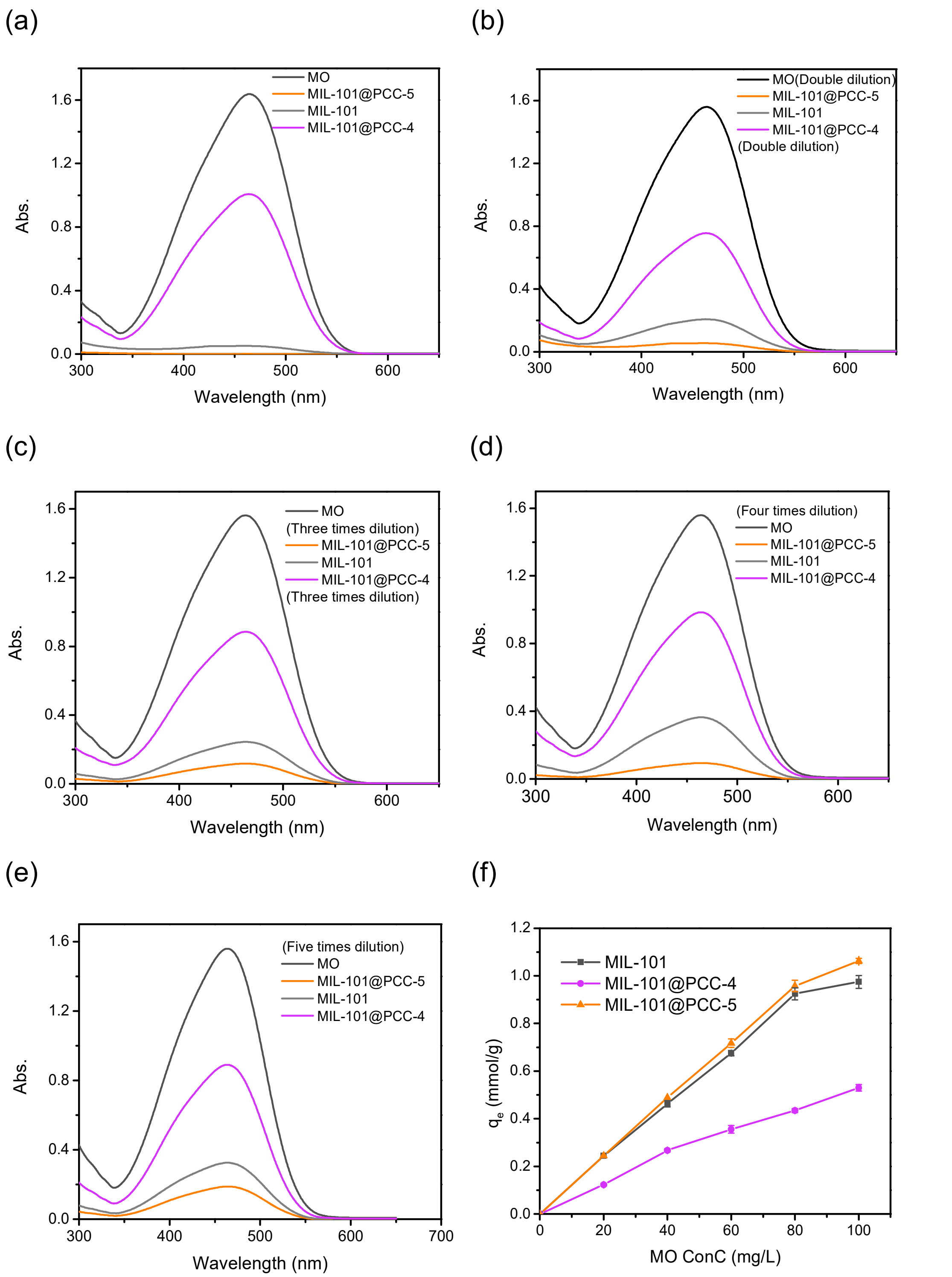

Supplementary Figure 60. The saturated adsorption capacity of **MIL-101** and **MIL-101@PCCs** for methyl orange at 20 mg/L (a), 40 mg/L (b), 60 mg/L (c), 80 mg/L (d), 100 mg/L (e), and the saturation adsorption curves at different concentrations. Data are presented as the mean ± SD (f).

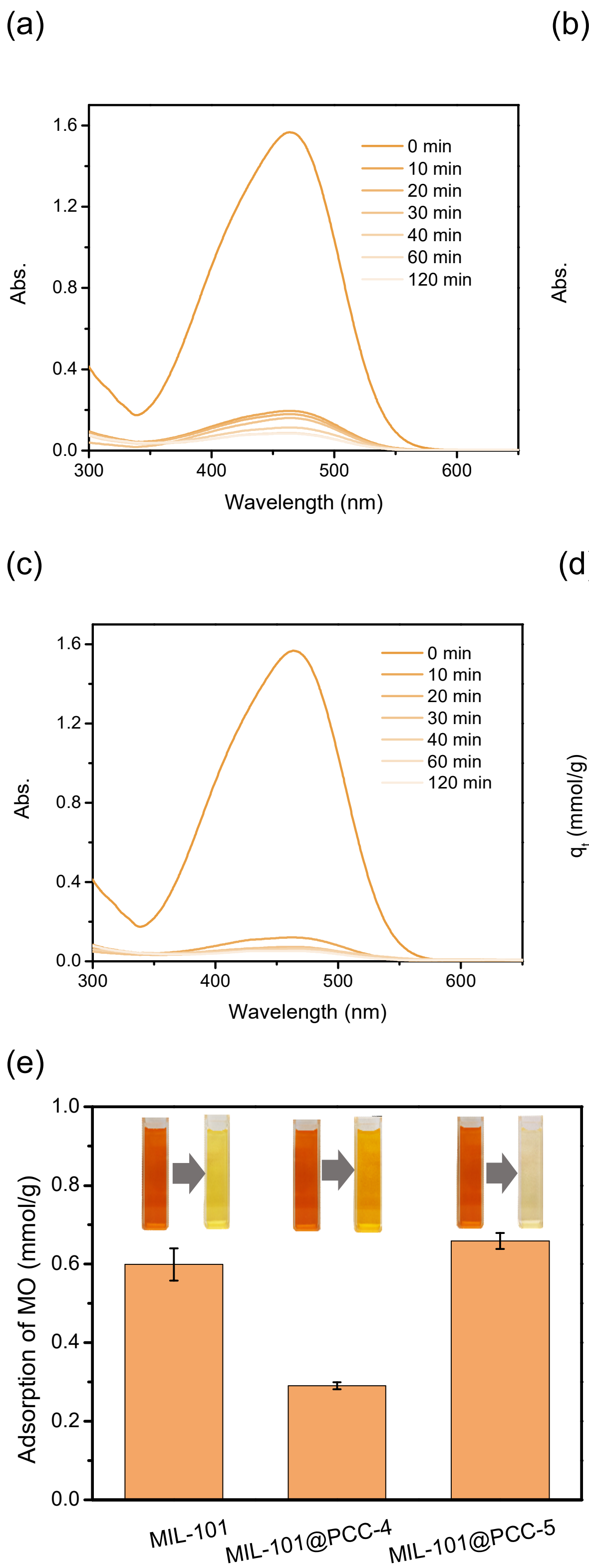

Supplementary Figure 61. The adsorption capacity of rhodamine B by **MIL-101** (a), **MIL-101@PCC-4** (b), and **MIL-101@PCC-5** (c) at different times in 60 mg/L methyl orange aqueous solution; adsorption curves of three materials with time. Data are presented as the mean ± SD (d); the adsorption capacity of **MIL-101** and **MIL-101@PCCs** in 60 mg/L methyl orange aqueous solution within 10 min. Data are presented as the mean ± SD (e).

(a)

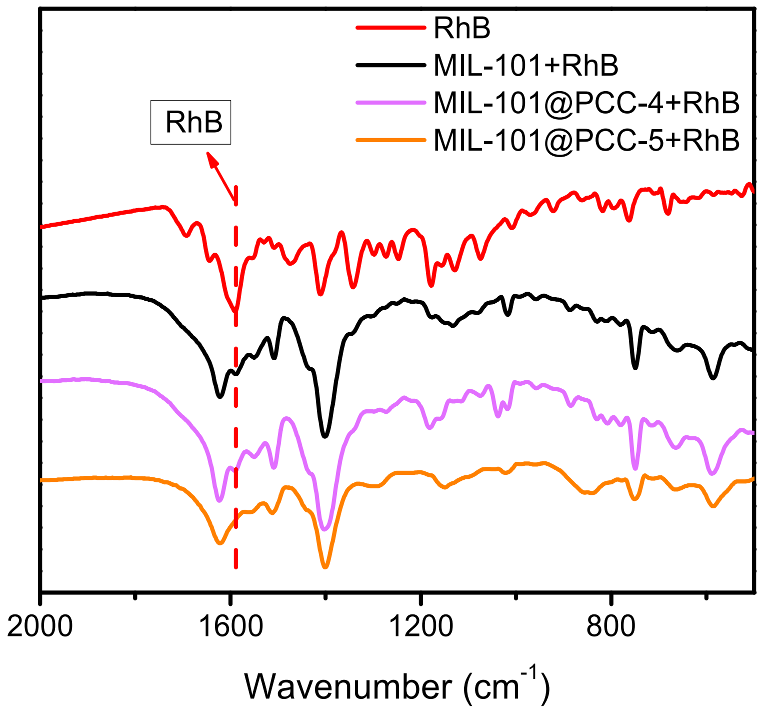

(b)

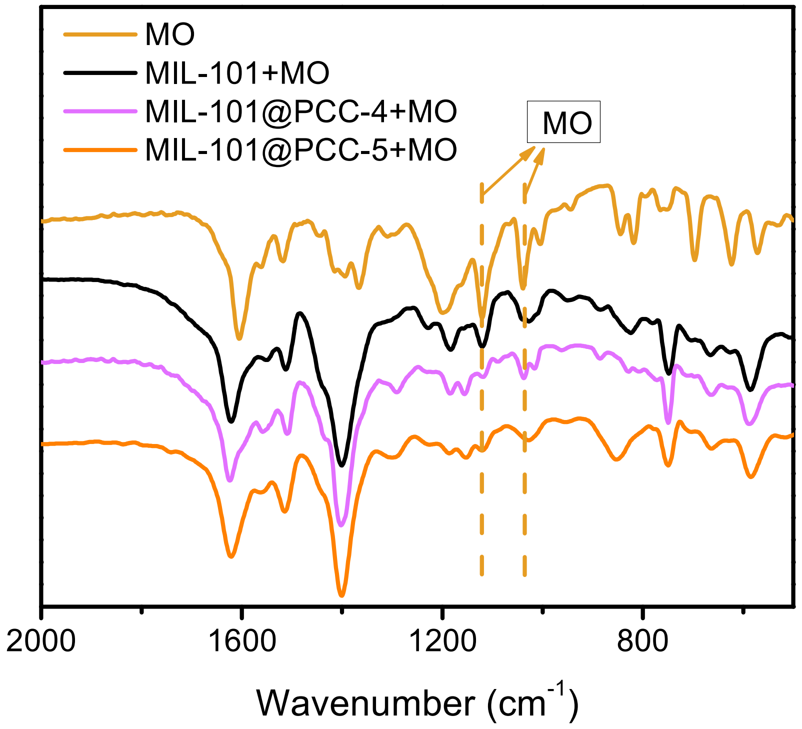

Supplementary Figure 62. The FT-IR spectra of **MIL-101@PCCs** after the dye adsorption. (a) After RhB adsorption. (b) After MO adsorption.

The experiments were conducted as follows: 5 mg of **MIL-101@PCCs** were dispersed in 40 mL of anionic dye solution containing Rhodamine B and Methylene Orange at a concentration of 40 mg/L. The **MIL-101@PCCs** with adsorbed dye were washed with a mixed solvent of MeCN and MeOH (VMeCN : VEtOH=1:1) and subjected to further analysis by UV-Vis. The release percentage of Rhodamine B (cationic, +1) for **MIL-101**, **MIL-101@PCC-4**, and **MIL-101@PCC-5** was found to be 71.4% and 81.5%, respectively. For Methyl Orange (anionic, -1), the release percentage for **MIL-101**, **MIL-101@PCC-4**, and **MIL-101@PCC-5** was 18.7%, 22.7%, and 20.2%, respectively. **MIL-101** alone was not able to release all the dyes due to the high surface area and porosity, which resulted in an interaction between the adsorbed dye molecules and the pores' surface, retaining a portion of the encapsulated molecules.

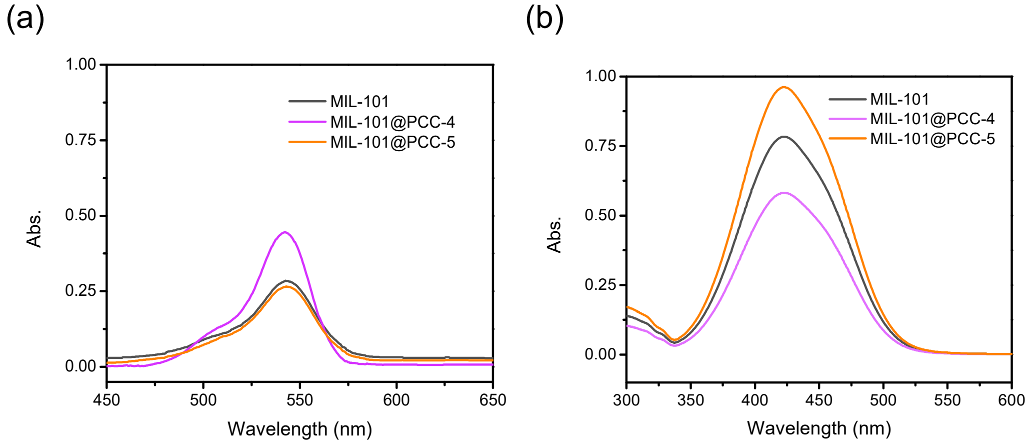

Supplementary Figure 63. UV-vis spectra of dye molecules released from **MIL-101@PCCs**. (a) RhB. (b) MO.

Supplementary Table 10. Dye release from **MIL-101@PCCs**.

**Materials**	**Adsorption capacity of RhB (mg)**	**The release of RhB (mg)**	**Release rate of RhB (%)**
MIL-101	0.42	0.30	71.4
MIL-101@PCC-4	0.54	0.44	81.5
MIL-101@PCC-5	0.41	0.29	70.7
**Materials**	**Adsorption capacity of MO (mg)**	**The release of MO (mg)**	**Release rate of MO (%)**
MIL-101	0.75	0.14	18.7
MIL-101@PCC-4	0.44	0.10	22.7
MIL-101@PCC-5	0.79	0.16	20.2

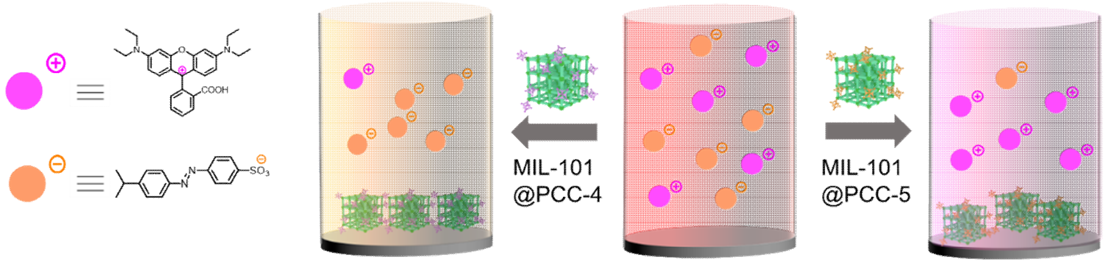

Supplementary Figure 64. Schematic diagram of the **MIL-101@PCCs** adsorbing the mixed dye of Rhodamine B and Methyl Orange.

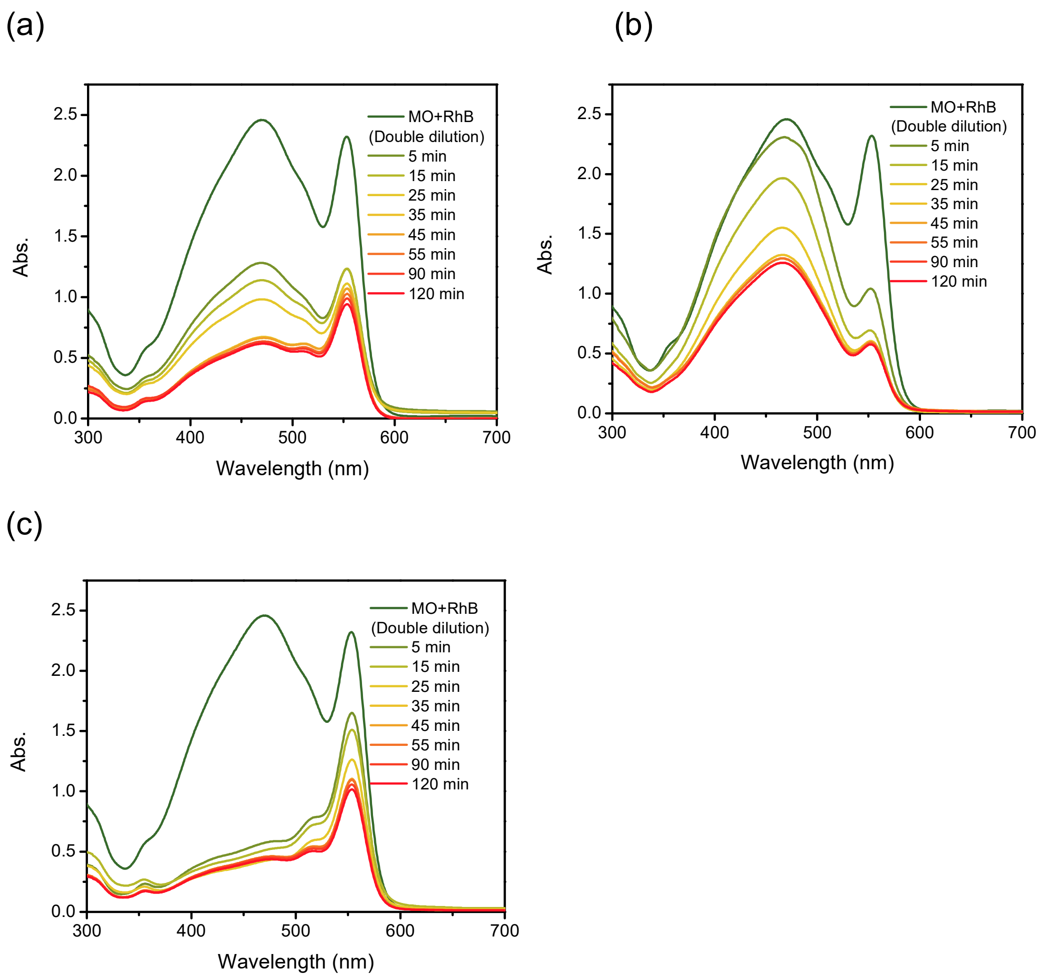

Supplementary Figure 65. UV-vis for measuring the adsorption behavior of **MIL-101** (a), **MIL-101@PCC-4** (b), and **MIL-101@PCC-5** (c) in the mixed dye solution.

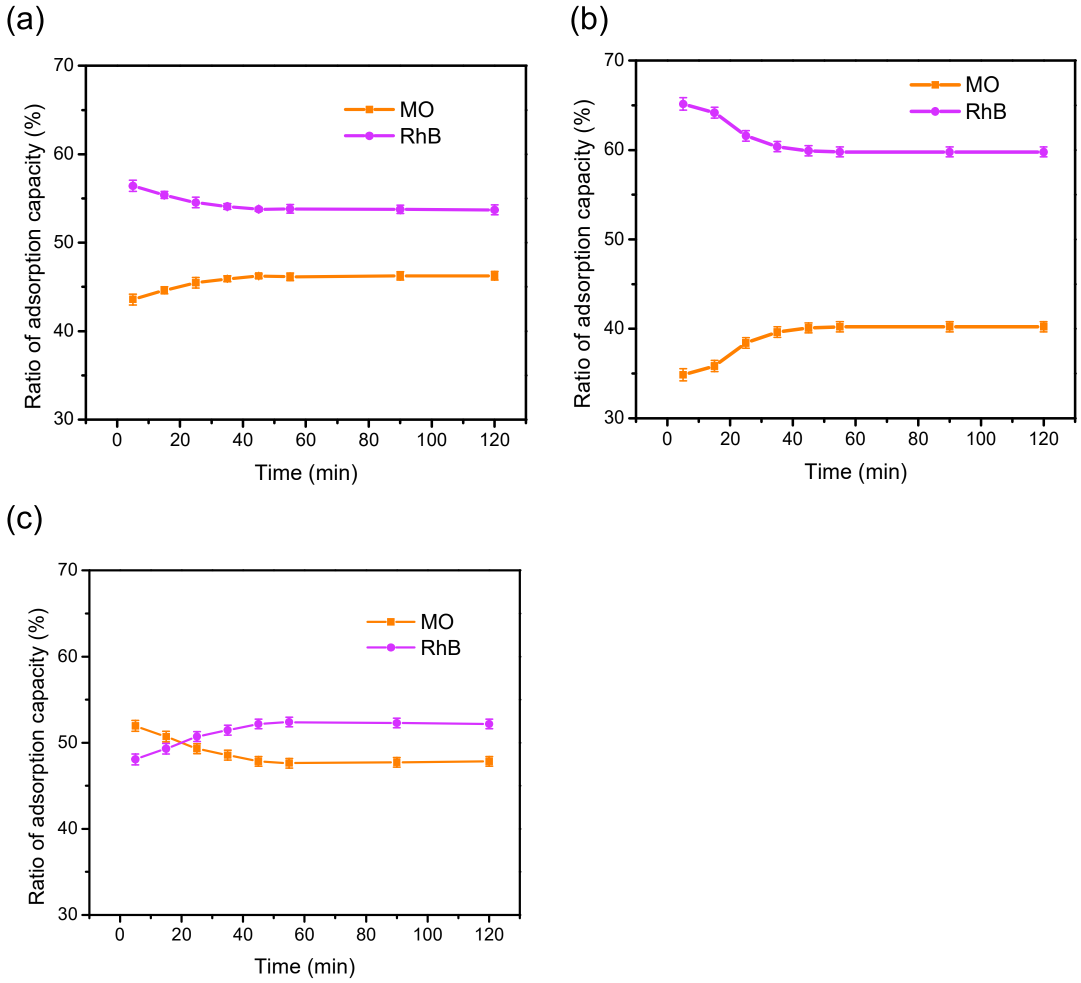

Supplementary Figure 66. Adsorption behavior of Rhodamine B and methyl orange mixed dyes by **MIL-101** (a), **MIL-101@PCC-4** (b), **MIL-101@PCC-5** (c). Data are presented as the mean ± SD.

Supplementary Table 11. The dye adsorption capacity of **MIL-101** and **MIL-101@PCCs** in the first 5 min.

	The total amount of dye adsorbed (mg/g)	The amount of MO (mg/g)	The amount of RhB (mg/g)
MIL-101	526.1	180.5	345.6
MIL-101@PCC-4	508.0	134.7	373.3
MIL-101@PCC-5	512.3	213.6	298.7

Section 10. Catalytic reactions of MIL-101@PCCs

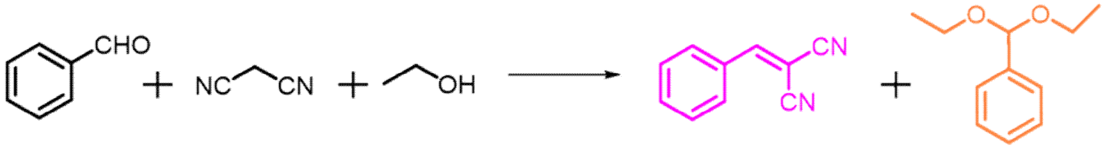

A B C D E

Supplementary Table 12. Catalytic activity and selectivity of **MIL-101** modified **PCC** components.

Entries	Catalyst	Conv. (%)	Yield (%)	
			D	E
1	blank	18.8	/	18.8
2	PCC-4	44.7	44.7	/
3	PCC-5	46.4	7.8	38.6
	MIL-101	39.5	39.5	/
4	MIL-101@PCC-4	64.9	64.9	/
5	MIL-101@PCC-5	70.3	8.2	62.1
6	MIL-101@TSSC	54.2	/	54.2
7	MIL-101@TATB	41.3	/	41.3
8	MIL-101@$CoCl_2$	45.5	/	45.5
9	MIL-101@NH_2-Bipyridine	40.6	2.5	38.1
10	MIL-101@TPT	59.9	13.6	46.3
11	MIL-101@$PdCl_2$	84.7	80.8	3.9
12	MIL-101@PCC-2b	42.2	42.2	/
13	MIL-101@PCC-3	40.6	40.6	/
14	MIL-101 mixed with PCC-4	70.4	/	70.4
15	MIL-101 mixed with PCC-5	40.3	/	40.3

Reaction condition: benzaldehyde (1.0 mmol), malononitrile (1.1 mmol), EtOH: DMSO =4:1 (20 mL), 20 mg catalyst, 25 °C for 2 h.

(a)

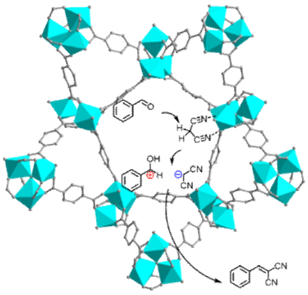

(b)

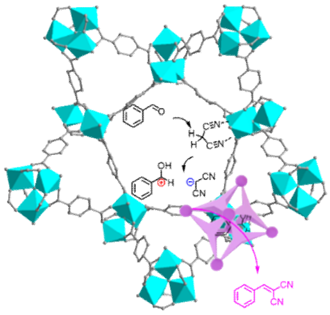

(c)

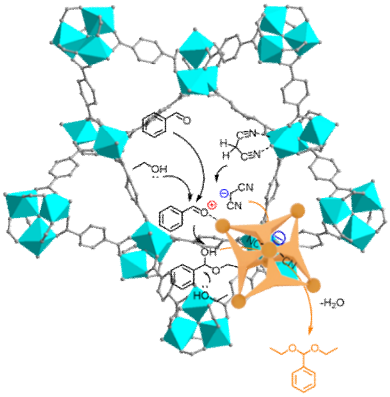

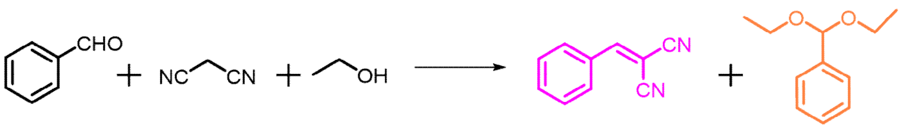

A B C D E

Supplementary Figure 67. Diagram of the catalytic reaction mechanism of **MIL-101** (a), **MIL-101@PCC-4** (b), **MIL-101@PCC-5** (c).

(a)

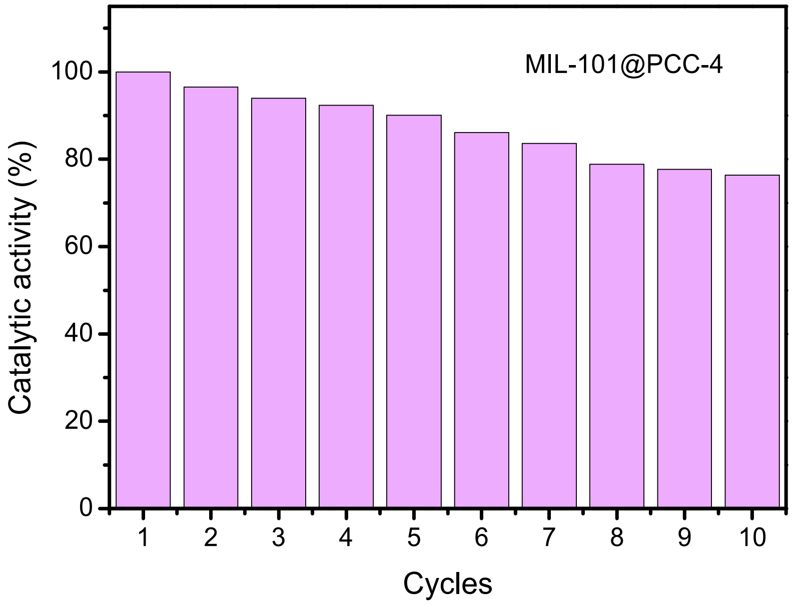

(b)

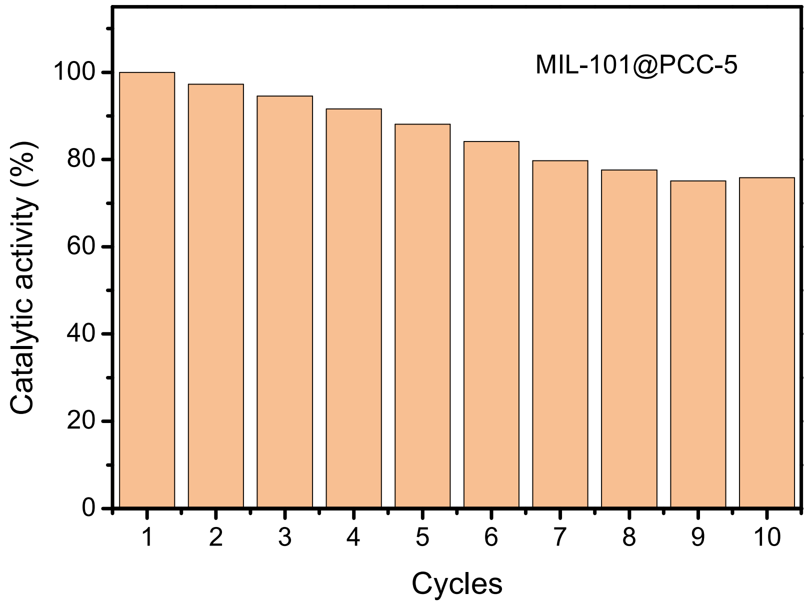

Supplementary Figure 68. The catalytic activity of **MIL-101@PCC-4** (a) and **MIL-101@PCC-5** (b) after 10 times of cycles.

(a)

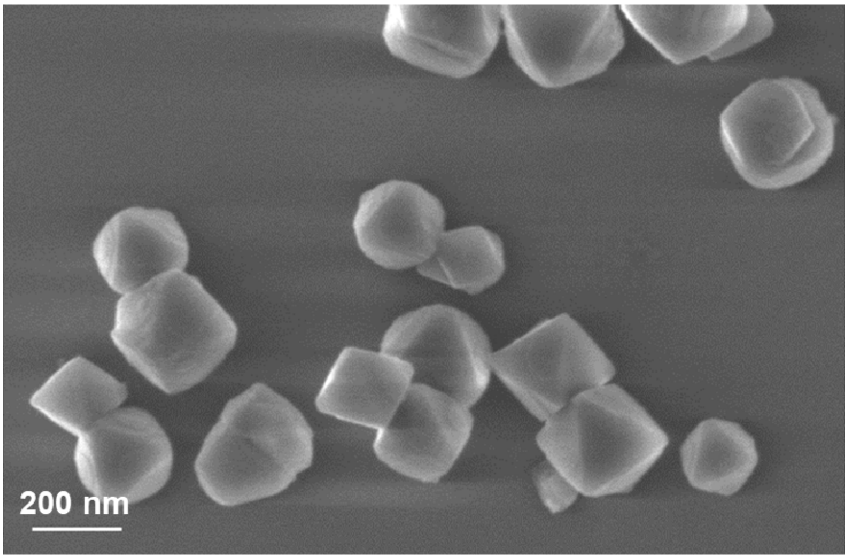

(b)

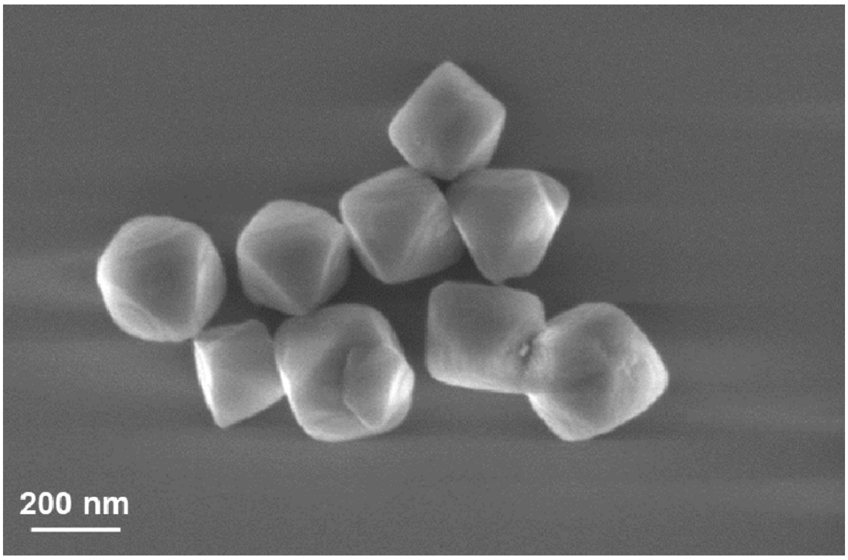

Supplementary Figure 69. SEM images of **MIL-101@PCC-4** (a) and **MIL-101@PCC-5** (b) after 10 times of cycles. Scale bar = 200 nm.

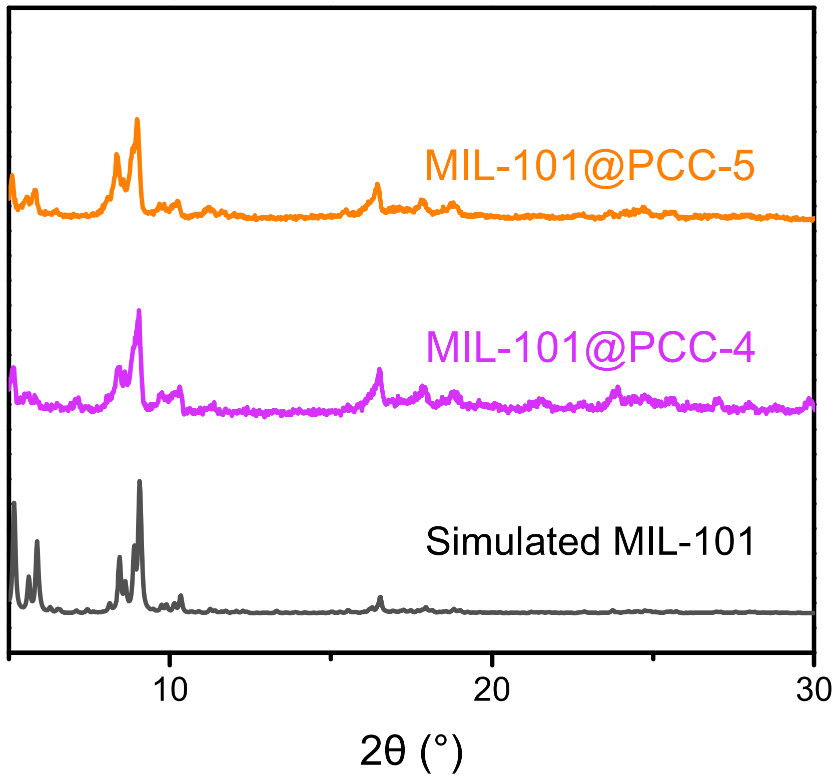

Supplementary Figure 70. PXRD of **MIL-101@PCC-4** and **MIL-101@PCC-5** after 10 times of cycles.

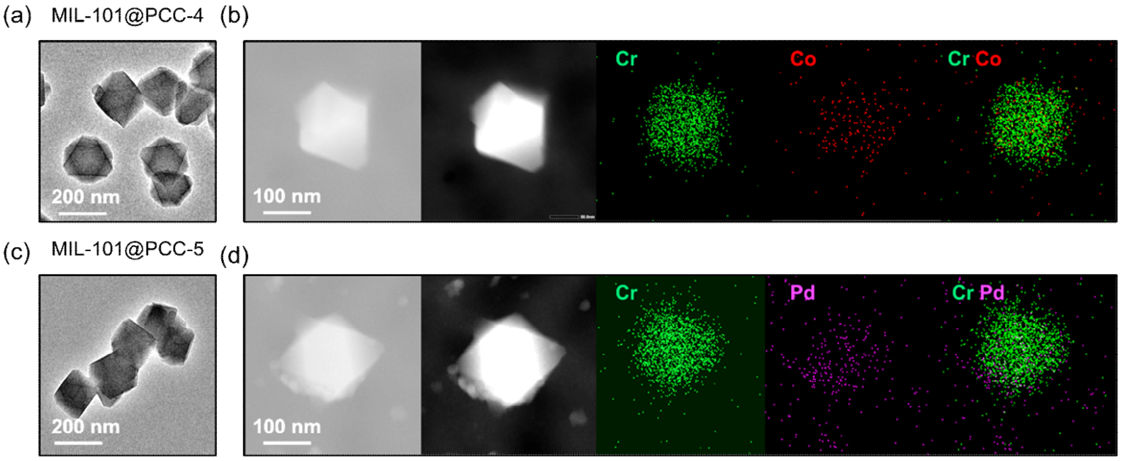

Supplementary Figure 71. The STEM images and EDX of **MIL-101@PCCs** after 10 times of cycles.

Supplementary Table 13. ICP-OES data of **MIL-101@PCCs** after 10 cycles.

	V_0 （mL)	C_{Cr} (mg/L)	C_{Co} (mg/L)	C_{Pd} (mg/L)	PCC/MIL-101@PCC (wt%)	The amount of PCC loss （wt%）
MIL-101@PCC-4	10	9.42	1.28	/	16.73	17.8
MIL-101@PCC-5	10	10.01	/	0.47	7.21	12.3

The surface etching procedure involved soaking **MIL-101@PCCs** in a 1 M aqueous hydrochloric acid solution for 2 hours with stirring. The resulting solids were collected by centrifugation, and the supernatant was washed repeatedly with deionized water until neutral. Next, the solid was soaked in DMF for 12 hours, collected by centrifugation, and washed twice with DMF to obtain **MIL-101**. Subsequently, the synthesis of **MIL-101@PCCs** was carried out according to the previously described procedure.

(a)

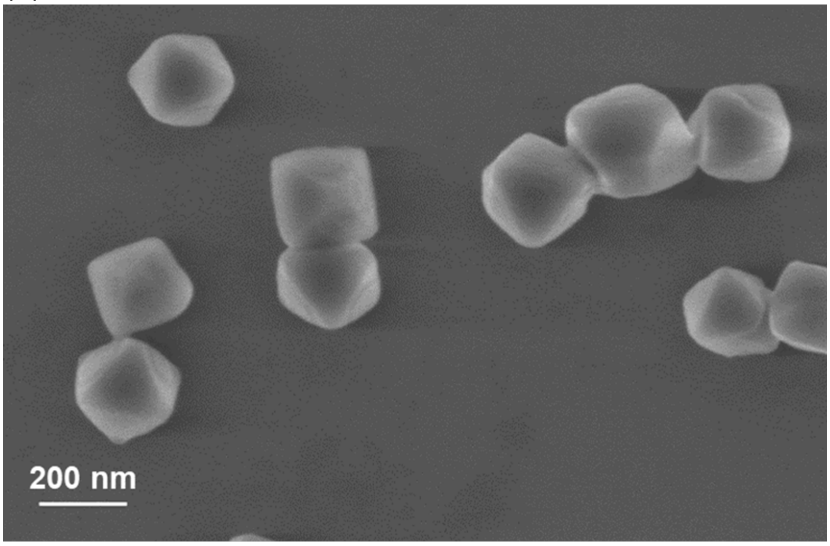

(b)

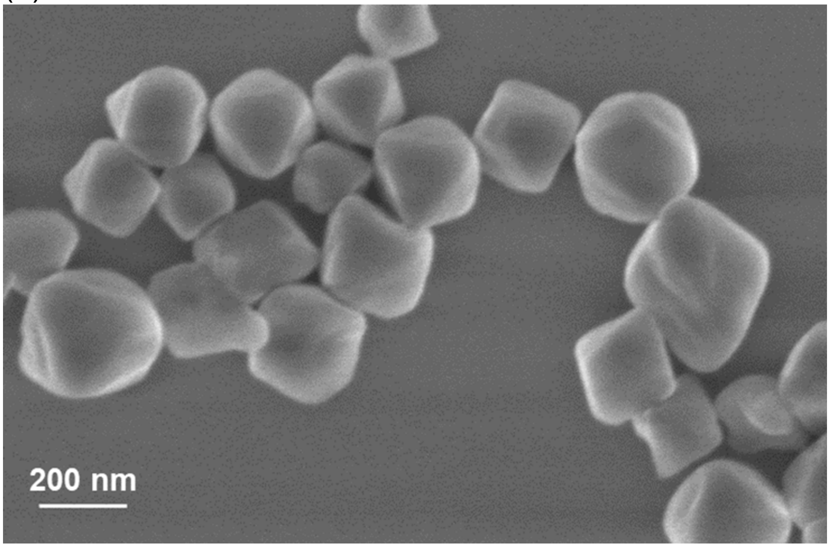

Supplementary Figure 72. SEM of **MIL-101@PCC-4** (a) and **MIL-101@PCC-5** (b) after reproducible modification. (Scale Bar: 200 nm).

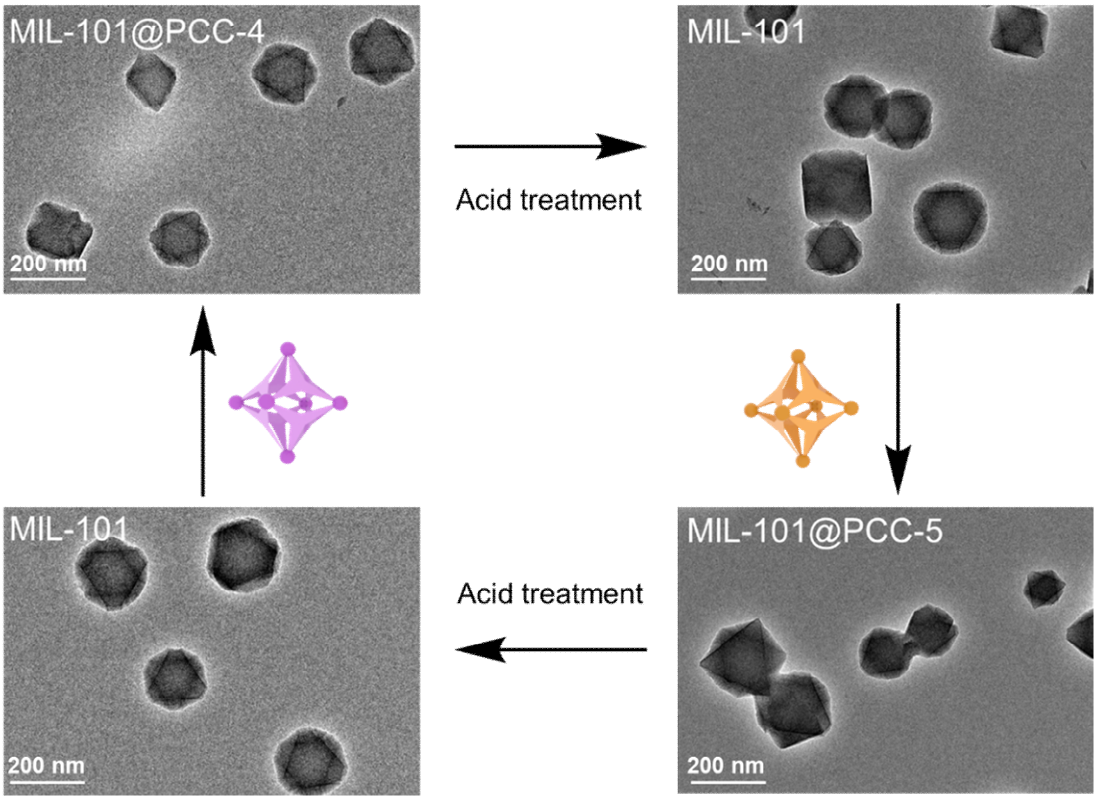

Supplementary Figure 73.TEM images of **MIL-101@PCCs** after reproducible modification.

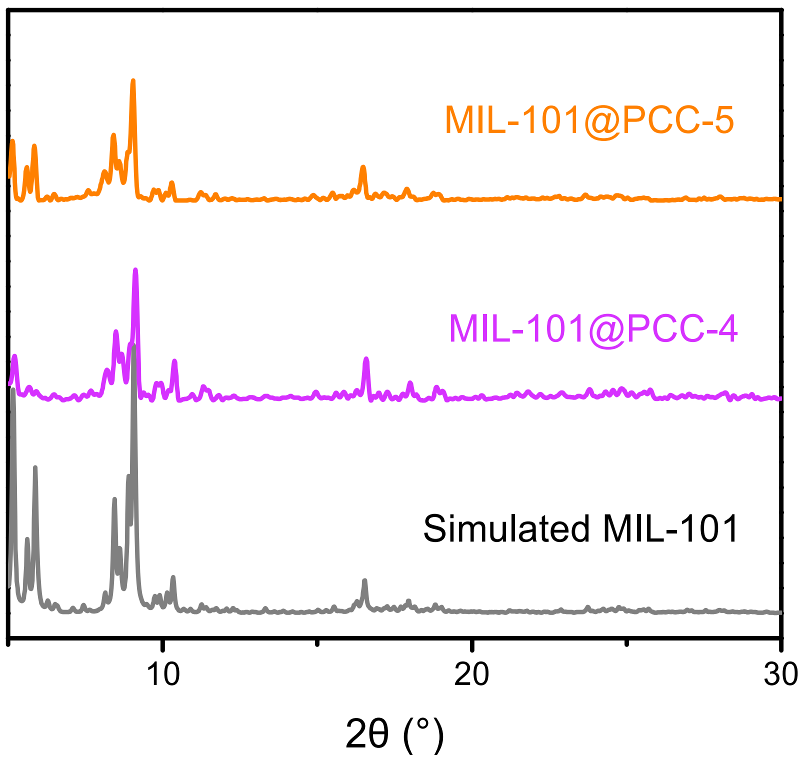

Supplementary Figure 74. PXRD of **MIL-101@PCCs** after reproducible modification.

Supplementary Table 14. ICP-OES data of **MIL-101@PCCs** after reproducible modification.

	V_0 (mL)	C_{Zr} (mg/L)	C_{Co} (mg/L)	C_{Pd} (mg/L)	Co/Cr	Pd/Cr
MIL-101@PCC-4	10	9.93	1.73	/	0.174	/
Surface etched MIL-101@PCC-4	10	14.84	0.12	/	0.009	/
MIL-101@PCC-5	10	9.79	/	0.53	/	0.054
Surface etched MIL-101@PCC-5	10	16.78	/	0.29	/	0.017

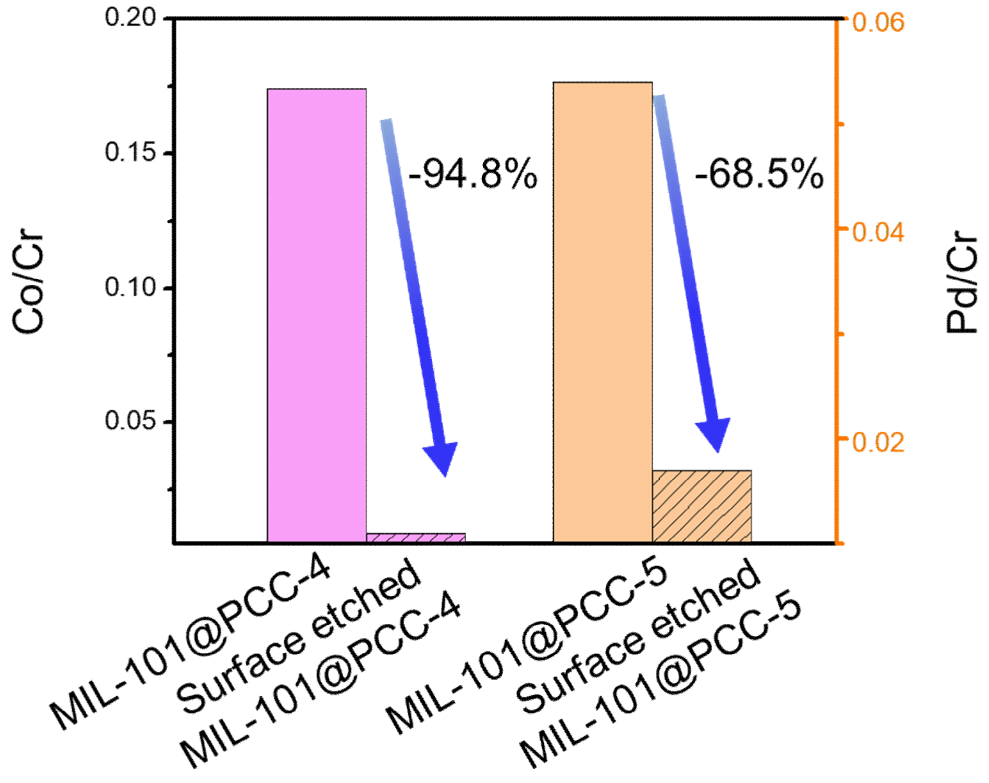

Supplementary Figure 75. The ratio Co/Cr of **MIL-101@PCC-4** before and after acid treatment, and the ratio Pd/Cr of **MIL-101@PCC-5** before and after acid treatment.

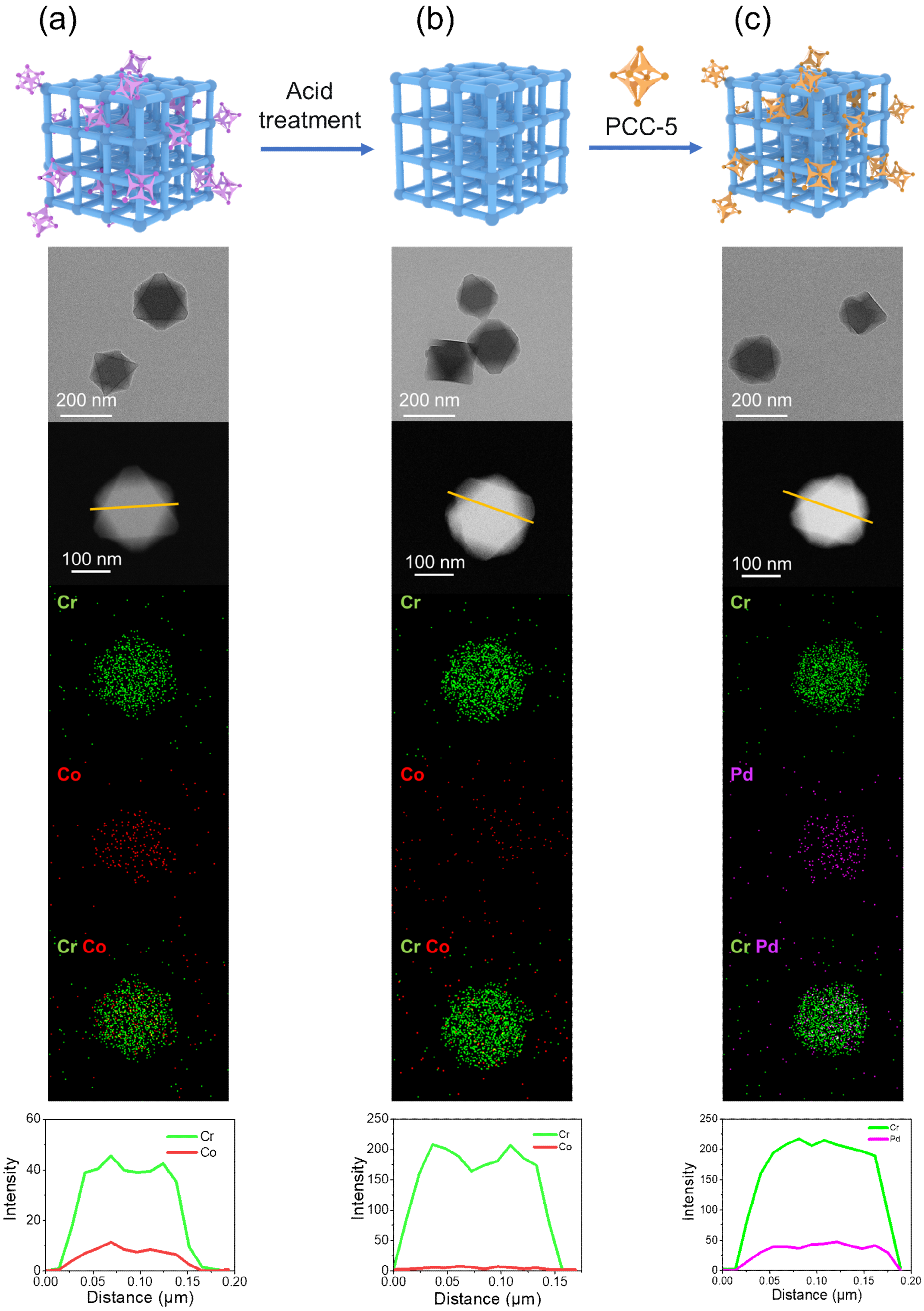

Supplementary Figure 76. A schematic representation, STEM images, elemental mapping, and linear scanning analysis of the (a) **PCN-222@PCC-4**, (b) **PCN-222@PCC-4** after acid treatment, and (c) reversible modification of **PCC-5** on **PCN-222**.

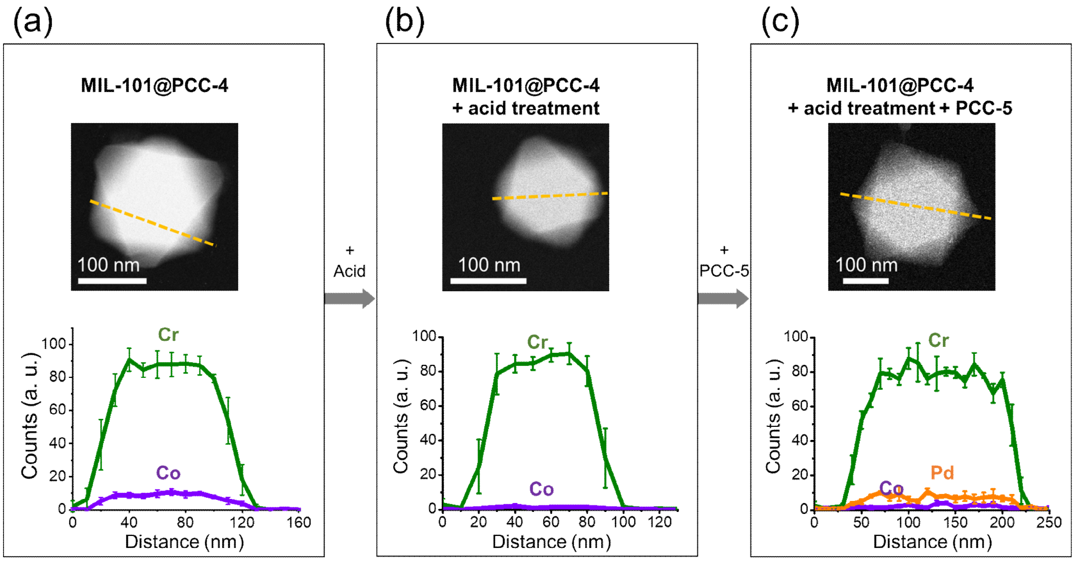

Supplementary Figure 77. STEM images and linear scanning analysis of the (a) **PCN-222@PCC-4**, (b) **PCN-222@PCC-4** after acid treatment, and (c) reversible modification of **PCC-5** on **PCN-222**. Data are presented as the mean ± SD.

We further used MD simulation to illustrate the PCC coverage on the MIL-101 surface to provide direct visualization of the surface coating mode. As demonstrated by MD simulation, both PCC-4 and PCC-5 molecules solely attach to the exterior surface of MIL-101 but not inside pores, due to the steric hindrance and electrostatic repulsion (Supplementary Fig. 78). The spacing filling mode of the MIL-101 particle on the (111) facet revealed that the molecular cages are mostly attaching to the surface but not getting into the open channels.

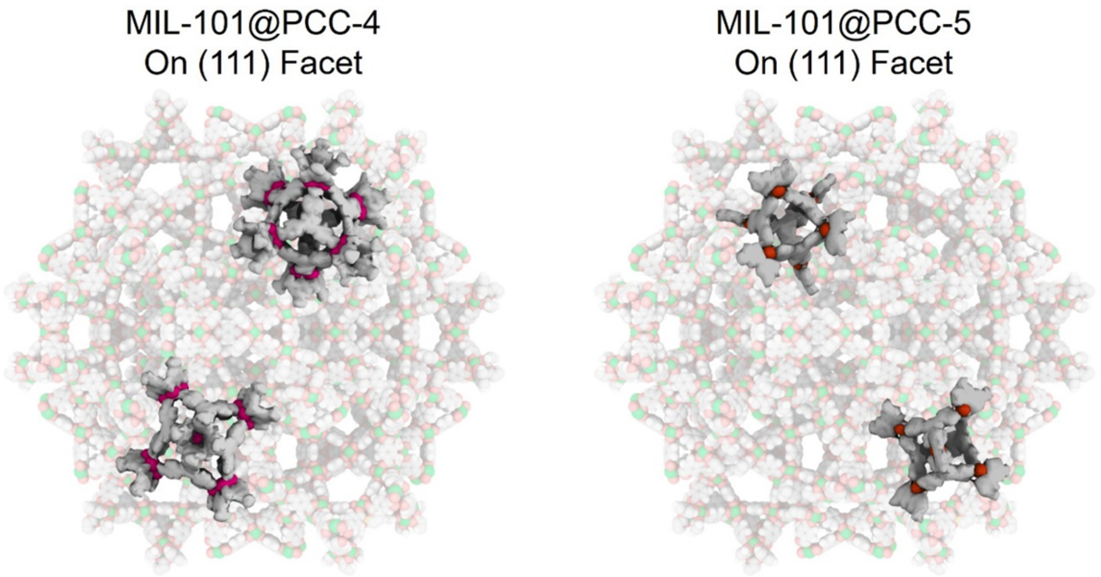

Supplementary Figure 78. Simulation structure of **MIL-101@PCC-4** and **MIL-101@PCC-5** on the (111) facet.
