# Supplementary material for: A cage-on-MOF strategy to coordinatively functionalize mesoporous MOFs for manipulating selectivity in adsorption and catalysis
Source: Nat Commun. 2023 Aug 26;14:5223. doi: 10.1038/s41467-023-40973-9 (PMC10460432; doi:10.1038/s41467-023-40973-9)
Supplement: Supplementary file 3 — Description of Additional Supplementary Files [file 41467_2023_40973_MOESM3_ESM.pdf]

Description of Additional Supplementary Files

Supplementary Data 1
Description: Crystallographic data for PCC-4.
